# Variation in alternative splicing across human tissues

**DOI:** 10.1186/gb-2004-5-10-r74

**Published:** 2004-09-13

**Authors:** Gene Yeo, Dirk Holste, Gabriel Kreiman, Christopher B Burge

**Affiliations:** 1 Department of Biology, Center for Biological and Computational Learning, Massachusetts Institute of Technology, Cambridge, MA 02319, USA; 2 Department of Brain and Cognitive Sciences, Center for Biological and Computational Learning, Massachusetts Institute of Technology, Cambridge, MA 02319, USA

## Abstract

**Background:**

Alternative pre-mRNA splicing (AS) is widely used by higher eukaryotes to generate different protein isoforms in specific cell or tissue types. To compare AS events across human tissues, we analyzed the splicing patterns of genomically aligned expressed sequence tags (ESTs) derived from libraries of cDNAs from different tissues.

**Results:**

Controlling for differences in EST coverage among tissues, we found that the brain and testis had the highest levels of exon skipping. The most pronounced differences between tissues were seen for the frequencies of alternative 3' splice site and alternative 5' splice site usage, which were about 50 to 100% higher in the liver than in any other human tissue studied. Quantifying differences in splice junction usage, the brain, pancreas, liver and the peripheral nervous system had the most distinctive patterns of AS. Analysis of available microarray expression data showed that the liver had the most divergent pattern of expression of serine-arginine protein and heterogeneous ribonucleoprotein genes compared to the other human tissues studied, possibly contributing to the unusually high frequency of alternative splice site usage seen in liver. Sequence motifs enriched in alternative exons in genes expressed in the brain, testis and liver suggest specific splicing factors that may be important in AS regulation in these tissues.

**Conclusions:**

This study distinguishes the human brain, testis and liver as having unusually high levels of AS, highlights differences in the types of AS occurring commonly in different tissues, and identifies candidate *cis*-regulatory elements and *trans*-acting factors likely to have important roles in tissue-specific AS in human cells.

## Abstract 2

Analysis of the alternative splicing patterns of genomically aligned ESTs revealed that human brain, testis and liver have unusually high levels of alternative splicing and identified candidate *cis*-acting factors likely to play important roles in tissue-specific alternative splicing in human cells.

## Background

The differentiation of a small number of cells in the developing embryo into the hundreds of cell and tissue types present in a human adult is associated with a multitude of changes in gene expression. In addition to many differences between tissues in transcriptional and translational regulation of genes, alternative pre-mRNA splicing (AS) is also frequently used to regulate gene expression and to generate tissue-specific mRNA and protein isoforms [1-5]. Between one-third and two-thirds of human genes are estimated to undergo AS [6-11] and the disruption of specific AS events has been implicated in several human genetic diseases [12]. The diverse and important biological roles of alternative splicing have led to significant interest in understanding its regulation.

Insights into the regulation of AS have come predominantly from the molecular dissection of individual genes (reviewed in [1,12]). Prominent examples include the tissue-specific splicing of the c-*src *N1 exon [13], cancer-associated splicing of the *CD44 *gene [14] and the alternative splicing cascade involved in *Drosophila melanogaster *sex determination [15]. Biochemical studies of these and other genes have described important classes of *trans*-acting splicing-regulatory factors, implicating members of the ubiquitously expressed serine/arginine-rich protein (SR protein) and heterogeneous nuclear ribonucleoprotein (hnRNP) families, and tissue-specific factors including members of the CELF [16] and NOVA [17] families of proteins, as well as other proteins and protein families, in control of specific splicing events. A number of *cis*-regulatory elements in exons or introns that play key regulatory roles have also been identified, using a variety of methods including site-directed mutagenesis, systematic evolution of ligands by exponential enrichment (SELEX) and computational approaches [18-22]. In addition, DNA microarrays and polymerase colony approaches have been developed for higher-throughput analysis of alternative mRNA isoforms [23-26] and a cross-linking/immunoprecipitation strategy (CLIP) has been developed for systematic detection of the RNAs bound by a given splicing factor [27]. These new methods suggest a path towards increasingly parallel experimental analysis of splicing regulation.

From another direction, the accumulation of large databases of cDNA and expressed sequence tag (EST) sequences has enabled large-scale computational studies, which have assessed the scope of AS in the mammalian transcriptome [3,8,10,28]. Other computational studies have analyzed the tissue specificity of AS events and identified sets of exons and genes that exhibit tissue-biased expression [29,30]. However, a number of significant questions about tissue-specific alternative splicing have not yet been comprehensively addressed. Which tissues have the highest and lowest proportions of alternative splicing? Do tissues differ in their usage of different AS types, such as exon skipping, alternative 5' splice site choice or alternative 3' splice site choice? Which tissues are most distinct from other tissues in the spectrum of alternative mRNA isoforms they express? And to what extent do expression levels of known splicing factors explain AS patterns in different tissues?

Here, we describe an initial effort to answer these questions using a large-scale computational analysis of ESTs derived from about two dozen human tissues, which were aligned to the assembled human genome sequence to infer patterns of AS occurring in thousands of human genes. Our results distinguish specific tissues as having high levels and distinctive patterns of AS, identify pronounced differences between the proportions of alternative 5' splice site and alternative 3' splice site usage between tissues, and predict candidate *cis*-regulatory elements and *trans*-acting factors involved in tissue-specific AS.

## Results and discussion

### Variation in the levels of alternative splicing in different human tissues

**Figure 1:**
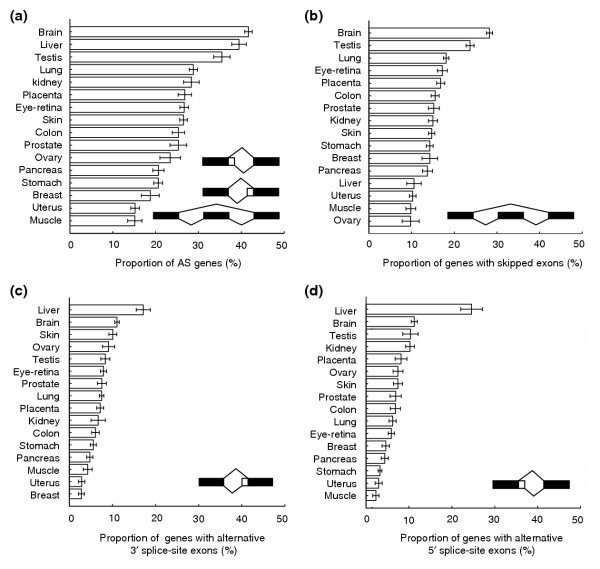
Levels of alternative splicing in 16 human tissues with moderate or high EST sequence coverage. Horizontal bars show the average fraction of alternatively spliced (AS) genes of each splicing type (and estimated standard deviation) for random samplings of 20 ESTs per gene from each gene with ≥ 20 aligned EST sequences derived from a given human tissue. The different splicing types are schematically illustrated in each subplot. **(a) **Fraction of AS genes containing skipped exons, alternative 3' splice site exons (A3Es) or 5' splice site exons (A5Es), **(b) **fraction of AS genes containing skipped exons, **(c) **fraction of AS genes containing A3Es, **(d) **fraction of AS genes containing A5Es.

Alternative splicing events are commonly distinguished in terms of whether mRNA isoforms differ by inclusion or exclusion of an exon, in which case the exon involved is referred to as a 'skipped exon' (SE) or 'cassette exon', or whether isoforms differ in the usage of a 5' splice site or 3' splice site, giving rise to alternative 5' splice site exons (A5Es) or alternative 3' splice site exons (A3Es), respectively (depicted in Figure 1). These descriptions are not necessarily mutually exclusive; for example, an exon can have both an alternative 5' splice site and an alternative 3' splice site, or have an alternative 5' splice site or 3' splice site but be skipped in other isoforms. A fourth type of alternative splicing, 'intron retention', in which two isoforms differ by the presence of an unspliced intron in one transcript that is absent in the other, was not considered in this analysis because of the difficulty in distinguishing true intron retention events from contamination of the EST databases by pre-mRNA or genomic sequences. The presence of these and other artifacts in EST databases are important caveats to any analysis of EST sequence data. Therefore, we imposed stringent filters on the quality of EST to genomic alignments used in this analysis, accepting only about one-fifth of all EST alignments obtained (see Materials and methods).

To determine whether differences occur in the proportions of these three types of AS events across human tissues, we assessed the frequencies of genes containing skipped exons, alternative 3' splice site exons or alternative 5' splice site exons for 16 human tissues (see Figure 1 for the list of tissues) for which sufficiently large numbers of EST sequences were available. Because the availability of a larger number of ESTs derived from a gene increases the chance of observing alternative isoforms of that gene, the proportion of AS genes observed in a tissue will tend to increase with increasing EST coverage of genes [10,31]. Since the number of EST sequences available differs quite substantially among human tissues (for example, the dbEST database contains about eight times more brain-derived ESTs than heart-derived ESTs), in order to compare the proportion of AS in different tissues in an unbiased way, we used a sampling strategy that ensured that all genes/tissues studied were represented by equal numbers of ESTs.

It is important to point out that our analysis does not make use of the concept of a canonical transcript for each gene because it is not clear that such a transcript could be chosen objectively or that this concept is biologically meaningful. Instead, AS events are defined only through pairwise comparison of ESTs.

Our objective was to control for differences in EST abundance across tissues while retaining sufficient power to detect a reasonable fraction of AS events. For each tissue we considered genes that had at least 20 aligned EST sequences derived from human cDNA libraries specific to that tissue ('tissue-derived' ESTs). For each such gene, a random sample of 20 of these ESTs was chosen (without replacement) to represent the splicing of the given gene in the given human tissue. For the gene and tissue combinations included in this analysis, the median number of EST sequences per gene was not dramatically different between tissues, ranging from 25 to 35 (see Additional data file 1). The sampled ESTs for each gene were then compared to each other to identify AS events occurring within the given tissue (see Materials and methods). The random sampling was repeated 20 times and the mean fraction of AS genes observed in these 20 trials was used to assess the fraction of AS genes for each tissue (Figure 1a). Different random subsets of a relatively large pool will have less overlap in the specific ESTs chosen (and therefore in the specific AS events detected) than for random subsets of a smaller pool of ESTs, and increased numbers of ESTs give greater coverage of exons. However, there is no reason that the expected number of AS events detected per randomly sampled subset should depend on the size of the pool the subset was chosen from. While the error (standard deviation) of the measured AS frequency per gene should be lower when restricting to genes with larger minimum pools of ESTs, such a restriction would not change the expected value. Unfortunately, the reduction in error of the estimated AS frequency per gene is offset by an increase in the expected error of the tissue-level AS frequency resulting from the use of fewer genes. The inclusion of all genes with at least 20 tissue-derived ESTs represents a reasonable trade-off between these factors.

The human brain had the highest fraction of AS genes in this analysis (Figure 1a), with more than 40% of genes exhibiting one or more AS events, followed by the liver and testis. Previous EST-based analyses have identified high proportions of splicing in human brain and testis tissues [29,30,32]. These studies did not specifically control for the highly unequal representation of ESTs from different human tissues. As larger numbers of ESTs increase the chance of observing a larger fraction of the expressed isoforms of a gene, the number of available ESTs has a direct impact on estimated proportions of AS, as seen previously in analyses comparing the levels of AS in different organisms [31]. Thus, the results obtained in this study confirm that the human brain and testis possess an unusually high level of AS, even in the absence of EST-abundance advantages over other tissues. We also observe a high level of AS in the human liver, a tissue with much lower EST coverage, where higher levels of AS have been previously reported in cancerous cells [33,34]. Human muscle, uterus, breast, stomach and pancreas had the lowest levels of AS genes in this analysis (less than 25% of genes). Lowering the minimum EST count for inclusion in this analysis from 20 to 10 ESTs, and sampling 10 (out of 10 or more) ESTs to represent each gene in each tissue, did not alter the results qualitatively (data not shown).

### Differences in the levels of exon skipping in different tissues

Alternatively spliced genes in this analysis exhibited on average between one and two distinct AS exons. Analyzing the different types of AS events separately, we found that the human brain and testis had the highest levels of skipped exons, with more than 20% of genes containing SEs (Figure 1b). The high level of skipped exons observed in the brain is consistent with previous analyses [29,30,32]. At the other extreme, the human ovary, muscle, uterus and liver had the lowest levels of skipped exons (about 10% of genes).

**Figure 2:**
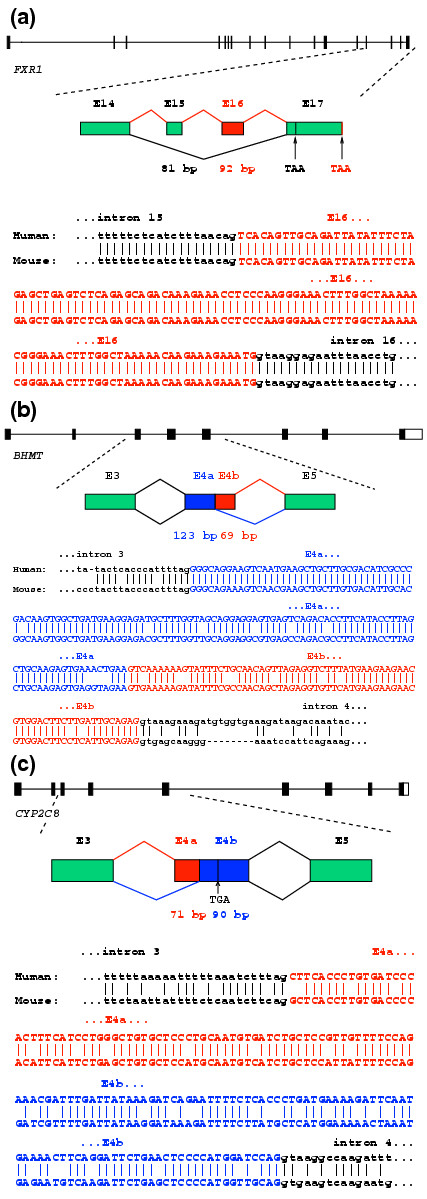
Examples of tissue-specific AS events in human genes with evidence of splice conservation in orthologous mouse genes. **(a) **Human fragile X mental retardation syndrome-related (*FXR1*) gene splicing detected in brain-derived EST sequences. *FXR1 *exhibited two alternative mRNA isoforms differing by skipping/inclusion of exons E15 and E16. Exclusion of E16 creates a shift in the reading-frame, which is predicted to result in an altered and shorter carboxy terminus. The exon-skipping event is conserved in the mouse ortholog of the human *FXR1 *gene, and both isoforms were detected in mouse brain-derived ESTs. **(b) **Human betaine-homocysteine *S*-methyltransferase (*BHMT*) gene splicing detected in liver-derived ESTs. *BHMT *exhibited two alternative isoforms differing by alternative 5' splice site usage in exon E4. Sequence comparisons indicate that the exon and splice site sequences involved in both alternative 5' splice site exon events are conserved in the mouse ortholog of the human *BHMT *gene. **(c) **Human cytochrome P450 2C8 (*CYP2C8*) gene splicing. *CYP2C8 *exhibited two alternative mRNA isoforms differing in the 3' splice site usage for exon E4 (detected in ESTs derived from several tissues), where the exclusion of a 71-base sequence creates a premature termination codon in exon E4b. Exons and splice sites involved in the AS event are conserved in the mouse ortholog of *CYP2C8*.

An example of a conserved exon-skipping event observed in human and mouse brain tissue is shown in Figure 2a for the human fragile X mental retardation syndrome-related (*FXR1*) gene [35,36]. In this event, skipping of the exon alters the reading frame of the downstream exon, presumably leading to production of a protein with an altered and truncated carboxy terminus. The exon sequence is perfectly conserved between the human and mouse genomes, as are the 5' splice site and 3' splice site sequences (Figure 2a), suggesting that this AS event may have an important regulatory role [37-39].

### Differences in the levels of alternative splice site usage in different tissues

Analyzing the proportions of AS events involving the usage of A5Es and A3Es revealed a very different pattern (Figure 1c,d). Notably, the fraction of genes containing A3Es was more than twice as high in the liver as in any other human tissue studied (Figure 1d), and the level of A5Es was also about 40-50% higher in the liver than in any other tissue (Figure 1c). The tissue with the second highest level of alternative usage for both 5' splice sites and 3' splice sites was the brain. Another group of human tissues including muscle, uterus, breast, pancreas and stomach - similar to the low SE frequency group above - had the lowest level of A5Es and A3Es (less than 5% of genes in each category). Thus, a picture emerges in which certain human tissues such as muscle, uterus, breast, pancreas and stomach, have low levels of AS of all types, whereas other tissues, such as the brain and testis, have relatively high levels of AS of all types and the liver has very high levels of A3Es and A5Es, but exhibits only a modest level of exon skipping. To our knowledge, this study represents the first systematic analysis of the proportions of different types of AS events occurring in different tissues. Repeating the analyses by removing ESTs from disease-associated tissue libraries, using available library classifications [40], gave qualitatively similar results (see Additional data files 2, 3, and 4). These data show that ESTs derived from diseased tissues show modestly higher frequencies of exon skipping, but the relative rankings of tissues remain similar. The fractions of genes containing A5Es and A3Es were not changed substantially when diseased-tissue ESTs were excluded.

From the set of genes with at least 20 human liver-derived ESTs, this analysis identified a total of 114 genes with alternative 5' splice site and/or 3' splice site usage in the liver. Those genes in this set that were named, annotated and for which the consensus sequences of the alternative splice sites were conserved in the orthologous mouse gene (see Materials and methods) are listed in Table 1. Of course, conservation of splice sites alone is necessary, but not sufficient by itself, to imply conservation of the AS event in the mouse. Many essential liver metabolic and detoxifying enzyme-coding genes appear on this list, including enzymes involved in sugar metabolism (for example, *ALDOB*, *IDH1*), protein and amino acid metabolism (for example, *BHMT, CBP2*, *TDO2*, *PAH*, *GATM*), detoxification or breakdown of drugs and toxins (for example, *GSTA3*, *CYP3A4*, *CYP2C8*).

Sequences and splicing patterns for two of these genes for which orthologous mouse exons/genes and transcripts could be identified - the genes *BHMT *and *CYP2C8 *- are shown in detail in Figure 2b,c. In the event depicted for *BHMT*, the exons involved are highly conserved between the human and mouse orthologs (Figure 2b), consistent with the possibility that the splicing event may have a (conserved) regulatory role. This AS event preserves the reading frame of downstream exons, so the two isoforms are both likely to produce functional proteins, differing by the insertion/deletion of 23 amino acids. In the event depicted for *CYP2C8*, usage of an alternative 3' splice site removes 71 nucleotides, shifting the reading frame and leading to a premature termination codon in the exon (Figure 2c). In this case, the shorter alternative transcript is a potential substrate for nonsense-mediated decay [41,42] and the AS event may be used to regulate the level of functional mRNA/protein produced.

### Differences in splicing factor expression between tissues

**Figure 3:**
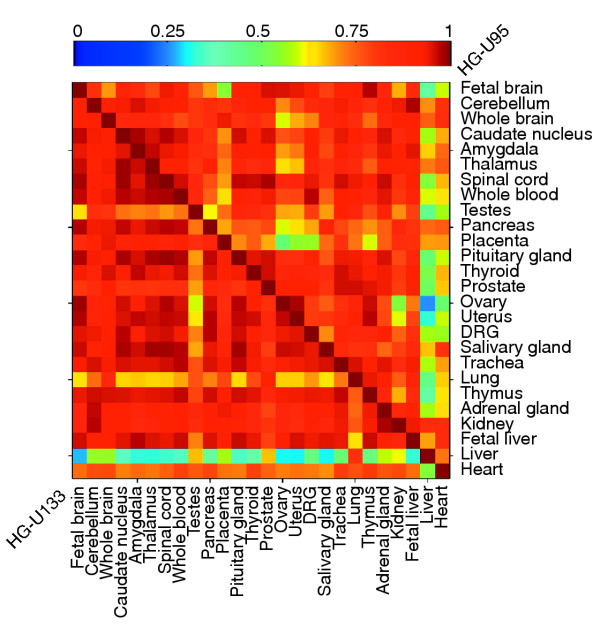
Correlation of mRNA expression levels of 20 known splicing factors (see Materials and methods) across 26 human tissues (lower diagonal: data from Affymetrix HU-133A DNA microarray experiment [45]; upper diagonal: data from Affymetrix HU-95A DNA microarray experiment [43]). Small squares are colored to represent the extent of the correlation between the mRNA expression patterns of the 20 splicing factor genes in each pair of tissues (see scale at top of figure).

To explore the differences in splicing factor expression in different tissues, available mRNA expression data was obtained from two different DNA microarray studies [43-45]. For this *trans*-factor analysis, we obtained a list of 20 splicing factors of the SR, SR-related and hnRNP protein families from proteomic analyses of the human spliceosome [46-48] (see Materials and methods for the list of genes). The variation in splicing-factor expression between pairs of tissues was studied by computing the Pearson (product-moment) correlation coefficient (*r*) between the 20-dimensional vectors of splicing-factor expression values between all pairs of 26 human tissues. The DNA microarray studies analyzed 10 tissues in addition to the 16 previously studied (Figure 3). A low value of *r *between a pair of tissues indicates a low degree of concordance in the relative mRNA expression levels across this set of splicing factors, whereas a high value of *r *indicates strong concordance.

While most of the tissues examined showed a very high degree of correlation in the expression levels of the 20 splicing factors studied (typically with *r *> 0.75; Figure 3), the human adult liver was clearly an outlier, with low concordance in splicing-factor expression to most other tissues (typically *r *< 0.6, and often much lower). The unusual splicing-factor expression in the human liver was seen consistently in data from two independent DNA microarray studies using different probe sets (compare the two halves of Figure 3). The low correlation observed between liver and other tissues in splicing factor expression is statistically significant even relative to arbitrary collections of 20 genes (see Additional data file 8). Examining the relative levels of specific splicing factors in the human adult liver versus other tissues, the relative level of SRp30c message was consistently higher in the liver and the relative levels of SRp40, hnRNP A2/B2 and Srp54 messages were consistently lower. A well established paradigm in the field of RNA splicing is that usage of alternative splice sites is often controlled by the relative concentrations of specific SR proteins and hnRNP proteins [49-52]. This functional antagonism between particular SR and hnRNP proteins is often due to competition for binding of nearby sites on pre-mRNAs [49,53,54]. Therefore, it seems likely that the unusual patterns of expression seen in the human adult liver for these families of splicing factors may contribute to the high level of alternative splice site usage seen in this tissue. It is also interesting that splicing-factor expression in the human fetal liver is highly concordant with most other tissues, but has low concordance with the adult liver (Figure 3). This observation suggests that substantial changes in splicing-factor expression may occur during human liver development, presumably leading to a host of changes in the splicing patterns of genes expressed in human liver. Currently available EST data were insufficient to allow systematic analysis of the patterns of AS in fetal relative to adult liver.

An important caveat to these results is that the DNA microarray data used in this analysis measure mRNA expression levels rather than protein levels or activities. The relation between the amount of mRNA expressed from a gene and the concentration of the corresponding protein has been examined previously in several studies in yeast as well as in human and mouse liver tissues [55-58]. These studies have generally found that mRNA expression levels correlate positively with protein concentrations, but with fairly wide divergences for a significant fraction of genes.

### Over-represented motifs in alternative exons in the human brain, testis and liver

The unusually high levels of alternative splicing seen in the human brain, testis and liver prompted us to search for candidate tissue-specific splicing regulatory motifs in AS exons in genes expressed in each of these tissues. Using a procedure similar to Brudno *et al*. [59], sequence motifs four to six bases long that were significantly enriched in exons skipped in AS genes expressed in the human brain relative to constitutive exons in genes expressed in the brain were identified. These sequences were then compared to each other and grouped into seven clusters, each of which shared one or two four-base motifs (Table 2). The motifs in cluster BR1 (CUCC, CCUC) resemble the consensus binding site for the polypyrimidine tract-binding protein (PTB), which acts as a repressor of splicing in many contexts [60-63]. A similar motif (CNCUCCUC) has been identified in exons expressed specifically in the human brain [29]. The motifs in cluster BR7 (containing UAGG) are similar to the high-affinity binding site UAGGG [A/U], identified for the splicing repressor protein hnRNP A1 by SELEX experiments [64]. The consensus sequences for the remaining clusters BR2 to BR6 (GGGU, UGGG, GGGA, CUCA, UAGC, respectively), as well as BR7, all resembled motifs identified in a screen for exonic splicing silencers (ESSs) in cultured human cells (Z. Wang and C.B.B., unpublished results), suggesting that most or all of the motifs BR1 to BR7 represent sequences directly involved in mediating exon skipping. In particular, G-rich elements, which are known to act as intronic splicing enhancers [65,66], may function as silencers of splicing when present in an exonic context.

A comparison of human testis-derived skipped exons to exons constitutively included in genes expressed in the testis identified only a single cluster of sequences, TE1, which share the tetramer UAGG. Enrichment of this motif, common to the brain-specific cluster BR7, suggests a role for regulation of exon skipping by hnRNP A1 - or a *trans-*acting factor with similar binding preferences - in the testis.

Alternative splice site usage gives rise to two types of exon segments - the 'core' portion common to both splice forms and the 'extended' portion that is present only in the longer isoform. Two clusters of sequence motifs enriched in the core sequences of A5Es in genes expressed in the liver relative to the core segments of A5Es resulting from alignments of non-liver-derived ESTs were identified - LI1 and LI2. Both are adenosine-rich, with consensus tetramers AAAC and UAAA, respectively. The former motif matches a candidate ESE motif identified previously using the computational/experimental RESCUE-ESE approach (motif 3F with consensus [AG]AA [AG]C) [19]. The enrichment of a probable ESE motif in exons exhibiting alternative splice site usage in the liver is consistent with the model that such splicing events are often controlled by the relative levels of SR proteins (which bind many ESEs) and hnRNP proteins. Insufficient data were available for the analysis of motifs in the extended portions of liver A5Es (which tend to be significantly shorter than the core regions) or for the analysis of liver A3Es.

### A measure of dissimilarity between mRNA isoforms

**Figure 4:**
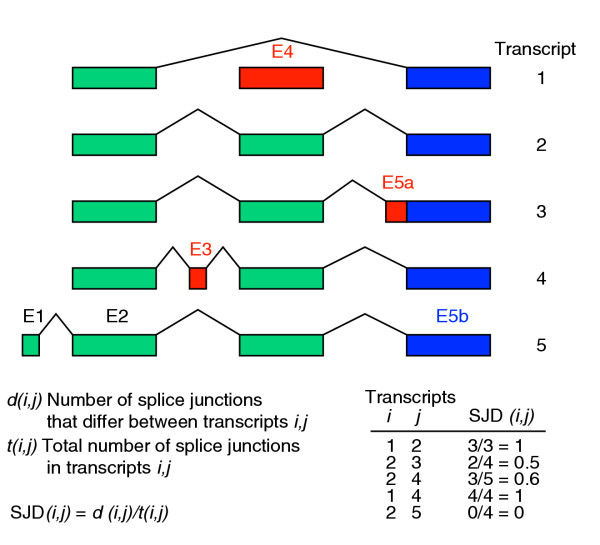
Computation of splice junction difference ratio (SJD). The SJD value for a pair of transcripts is computed as the number of splice junctions in each transcript that are not represented in the other transcript, divided by the total number of splice junctions in the two transcripts, in both cases considering only those splice junctions that occur in portions of the two transcripts that overlap (see Materials and methods for details). SJD value calculations for different combinations of the transcripts shown in the upper part of the figure are also shown.

To quantify the differences in splicing patterns between mRNAs or ESTs derived from a gene locus, a new measure called the splice junction difference ratio (SJD) was developed. For any pair of mRNAs/ESTs that align to overlapping portions of the same genomic locus, the SJD is defined as the proportion of splice junctions present in both transcripts that differ between them, including only those splice junctions that occur in regions of overlap between the transcripts (Figure 4). The SJD varies between zero and one, with a value of zero for any pair of transcripts that have identical splice junctions in the overlapping region (for example, transcripts 2 and 5 in Figure 4, or for two identical transcripts), and has a value of 1.0 for two transcripts whose splice junctions are completely different in the regions where they overlap (for example, transcripts 1 and 2 in Figure 4). For instance, transcripts 2 and 3 in Figure 4 differ in the 3' splice site used in the second intron, yielding an SJD value of 2/4 = 0.5, whereas transcripts 2 and 4 differ by skipping/inclusion of an alternative exon, which affects a larger fraction of the introns in the two transcripts and therefore yields a higher SJD value of 3/5 = 0.6.

The SJD value can be generalized to compare splicing patterns between two sets of transcripts from a gene - for example, to compare the splicing patterns of the sets of ESTs derived from two different tissues. In this case, the SJD is defined by counting the number of splice junctions that differ between all pairs of transcripts (*i*, *j*), with transcript *i *coming from set 1 (for example, heart-derived ESTs), and transcript *j *coming from set 2 (for example, lung-derived ESTs), and dividing this number by the total number of splice junctions in all pairs of transcripts compared, again considering only those splice junctions that occur in regions of overlap between the transcript pairs considered. Note that this definition has the desirable property that pairs of transcripts that have larger numbers of overlapping splice junctions contribute more to the total than transcript pairs that overlap less. As an example of the splice junction difference between two sets of transcripts, consider the set *S*1, consisting of transcripts (1,2) from Figure 4, and set *S*2, consisting of transcripts (3,4) from Figure 4. Using the notation introduced in Figure 4, SJD(*S*1,*S*2) = *d*(*S*1,*S*2) / *t*(*S*1,*S*2) = [*d*(1,3) + *d*(1,4) + *d*(2,3) + *d*(2,4)]/ [*t*(1,3) +*t*(1,4) + *t*(2,3) + *t*(2,4)] = [3 + 4 + 2 + 3]/ [3 + 4 + 4 + 5] = 12/16 = 0.75, reflecting a high level of dissimilarity between the isoforms in these sets, whereas the SJD falls to 0.57 for the more similar sets *S*1 = transcripts (1,2) versus *S*3 = transcripts (2,3). Note that in cases where multiple similar/identical transcripts occur in a given set, the SJD measure effectively weights the isoforms by their abundance, reflecting an average dissimilarity when comparing randomly chosen pairs of transcripts from the two tissues. For example, the SJD computed for the set *S*4 = (1,2,2,2,2), that is, one transcript aligning as transcript 1 in Figure 4 and four transcripts aligning as transcript 2, and the set *S*5 = (2,2,2,2,3) is 23/95 = 0.24, substantially lower than the SJD value for sets *S*1 versus *S*3 above, reflecting the higher fraction of identically spliced transcripts between sets *S*4 and *S*5.

### Global comparison of splicing patterns between tissues

To make a global comparison of patterns of splicing between two different human tissues, a tissue-level SJD value was computed by comparing the splicing patterns of ESTs from all genes for which at least one EST was available from cDNA libraries representing both tissues. The 'inter-tissue' SJD value is then defined as the ratio of the sum of *d*(*S*_A_,*S*_B_) values for all such genes, divided by the sum of *t*(*S*_A_,*S*_B_) values for all of these genes, where *S*_A _and *S*_B _refer to the set of ESTs for a gene derived from tissues A and B, respectively, and *d*(*S*_A_,*S*_B_) and *t*(*S*_A_,*S*_B_) are defined in terms of comparison of all pairs of ESTs from the two sets as described above. This analysis uses all available ESTs for each gene in each tissue (rather than samples of a fixed size). A large SJD value between a pair of tissues indicates that mRNA isoforms of genes expressed in the two tissues tend to be more dissimilar in their splicing patterns than is the case for two tissues with a smaller inter-tissue SJD value. This definition puts greater weight on those genes for which more ESTs are available.

**Figure 5:**
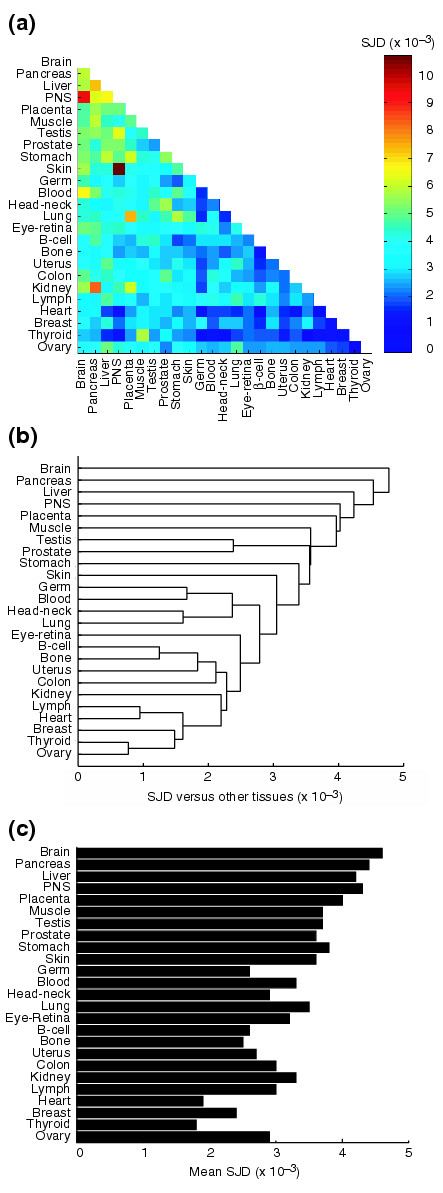
Comparison of alternative mRNA isoforms across 25 human tissues. **(a) **Color-coded representation of *SJD *values between pairs of tissues (see Figure 4 and Materials and methods for definition of SJD). **(b) **Hierarchical clustering of SJD values using average-linkage clustering. Groups of tissues in clusters with short branch lengths (for example, thyroid/ovary, B-cell/bone) have highly similar patterns of AS. **(c) **Mean SJD values (versus other 24 tissues) for each tissue.

The SJD values were then used to globally assess tissue-level differences in alternative splicing. A set of 25 human tissues for which at least 20,000 genomically aligned ESTs were available was compiled for this comparison (see Materials and methods) and the SJD values were then computed between all pairs of tissues in this set (Figure 5a). A clustering of human tissues on the basis of their inter-tissue SJD values (Figure 5b) identified groups of tissues that cluster together very closely (for example, the ovary/thyroid/breast cluster, the heart/lymph cluster and the bone/B-cell cluster), while other tissues including the brain, pancreas, liver, peripheral nervous system (PNS) and placenta occur as outgroups. These results complement a previous clustering analysis based on data from microarrays designed to detect exon skipping [24]. Calculating the mean SJD value for a given tissue when compared to the remaining 24 tissues (Figure 5c) identified a set of human tissues including the ovary, thyroid, breast, heart, bone, B-cell, uterus, lymph and colon that have 'generic' splicing patterns which are more similar to most other tissues. As expected, many of these tissues with generic splicing patterns overlap with the set of tissues that have low levels of AS (Figure 1). On the other hand, another group of tissues including the human brain, pancreas, liver and peripheral nervous system, have highly 'distinctive' splicing patterns that differ from most other tissues (Figure 5c). Many of these tissues were identified as having high proportions of AS in Figure 1. Taken together, these observations suggest that specific human tissues such as the brain, testis and liver, make more extensive use of AS in gene regulation and that these tissues have also diverged most from other tissues in the set of spliced isoforms they express. Although we are not aware of reliable, quantitative data on the relative abundance of different cell types in these tissues, a greater diversity of cell types is likely to contribute to higher SJD values for many of these tissues.

## Conclusions

The systematic analysis of transcripts generated from the human genome is just beginning, but promises to deepen our understanding of how changes in the program of gene expression contribute to development and differentiation. Here, we have observed pronounced differences between human tissues in the set of alternative mRNA isoforms that they express. Because our approach normalizes the EST coverage per gene in each tissue, there is higher confidence that these differences accurately reflect differences in splicing patterns between tissues. As human tissues are generally made up of a mixture of cell types, each of which may have its own unique pattern of gene expression and splicing, it will be important in the future to develop methods for systematic analysis of transcripts in different human cell types.

Understanding the mechanisms and regulatory consequences of AS will require experimental and computational analyses at many levels. At its core, AS involves the generation of alternative transcripts mediated by interactions between *cis*-regulatory elements in exons or introns and *trans*-acting splicing factors. The current study has integrated these three elements, inferring alternative transcripts from EST-genomic alignments, identifying candidate regulatory sequence motifs enriched in alternative exons from different tissues, and analyzing patterns of splicing-factor expression in different tissues. Our results emphasize differences in the frequencies of exon skipping versus alternative splice site usage in different tissues and highlight the liver, brain and testis as having particularly high levels of AS, supporting the idea that tissue-regulated AS plays important roles in the differentiation of these tissues. The high levels of alternative splice site usage in the liver may relate to the unusual patterns of splicing-factor expression observed in the adult liver, suggesting aspects of developmental regulation of AS at the tissue level. Obtaining a more comprehensive picture of AS will require the integration of additional types of data upstream and downstream of these core interactions. Upstream, splicing factors themselves may be differentially regulated in different tissues or in response to different stimuli at the level of transcription, splicing, or translation, and are frequently regulated by post-translational modifications such as phosphorylation, so systematic measurements of splicing factor levels and activities will be required. Downstream, AS may affect the stability of alternative transcripts (for example, in cases of messages subject to nonsense-mediated mRNA decay), and frequently alters functional properties of the encoded proteins, so systematic measurements of AS transcript and protein isoforms and functional assays will also be needed to fully understand the regulatory consequences of AS events. Ultimately, it will be important to place regulatory events involving AS into the context of regulatory networks involving control at the levels of transcription, translation and post-translational modifications.

## Materials and methods

### Data and resources

Chromosome assemblies of the human genome (hg13) were obtained from public databases [67]. Transcript databases included approximately 94,000 human cDNA sequences obtained from GenBank (release 134.0, gbpri and gbhtc categories), and approximately 5 million human expressed sequence tags (ESTs) from dbEST (repository 02202003). Human ESTs were designated according to their cDNA library source (in total about 800) into different tissue types. Pertinent information about cDNA libraries and the corresponding human tissue or cell line was extracted from dbEST and subsequently integrated with library information retrieved from the Mammalian Gene Collection Initiative (MGC) [68], the Integrated Molecular Analysis of Gene Expression Consortium (IMAGE) [69] and the Cancer Genome Anatomy Project (CGAP) [70]. Library information obtained from MGC, IMAGE and CGAP is provided in Additional data file 5.

### Genome annotation by alignment of spliced transcripts

The GENOA genome annotation script [71] was used to align spliced cDNA and EST sequences to the human genome. GENOA uses BLASTN to detect significant blocks of identity between repeat-masked cDNA sequences and genomic DNA, and then aligns cDNAs to the genomic loci identified by BLASTN using the spliced-alignment algorithm MRNAVSGEN [71]. This algorithm is similar in concept to SIM4 [72] but was developed specifically to align high-quality cDNAs rather than ESTs and thus requires higher alignment quality (at least 93% identity) and consensus terminal dinucleotides at the ends of all introns (that is, GT..AG, GC..AG or AT..AC). EST sequences were aligned using SIM4 to those genomic regions that had aligned cDNAs. Stringent alignment criteria were imposed: ESTs were required to overlap cDNAs (so that all the genes studied were supported by at least one cDNA-genomic alignment); the first and last aligned segments of ESTs were required to be at least 30 nucleotides in length, with at least 90% sequence identity; and the entire EST sequence alignment was required to extend over at least 90% of the length of the EST with at least 90% sequence identity.

In total, GENOA aligned about 85,900 human cDNAs and about 890,300 ESTs to the human genome. The relatively low fraction of aligned ESTs (about 18%), and average aligned length of about 550 bases (the average lengths were not significantly different between different tissues, see Additional data file 6), reflect the stringent alignment-quality criteria that were imposed so as to be as confident as possible in the inferred splicing patterns. The aligned sequences yielded about 17,800 gene regions with more than one transcript aligned that exhibited a multi-exon structure. Of these, about 60% exhibited evidence of alternative splicing of internal exons. Our analysis did not examine differences in 3'-terminal and 5'-terminal exons, inclusion of which is frequently dictated by alternative polyadenylation or alternative transcription start sites and therefore does not represent 'pure' AS [73,74]. The EST alignments were then used to categorize all internal exons as: constitutive exons; A3Es, A5Es, skipped exons, multiply alternatively spliced exons (for example, exons that exhibited both skipping and alternative 5' splice site usage), and exons that contained retained introns. An internal exon present in at least one transcript was identified as a skipped exon if it was precisely excluded in one or more other transcripts, such that the boundaries of both the 5' and 3' flanking exons were the same in the transcripts that included and skipped the exon (for example, exon E3 in Figure 1). Similarly, an internal exon present in at least one transcript was identified as an A3E or A5E if at least one other transcript contained an exon differing in length by the use of an alternative 3' splice site or 5' splice site. The 'core' of an A3E or A5E is defined as the exon portion that is common to all transcripts used to infer the AS event. The extension of an alternatively spliced exon is the exon portion added to the core region by the use of an alternative 3' splice site or 5' splice site) that is present in some, but not all transcripts used to infer the AS event. Pairs of inferred A3Es or A5Es differing by fewer than six nucleotides were excluded from further analysis, as in [8], because of the possibility that such small differences might sometimes result from EST sequencing or alignment errors. As the frequency of insertion-deletions errors greater than three bases using modern sequencing techniques is vanishingly small (P. Green, personal communication), a six-base cutoff should exclude the vast majority of such errors. Alternatively spliced exons/genes identified in specific tissues are available for download from the GENOA website [71].

### Quantifying splice junction differences between alternative mRNA isoforms

To quantify the difference in splicing patterns between mRNAs or ESTs derived from a gene locus, the splice junction difference ratio (SJD) was calculated. For any pair of mRNAs/ESTs that have been aligned to overlapping portions of a genomic locus, the SJD is defined as the fraction of the splice junctions that occur in overlapping portions of the two transcripts that differ in one or both splice sites. A sample calculation is given in Figure 4. The SJD measure was calculated by taking the ratio of the number of 'valid' splice junctions that differ between two sequences over the total number of splice junctions, when comparing a pair of ESTs across all splice junctions present in overlapping portions of the two transcripts. A splice junction was considered valid if: the 5' splice site and the 3' splice site satisfied either the GT..AG or the GC..AG dinucleotide sequences at exon-intron junctions; and if the splice junction was observed at least twice in different transcripts.

### Identification of candidate splicing regulatory motifs

Over-represented sequence motifs (*k*-mers) were identified by comparing the number of occurrences of *k*-mers (for *k *in the range of 4 to 6 bases) in a test set of alternative exons versus a control set. In this analysis, monomeric tandem repeats (for example, poly(A) sequences) were excluded. The enrichment score of candidate *k*-mers in the test set versus the control set was evaluated by computing χ^2 ^(chi-squared) values with a Yates correction term [75], using an approach similar in spirit to that described by Brudno *et al*. [59]. We randomly sampled 500 subsets of the same size as the test set from the control set. The enrichment scores for *k*-mers over-represented in the sampled subset versus the remainder of the control set were computed as above. The estimated *p*-value for observing the given enrichment score (χ^2^-value) associated with an over-represented sequence motif of length *k *was defined as the fraction of subsets that contained any *k*-mer with enrichment score (χ^2^-value) higher than the tested motif. Correcting for multiple testing is not required as the *p*-value was defined relative to the most enriched *k*-mer for each sampled set. For sets of skipped exons from human brain- and testis-derived EST sequences, the test sets comprised 1,265 and 517 exons skipped in brain- and testis-derived ESTs, respectively, and the control sets comprised 12,527 and 8,634 exons constitutively included in human brain- and testis-derived ESTs, respectively. Candidate sequence motifs in skipped exons from brain and testis-derived ESTs with associated *p*-values less than 0.002 were retained. For the set of A5E and A3E events from human liver-derived EST sequences, the test set comprised 44 A3Es and 45 A5Es, and the control set comprised 1,619 A3Es and 1,481 A5Es identified using ESTs from all tissues excluding liver. In this analysis, A3Es and A5Es with extension sequences of less than 25 bases were excluded and sequences longer than 150 bases were truncated to 150 bases, by retaining the exon sequence segment closest to the internal alternative splice junction. Over-represented sequence motifs in A3Es and A5Es from liver-derived EST sequences with associated *p*-values less than 0.01 were retained.

### Gene-expression analysis of *trans*-acting splicing factors

SR proteins, SR-related proteins, and hnRNPs were derived from published proteomic analyses of the spliceosome [46-48]. Expression values for these genes were obtained from the 'gene expression atlas' using the HG-U95A DNA microarray [43] and from a similar set of expression data using the HG-U133A DNA microarray [45]. Altogether, 20 splicing factors - ASF/SF2, SRm300, SC35, SRp40, SRp55, SRp30c, 9G8, SRp54, SFRS10, SRp20, hnRNPs A1, A2/B2, C, D, G, H1, K, L, M, and RALY - were studied in 26 different tissues present in both microarray experiments (Figure 5). The data from each gene chip - HG-U95A and HG-U133A - were analyzed separately. The average difference (AD) value of each probe was used as the indicator of expression level. In analyzing these microarray data, AD values smaller than 20 were standardized to 20, as in [43]. When two or more probes mapped to a single gene, the values from those probes were averaged. Pearson (product-moment) correlation coefficients between 20-dimensional vectors for all tissue pairs were calculated, using data from each of the two DNA microarray studies separately.

## Additional data files

Additional data files containing the following supplementary data, tables and figures are available with the online version of this paper and from the GENOA genome annotation website [71]. The lists of GenBank accession numbers of human cDNAs and ESTs that were mapped to the human genome by the GENOA pipeline, GENOA gene locus identifiers, and gene loci with spliced alignments for the 22 human autosomes and two sex chromosomes are provided at our website [76]. Sets of constitutive and alternative exons in genes expressed in the human brain, testis and liver, and control sets used are also provided [77]. Additional data file 1 lists the average and median number of ESTs per gene and tissue, and the total number of genes per tissue using different minimum numbers of ESTs. Additional data file 2 lists the average total number of AS genes and AS genes containing SEs, A3Es and A5Es using ESTs derived from normal, non-diseased tissues. Additional data file 3 lists the number of constitutively spliced and AS genes, and AS genes containing SEs, A3Es and A5Es. Additional data file 4 shows the average fractions of AS genes and average fractions of AS genes containing SEs, A3Es and A5Es using ESTs derived from normal, non-disease-derived tissues. Additional data file 5 lists categories of cDNA libraries and designated tissues derived from the MGC, IMAGE and CGAP. Additional data file 6 shows the average lengths of ESTs that aligned to gene loci expressed in different tissues. Additional data file 7 lists human splicing factors of SR, SR-related and hnRNP genes, corresponding Ensembl gene numbers and Affymetrix microarray probe identification numbers. Additional data file 8 shows the distribution of the average Pearson correlation coefficient values across different tissues for expression levels of random sets of genes obtained from the Affymetrix microarray data.

## Supplementary Material

Additional data file 1 The average and median number of ESTs per gene and tissue, and the total number of genes per tissue using different minimum numbers of ESTs Click here for additional data file

Additional data file 2 The average total number of AS genes and AS genes containing SEs, A3Es and A5Es using ESTs derived from normal, non-diseased tissues Click here for additional data file

Additional data file 3 The number of constitutively spliced and AS genes, and AS genes containing SEs, A3Es and A5Es Click here for additional data file

Additional data file 4 The average fractions of AS genes and average fractions of AS genes containing SEs, A3Es and A5Es using ESTs derived from normal, non-disease-derived tissues Click here for additional data file

Additional data file 5 Categories of cDNA libraries and designated tissues derived from the MGC, IMAGE and CGAP Click here for additional data file

Additional data file 6 The average lengths of ESTs that aligned to gene loci expressed in different tissues Click here for additional data file

Additional data file 7 Human splicing factors of SR, SR-related and hnRNP genes, corresponding Ensembl gene numbers and Affymetrix microarray probe identification numbers Click here for additional data file

Additional data file 8 The distribution of the average Pearson correlation coefficient values across different tissues for expression levels of random sets of genes obtained from the Affymetrix microarray datat Click here for additional data file

## Figures and Tables

**Table 1:** Human genes expressed in the liver with alternative 3' splice site exons (A3Es) or alternative 5' splice site exons (A5Es)

Splicing type	Ensembl gene ID	Gene name	Exon numbers	Fold-change above median expression, HG-U95A	Fold-change above median expression, MG-U74A
A5E;A3E	091513	Serotransferrin precursor, *TF*	8, 9; 4	100	100
A5E;A3E	115414	Fibronectin precursor, *FN1*	36; 31	10	-
A5E;A3E	117601	Antithrombin-III precursor, *SERPINC1*	5; 4	100	100
A5E;A3E	136872	Fructose-bisphosphate aldolase, *ALDOB*	3, 8; 4	100	10
A5E;A3E	140833	Haptoglobin-related protein precursor, *HPR*	3	100	10
A5E;A3E	151790	Tryptophan 2,3-dioxygenase, TDO2	3, 5; 4	10	100
A5E;A3E	171759	Phenylalanine-4-hydroxylase, *PAH*	6; 4,10	-	100
A5E	047457	Ceruloplasmin precursor, *CP*	14, 16	3	-
A5E	055957	Inter-alpha-trypsin inhibitor heavy chain H1 precursor, *ITIH1*	21	100	10
A5E	111275	Aldehyde dehydrogenase, *ALDH2*	12	3	3
A5E	132386	Pigment epithelium-derived factor precursor, *SERPINF1*	4	10	10
A5E	138356	Aldehyde oxidase, *AOX1*	27, 29	3	3
A5E	138413	Isocitrate dehydrogenase, *IDH1*	3	1	-
A5E	145692	Betaine-homocysteine *S*-methyltransferase, *BHMT*	4	10	100
A5E	160868	Cytochrome P450, *CYP3A4*	5	10	10
A5E	171766	Glycine amidinotransferase, *GATM*	8	3	3
A3E	080618	Carboxypeptidase, *CBP2*	10	-	-
A3E	080824	Heat shock protein HSP 90-alpha, *HSPCA*	8	-	-
A3E	096087	Glutathione *S*-transferase, *GSTA2*	4, 6	10	10
A3E	106927	Protein precursor, *AMBP*	5, 9	100	100
A3E	110958	Telomerase-binding protein P23, *TEBP*	5	<1	1
A3E	134240	Hydroxymethylglutaryl-CoA synthase, *HMGCS2*	8	10	-
A3E	138115	Cytochrome P450, *CYP2C8*	4	100	10
A3E	145192	Alpha-2-HS-glycoprotein precursor, *AHSG*	6	100	100
A3E	163631	Serum albumin precursor, *ALB*	9	100	100
A3E	171557	Fibrinogen gamma chain precursor, *FGG*	4	100	100
A3E	174156	Glutathione S-transferase, *GSTA3*	4, 6	10	10

Examples of human AS genes found to exhibit A3E and/or A5E splicing with both isoforms detected in liver-derived ESTs. AS types are listed in the first column, followed by the last six digits of the Ensembl gene number, the gene name and alternative exon numbers. The last two columns list expression levels in human liver and mouse liver tissues, respectively, expressed in terms of the fold-change relative to the median expression level in other tissues (from the DNA microarray data of [43] and [45], respectively).

**Table 2:** Sequence motifs enriched in skipped exons (SEs) and alternative 5' splice site exons (A5Es)

AS type /tissue (motif name)	Oligonucleotides	Occurrences	Consensus (% of exons containing)
			
SE/brain (BR1)	**CUCC**UG	169	CUCC (45.3)
	**CUCC**U	323	
	**CUCC**C	264	
	**CUCC**	945	
	C**CUCC**C	137	CCUC (41.0)
	**CCUC**C	363	
	**CCUC**	1021	
	**GCCUC**C	136	
	**GCCUC**	375	
	**GCCUC**A	122	
	G**GCCUC**	118	
	U**GCCUC**	108	
			
SE/brain (BR2)	**GGGU**U	97	GGGU (25.6)
	**GGGU**	411	
	A**GGGU**	116	
			
SE/brain (BR3)	**UGGG**A	324	UGGG (47.2)
	**UGGG**	948	
	C**UGGG**	426	
	CC**UGGG**	171	
			
SE/brain (BR4)	**GGGA**UU	58	GGGA (45.5)
	**GGGA**U	176	
	**GGGA**	840	
			
SE/brain (BR5)	**CUCA**	925	CUCA (46.5)
	**CUCA**C	206	
	GC**CUCA**	122	
	GG**CUCA**	102	
	G**CUCAC**	79	
	**CUCA**GC	126	
			
SE/brain (BR6)	**UAGC**	269	UAGC (18.0)
	**UAGC**U	106	
	G**UAGC**	96	
	G**UAGC**U	51	
	AG**UAGC**	47	
	**UAGC**UG	54	
			
SE/brain (BR7)	**UAGG**	186	UAGG (13.8)
	U**UAGG**	63	
	U**UAGG**G	24	
			
SE/testis (TE1)	**UAGG**	99	UAGG (16.6)
	U**UAGG**	33	
			
Core A5E/liver (LI1)	** AAAC**	42	AAAC (53.3)
	A**AAAC**	18	
			
Core A5E/liver (LI2)	**UAAA**	29	UAAA (40.0)
	**UAAA**CC	5	

Sequence motifs of length four to six bases that are significantly over-represented (*p *< 0.002) in SEs relative to constitutively spliced exons from brain- or testis-derived ESTs are shown, followed by the number of occurrences in SEs in these tissues. Sequence motifs are grouped/aligned by similarity, and shared tetramers are shown in bold and listed in the last column, followed by the fraction of SEs that contain the given tetramer. Sequence motifs significantly over-represented (*p *< 0.01) in the core of A5Es from human liver-derived ESTs are shown at the bottom, followed by the number of A5E occurrences and the fraction of A5Es that contain the given tetramer. Statistical significance was evaluated as described in Materials and methods.

## References

[B1] Black DL (2003). Mechanisms of alternative pre-messenger RNA splicing.. Annu Rev Biochem.

[B2] Cartegni L, Chew SL, Krainer AR (2002). Listening to silence and understanding nonsense: exonic mutations that affect splicing.. Nat Rev Genet.

[B3] Graveley BR (2001). Alternative splicing: increasing diversity in the proteomic world.. Trends Genet.

[B4] Lopez AJ (1998). Alternative splicing of pre-mRNA: developmental consequences and mechanisms of regulation.. Annu Rev Genet.

[B5] Grabowski PJ (2000). Genetic evidence for a Nova regulator of alternative splicing in the brain.. Neuron.

[B6] Zavolan M, Kondo S, Schonbach C, Adachi J, Hume DA, Hayashizaki Y, Gaasterland T, RIKEN GER Group, GSL members (2003). Impact of alternative initiation, splicing, and termination on the diversity of the mRNA transcripts encoded by the mouse transcriptome.. Genome Res.

[B7] Mironov AA, Fickett JW, Gelfand MS (1999). Frequent alternative splicing of human genes.. Genome Res.

[B8] Modrek B, Resch A, Grasso C, Lee C (2001). Genome-wide detection of alternative splicing in expressed sequences of human genes.. Nucleic Acids Res.

[B9] Modrek B, Lee C (2002). A genomic view of alternative splicing.. Nat Genet.

[B10] Kan Z, States D, Gish W (2002). Selecting for functional alternative splices in ESTs.. Genome Res.

[B11] Brett D, Hanke J, Lehmann G, Haase S, Delbruck S, Krueger S, Reich J, Bork P (2000). EST comparison indicates 38% of human mRNAs contain possible alternative splice forms.. FEBS Lett.

[B12] Faustino NA, Cooper TA (2003). Pre-mRNA splicing and human disease.. Genes Dev.

[B13] Modafferi EF, Black DL (1997). A complex intronic splicing enhancer from the c-src pre-mRNA activates inclusion of a heterologous exon.. Mol Cell Biol.

[B14] Naor D, Nedvetzki S, Golan I, Melnik L, Faitelson Y (2002). CD44 in cancer.. Crit Rev Clin Lab Sci.

[B15] MacDougall C, Harbison D, Bownes M (1995). The developmental consequences of alternate splicing in sex determination and differentiation in *Drosophila*.. Dev Biol.

[B16] Ladd AN, Charlet N, Cooper TA (2001). The CELF family of RNA binding proteins is implicated in cell-specific and developmentally regulated alternative splicing.. Mol Cell Biol.

[B17] Jensen KB, Dredge BK, Stefani G, Zhong R, Buckanovich RJ, Okano HJ, Yang YY, Darnell RB (2000). Nova-1 regulates neuron-specific alternative splicing and is essential for neuronal viability.. Neuron.

[B18] Lim LP, Burge CB (2001). A computational analysis of sequence features involved in recognition of short introns.. Proc Natl Acad Sci USA.

[B19] Fairbrother WG, Yeh RF, Sharp PA, Burge CB (2002). Predictive identification of exonic splicing enhancers in human genes.. Science.

[B20] Liu HX, Zhang M, Krainer AR (1998). Identification of functional exonic splicing enhancer motifs recognized by individual SR proteins.. Genes Dev.

[B21] Schaal TD, Maniatis T (1999). Selection and characterization of pre-mRNA splicing enhancers: identification of novel SR protein-specific enhancer sequences.. Mol Cell Biol.

[B22] Tian H, Kole R (2001). Strong RNA splicing enhancers identified by a modified method of cycled selection interact with SR protein.. J Biol Chem.

[B23] Zhu J, Shendure J, Mitra RD, Church GM (2003). Single molecule profiling of alternative pre-mRNA splicing.. Science.

[B24] Johnson JM, Castle J, Garrett-Engele P, Kan Z, Loerch PM, Armour CD, Santos R, Schadt EE, Stoughton R, Shoemaker DD (2003). Genome-wide survey of human alternative pre-mRNA splicing with exon junction microarrays.. Science.

[B25] Hu GK, Madore SJ, Moldover B, Jatkoe T, Balaban D, Thomas J, Wang Y (2001). Predicting splice variant from DNA chip expression data.. Genome Res.

[B26] Clark TA, Sugnet CW, Ares M (2002). Genome-wide analysis of mRNA processing in yeast using splicing-specific microarrays.. Science.

[B27] Ule J, Jensen KB, Ruggiu M, Mele A, Ule A, Darnell RB (2003). CLIP identifies Nova-regulated RNA networks in the brain.. Science.

[B28] Clark F, Thanaraj TA (2002). Categorization and characterization of transcript-confirmed constitutively and alternatively spliced introns and exons from human.. Hum Mol Genet.

[B29] Stamm S, Zhu J, Nakai K, Stoilov P, Stoss O, Zhang MQ (2000). An alternative-exon database and its statistical analysis.. DNA Cell Biol.

[B30] Xu Q, Modrek B, Lee C (2002). Genome-wide detection of tissue-specific alternative splicing in the human transcriptome.. Nucleic Acids Res.

[B31] Brett D, Pospisil H, Valcarcel J, Reich J, Bork P (2002). Alternative splicing and genome complexity.. Nat Genet.

[B32] Lee CJ, Irizarry K (2003). Alternative splicing in the nervous system: an emerging source of diversity and regulation.. Biol Psychiatry.

[B33] Wang Z, Lo HS, Yang H, Gere S, Hu Y, Buetow KH, Lee MP (2003). Computational analysis and experimental validation of tumor-associated alternative RNA splicing in human cancer.. Cancer Res.

[B34] Hui L, Zhang X, Wu X, Lin Z, Wang Q, Li Y, Hu G (2004). Identification of alternatively spliced mRNA variants related to cancers by genome-wide ESTs alignment.. Oncogene.

[B35] Kirkpatrick LL, McIlwain KA, Nelson DL (1999). Alternative splicing in the murine and human FXR1 genes.. Genomics.

[B36] Kirkpatrick LL, McIlwain KA, Nelson DL (2001). Comparative genomic sequence analysis of the FXR gene family: FMR1, FXR1, and FXR2.. Genomics.

[B37] Sugnet CW, Kent WJ, Ares M, Haussler D, Altman RB, Dunker AK, Hunter L, Jung TA, Klein TE (2004). Transcriptome and genome conservation of alternative splicing events in humans and mice.. In Biocomputing 2004: Pac Symp Biocomput.

[B38] Sorek R, Ast G (2003). Intronic sequences flanking alternatively spliced exons are conserved between human and mouse.. Genome Res.

[B39] Kaufmann D, Kenner O, Nurnberg P, Vogel W, Bartelt B (2004). In NF1, CFTR, PER3, CARS and SYT7, alternatively included exons show higher conservation of surrounding intron sequences than constitutive exons.. Eur J Hum Genet.

[B40] Megy K, Audic S, Claverie JM (2003). Positional clustering of differentially expressed genes on human chromosomes 20, 21 and 22.. Genome Biol.

[B41] Hillman RT, Green RE, Brenner SE (2004). An unappreciated role for RNA surveillance.. Genome Biol.

[B42] Green RE, Lewis BP, Hillman RT, Blanchette M, Lareau LF, Garnett AT, Rio DC, Brenner SE (2003). Widespread predicted nonsense-mediated mRNA decay of alternatively-spliced transcripts of human normal and disease genes.. Bioinformatics.

[B43] Su AI, Cooke MP, Ching KA, Hakak Y, Walker JR, Wiltshire T, Orth AP, Vega RG, Sapinoso LM, Moqrich A (2002). Large-scale analysis of the human and mouse transcriptomes.. Proc Natl Acad Sci USA.

[B44] Gene Expression Atlas. http://expression.gnf.org.

[B45] Su AI, Wiltshire T, Batalov S, Lapp H, Ching KA, Block D, Zhang J, Soden R, Hayakawa M, Kreiman G (2004). A gene atlas of the mouse and human protein-encoding transcriptomes.. Proc Natl Acad Sci USA.

[B46] Zhou Z, Licklider LJ, Gygi SP, Reed R (2002). Comprehensive proteomic analysis of the human spliceosome.. Nature.

[B47] Jurica MS, Moore MJ (2003). Pre-mRNA splicing: awash in a sea of proteins.. Mol Cell.

[B48] Rappsilber J, Ryder U, Lamond AI, Mann M (2002). Large-scale proteomic analysis of the human spliceosome.. Genome Res.

[B49] Hanamura A, Caceres JF, Mayeda A, Franza BR, Krainer AR (1998). Regulated tissue-specific expression of antagonistic pre-mRNA splicing factors.. RNA.

[B50] Bai Y, Lee D, Yu T, Chasin LA (1999). Control of 3' splice site choice *in vivo* by ASF/SF2 and hnRNP A1.. Nucleic Acids Res.

[B51] Eperon IC, Makarova OV, Mayeda A, Munroe SH, Caceres JF, Hayward DG, Krainer AR (2000). Selection of alternative 5' splice sites: role of U1 snRNP and models for the antagonistic effects of SF2/ASF and hnRNP A1.. Mol Cell Biol.

[B52] Kamma H, Portman DS, Dreyfuss G (1995). Cell type-specific expression of hnRNP proteins.. Exp Cell Res.

[B53] Caputi M, Mayeda A, Krainer AR, Zahler AM (1999). hnRNP A/B proteins are required for inhibition of HIV-1 pre-mRNA splicing.. EMBO J.

[B54] Caputi M, Zahler AM (2002). SR proteins and hnRNP H regulate the splicing of the HIV-1 tev-specific exon 6D.. EMBO J.

[B55] Anderson L, Seilhamer J (1997). A comparison of selected mRNA and protein abundances in human liver.. Electrophoresis.

[B56] Futcher B, Latter GI, Monardo P, McLaughlin CS, Garrels JI (1999). A sampling of the yeast proteome.. Mol Cell Biol.

[B57] Griffin TJ, Gygi SP, Ideker T, Rist B, Eng J, Hood L, Aebersold R (2002). Complementary profiling of gene expression at the transcriptome and proteome levels in *Saccharomyces cerevisiae*.. Mol Cell Proteomics.

[B58] Kawamoto S, Matsumoto Y, Mizuno K, Okubo K, Matsubara K (1996). Expression profiles of active genes in human and mouse livers.. Gene.

[B59] Brudno M, Gelfand MS, Spengler S, Zorn M, Dubchak I, Conboy JG (2001). Computational analysis of candidate intron regulatory elements for tissue-specific alternative pre-mRNA splicing.. Nucleic Acids Res.

[B60] Chou MY, Underwood JG, Nikolic J, Luu MH, Black DL (2000). Multisite RNA binding and release of polypyrimidine tract binding protein during the regulation of c-*src *neural-specific splicing.. Mol Cell.

[B61] Chan RC, Black DL (1997). The polypyrimidine tract binding protein binds upstream of neural cell-specific c-*src *exon N1 to repress the splicing of the intron downstream.. Mol Cell Biol.

[B62] Grabowski PJ (1998). Splicing regulation in neurons: tinkering with cell-specific control.. Cell.

[B63] Wollerton MC, Gooding C, Wagner EJ, Garcia-Blanco MA, Smith CW (2004). Autoregulation of polypyrimidine tract binding protein by alternative splicing leading to nonsense-mediated decay.. Mol Cell.

[B64] Burd CG, Dreyfuss G (1994). RNA binding specificity of hnRNP A1: significance of hnRNP A1 high-affinity binding sites in pre-mRNA splicing.. EMBO J.

[B65] Sirand-Pugnet P, Durosay P, Brody E, Marie J (1995). An intronic (A/U)GGG repeat enhances the splicing of an alternative intron of the chicken beta-tropomyosin pre-mRNA.. Nucleic Acids Res.

[B66] McCullough AJ, Berget SM (1997). G triplets located throughout a class of small vertebrate introns enforce intron borders and regulate splice site selection.. Mol Cell Biol.

[B67] UCSC Genome Browser. http://genome.ucsc.edu.

[B68] Mammalian Gene Collection (MGC) Initiative. http://mgc.nci.nih.gov.

[B69] The I.M.A.G.E. Consortium. http://image.llnl.gov/image.

[B70] The Cancer Gene Anatomy Project. http://cgap.nci.nih.gov.

[B71] GENOA file server. http://genes.mit.edu/genoa.

[B72] Florea L, Hartzell G, Zhang Z, Rubin GM, Miller W (1998). A computer program for aligning a cDNA sequence with a genomic DNA sequence.. Genome Res.

[B73] Berget SM (1995). Exon recognition in vertebrate splicing.. J Biol Chem.

[B74] Majewski J, Ott J (2002). Distribution and characterization of regulatory elements in the human genome.. Genome Res.

[B75] Glantz SA (1997). Primer of Biostatistics.

[B76] Burge lab: Additional data directory 1. http://genes.mit.edu/burgelab/Supplementary/yeo_holste04/Add_Datadir_1/.

[B77] Burge lab: Additional data directory 2. http://genes.mit.edu/burgelab/Supplementary/yeo_holste04/Add_Datadir_2/.

