# Supplementary material for: Variation in alternative splicing across human tissues
Source: Genome Biol. 2004 Sep 13;5(10):R74. doi: 10.1186/gb-2004-5-10-r74 (PMC545594; doi:10.1186/gb-2004-5-10-r74)
Supplement: Additional data file 1 — The average and median number of ESTs per gene and tissue, and the total number of genes per tissue using different minimum numbers of ESTs [file gb-2004-5-10-r74-s1.pdf]

Genes with >=1 EST per tissue			
Tissues	Mean	Median	Num. genes
kidney	3.69	2	6115
ovary	3.57	2	4771
breast	4.29	2	5877
uterus	4.89	2	6581
testis	4.29	2	7890
prostate	4.04	2	5466
colon	5.52	3	7343
eye_retina	5.13	2	6995
lung	6.72	3	7672
skin	5.92	3	6176
stomach	7.42	3	6181
brain	10.18	4	10437
placenta	6.82	3	6727
muscle	3.89	2	5243
pancreas	7.57	3	6658
liver	4.4	2	4326

Genes with >=30 ESTs per tissue			
Tissues	Mean	Median	Num. genes
kidney	42.5	36	20
ovary	38.82	35	33
breast	42.98	40	58
uterus	44.01	38	101
testis	45.95	39	64
prostate	46.82	36	57
colon	44.56	38	164
eye_retina	47.7	39	134
lung	51.16	42	276
skin	52.11	42	178
stomach	54.6	43	275
brain	57.73	46	797
placenta	70.31	41	212
muscle	74.29	51	56
pancreas	74.93	47	267
liver	81.44	50	72

Genes with >=20 EST per tissue			
Tissues	Mean	Median	Num. genes
kidney	28.16	25	82
ovary	29.01	25	87
breast	30.58	26	158
uterus	31.56	26	252
testis	32.49	27	161
prostate	33.15	28	140
colon	33.36	28	357
eye_retina	34.25	27	307
lung	36.8	29	582
skin	37.18	29	374
stomach	39.84	30	531
brain	42.7	31	1431
placenta	47.99	30	405
muscle	49.42	30	110
pancreas	51.02	31	502
liver	60.01	35	114

Genes with >=40 & <60 ESTs per tissue			
Tissues	Mean	Median	Num. genes
kidney	47.5	49	4
ovary	45.17	45	12
breast	48.13	48	23
uterus	47.83	47	36
testis	48.15	48	20
prostate	47.69	47	13
colon	47.52	46	54
eye_retina	47.21	46	43
lung	47.96	46	102
skin	48	48	62
stomach	47.87	48	100
brain	48.3	48	260
placenta	47.38	47	60
muscle	48.14	50	14
pancreas	48.31	47	91
liver	50.29	50	17

Table S1. Mean and median number of ESTs per gene, and the total number of genes inferred at different minimum number of ESTs required.
