# Supplementary material for: Variation in alternative splicing across human tissues
Source: Genome Biol. 2004 Sep 13;5(10):R74. doi: 10.1186/gb-2004-5-10-r74 (PMC545594; doi:10.1186/gb-2004-5-10-r74)
Supplement: Additional data file 2 — The average total number of AS genes and AS genes containing SEs, A3Es and A5Es using ESTs derived from normal, non-diseased tissues [file gb-2004-5-10-r74-s2.pdf]

Normal tissues	Average number of genes	Average number of AS genes	Average number of genes with skipped exons	Average number of genes with alternative 5'ss exons	Average number of genes with alternative 3'ss exons
kidney	75.9	11.9	8.1	2.1	2.0
pancreas	288.7	32.1	21.3	4.5	8.8
testis	189.7	44.7	34.1	6.8	8.0
eye-retina	267.0	34.9	19.5	8.7	9.0
stomach	734.6	90.8	62.0	12.6	24.2
brain	1635.8	423.3	272.9	102.6	108.7
placenta	409.4	59.1	36.8	14.3	14.4
breast	173.7	15.9	12.1	2.5	2.6
muscle	177.1	15.6	12.5	1.5	3.5
uterus	71.3	4.2	2.2	2.0	0.0
liver	91.8	26.3	5.6	13.3	13.6
lung	430.7	62.9	41.1	11.6	16.1

Table S2. The average total number of genes, AS genes, genes containing skipped, exons, genes containing alternative 5'ss or 3'ss exons.   Splicing patterns were inferred utilizing 10 ESTs from genes with at least 15 ESTs from a particular normal tissue cDNA library.
