# Supplementary material for: Variation in alternative splicing across human tissues
Source: Genome Biol. 2004 Sep 13;5(10):R74. doi: 10.1186/gb-2004-5-10-r74 (PMC545594; doi:10.1186/gb-2004-5-10-r74)
Supplement: Additional data file 3 — The number of constitutively spliced and AS genes, and AS genes containing SEs, A3Es and A5Es [file gb-2004-5-10-r74-s3.pdf]

AS event	Normal tissue	Disease-associated tissue	Normal and disease
	ASG (CSG)	ASG (CSG)	ASG (CSG)
SE	195 (1201)	275 (1115)	261 (1128)
A5E	134 (1261)	119 (1271)	147 (1242)
A3E	124 (1271)	107 (1266)	107 (1282)
ASG	382 (1013)	435 (947)	435 (954)

AS event	Normal tissue	Disease-associated tissue	Normal and disease
	ASG / (CSG+ASG)	ASG / (CSG+ASG)	ASG / (CSG+ASG)
SE	14%	20%	19%
A5E	10%	9%	11%
A3E	9%	9%	8%
ASG	27%	32%	31%

Table S3. The number of alternatively spliced genes (ASGs) and constitutively spliced genes (CSGs), genes containing skipped exons (SEs), alternative 5'ss (A5E), and alternative 3'ss exons. Splicing patterns were inferred by using 16 ESTs per gene from normal cDNA libraries, 16 ESTs per gene from diseased cDNA libraries, and 8 ESTs from normal and 8 ESTs from diseased cDNA libraries. ESTs from the 16 tissue libraries similar to that in Figure 1 were combined in this analysis. The fraction of genes containing SEs using ESTs from normal tissue libraries was significantly smaller (p <1E-5) by a chi-square test (with Yates correction), as compared to using ESTs from disease associated libraries.
