# Supplementary material for: Variation in alternative splicing across human tissues
Source: Genome Biol. 2004 Sep 13;5(10):R74. doi: 10.1186/gb-2004-5-10-r74 (PMC545594; doi:10.1186/gb-2004-5-10-r74)

Figure S1. Sampling 10 ESTs from gene regions with at least 15 ESTs aligned the region. ESTs are derived from strictly normal cDNA libraries.

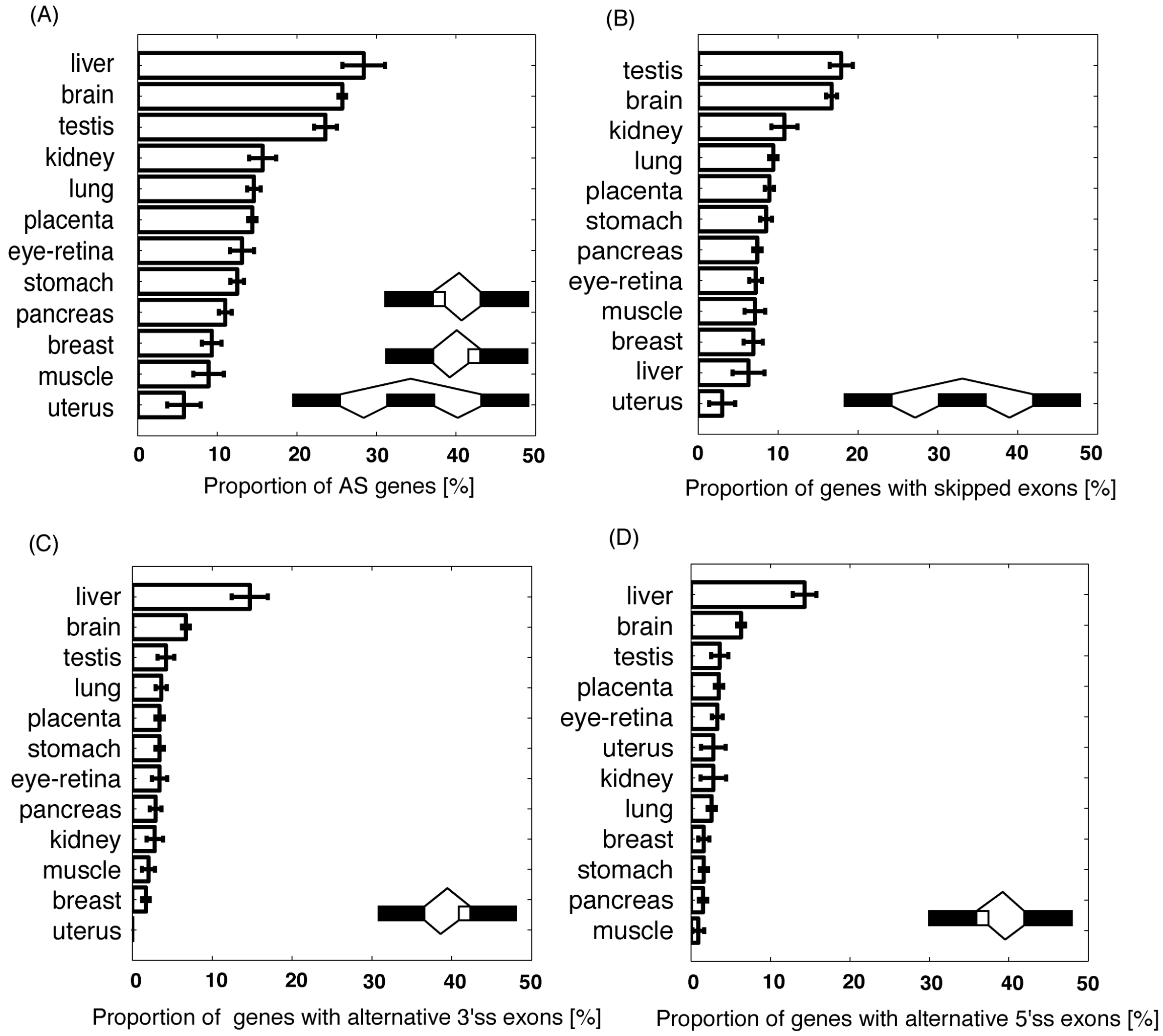

Supplement: Additional data file 4 — The average fractions of AS genes and average fractions of AS genes containing SEs, A3Es and A5Es using ESTs derived from normal, non-disease-derived tissues [file gb-2004-5-10-r74-s4.pdf]
