# Supplementary material for: Variation in alternative splicing across human tissues
Source: Genome Biol. 2004 Sep 13;5(10):R74. doi: 10.1186/gb-2004-5-10-r74 (PMC545594; doi:10.1186/gb-2004-5-10-r74)
Supplement: Additional data file 5 — Categories of cDNA libraries and designated tissues derived from the MGC, IMAGE and CGAP [file gb-2004-5-10-r74-s5.pdf]

Supplementary Material, Tab. S4

May, 2004

Categories of cDNA libraries and designated tissues, derived from:

- Mammalian Gene Collection (MGC)
- Integrated Molecular Analysis of Genomes and their Expression (IMAGE)
- Cancer Genome Anatomy Project (CGAP).

For each entry in the EST database (dbEST), we extract

- EST identification number "dbEST Id" (e.g., 90695)
- GenBank Accession number (e.g., T39891)
- EST sequence
- cDNA library name "Lib Name" (e.g., Stratagene liver (#937224)).

If the cDNA library has been taken from MGC, IMAGE or CGAP, the library name is matched to the original designated tissue. For instance, EST BE764871 has been derived from the cDNA library NT0086 and was designated as "brain" in CGAP. Cases with nonspecific tissue description (e.g., cDNA library HT29M6) where catagorized as "uncharacterized" or "pooled" tissue.

For a flat file containing EST tissue information, please send email to: holste@mit.edu.

cDNA Library	Designated Tissue for Library
Stratagene liver (#937224)	liver
Stratagene ovary (#937217)	ovary
Soares fetal liver spleen 1NFLS	pooled tissue
Soares infant brain 1NIB	brain
Human fetal brain (TFujiwara)	brain
Human aorta polyA+ (TFujiwara)	vascular
Human placenta polyA+ (TFujiwara)	placenta
Human fetal brain QBoqin	uncharacterized tissue
Human hepatoma RHWenger	uncharacterized tissue
Human fibroblast cDNA	uncharacterized tissue
Human pancreatic cancer cell line Patu 8988t	pancreas
Human frontal cortex, Stratagene	uncharacterized tissue
Human vulva squamous carcinoma A431	uncharacterized tissue
Human exon amplification Treacher Collins gene region	uncharacterized tissue
multi-tissue normalized short-fragment	uncharacterized tissue
normal female breast tissue	uncharacterized tissue
fetal brain cDNA	uncharacterized tissue
Human fetal brain QBoqin2	brain
Human fovea cDNA	retina
Chromosome 16p13.3 Exon	uncharacterized tissue
Human normal gingiva	uncharacterized tissue
Human pancreatic tumor	uncharacterized tissue
Human retina cDNA Tsp509I-cleaved sublibrary	retina
Human retina cDNA randomly primed sublibrary	retina
Fetal heart	heart
Human fetal brain, Stratagene	uncharacterized tissue
Human PGasparini	uncharacterized tissue
Stratagene fetal spleen (#937205)	spleen
Soares placenta Nb2HP	placenta
Soares breast 2NbHBst	mammary gland
Soares breast 3NbHBst	mammary gland
Soares adult brain N2b4HB55Y	brain
Soares adult brain N2b5HB55Y	brain
Soares retina N2b4HR	retina
Soares ovary tumor NbHOT	ovary
Weizmann Olfactory Epithelium	head and neck
Soares melanocyte 2NbHM	skin
Morton Fetal Cochlea	ear
Pancreatic Islet	pancreatic islet
CLONTECH cDNA library CCRF-CEM, cat# HL1063g	uncharacterized tissue

cDNA Library	Designated Tissue for Library
HT29M6	uncharacterized tissue
Human chromosome 17	uncharacterized tissue
BATM1	uncharacterized tissue
Human, Meese.E.	uncharacterized tissue
Adult heart, Clontech	heart
Outward Alu-primed hncDNA library	uncharacterized tissue
Inward Alu-primed hncDNA library	uncharacterized tissue
Human fetal lung	lung
Human adult lung 3' directed MboI cDNA	lung
HDMEC cDNA library	vascular
Subtracted human retina	uncharacterized tissue
Selected chromosome 21 cDNA library	uncharacterized tissue
22 week old human fetal liver cDNA library	liver
Subtractive cDNA library ocular ciliary body	eye
Cot1374Ft-4HB3MA	uncharacterized tissue
Human chromosome 8	uncharacterized tissue
Chromosome 21 exon	uncharacterized tissue
Chromosome 19p12-p13.1 exon	uncharacterized tissue
Human stem cell	uncharacterized tissue
Human fetal brain	uncharacterized tissue
human adult testis	testis
Clontech human fetal brain polyA+ mRNA (#6535)	brain
Clontech human placenta polyA+ mRNA (#6572)	placenta
Testis, Subtracted	uncharacterized tissue
HE6W	uncharacterized tissue
Human ocular ciliary body cDNA library 1	uncharacterized tissue
Human ocular ciliary body cDNA library 2	uncharacterized tissue
Chromosome 21, K.Gardiner	uncharacterized tissue
Chromosome 22 exon	uncharacterized tissue
21q Placenta, F.Tassone and K.Gardiner	uncharacterized tissue
Human chromosome 12p cDNAs	uncharacterized tissue
BATM2	uncharacterized tissue
Human thymus NSTH II	thymus
Human normalized K562-cDNA	uncharacterized tissue
Human HL60	uncharacterized tissue
HM1	muscle
Human cerebral cortex	cerebrum
Human	uncharacterized tissue
WATM1	adipose
Stratagene lung (#937210)	lung
Stratagene placenta (#937225)	placenta

cDNA Library	Designated Tissue for Library
STRATAGENE Human skeletal muscle cDNA library, cat.#936215	muscle
normalized infant brain cDNA	brain
Human Adipose tissue	adipose
Human Adrenal gland	endocrine
Human Bone	bone
Human Brain	brain
Human Breast	mammary gland
Human Colon	colon
Human Embryo	whole body
Human Endothelial cells	vascular
Human Epididymis	genitourinary
Human Esophagus	uncharacterized tissue
Human Eye	eye
Human Gall bladder	uncharacterized tissue
Human Greater omentum	uncharacterized tissue
Human Heart	heart
Human Kidney	kidney
Human Liver	liver
Human Lung	lung
Human Lymphoid tissue	uncharacterized tissue
Human Ovary	ovary
Human Pancreas	pancreas
Human Parathyroid gland	uncharacterized tissue
Human Placenta	placenta
Human Platelet	uncharacterized tissue
Human Prostate gland	prostate
Human Salivary gland	salivary gland
Human Skeletal muscle	uncharacterized tissue
Human Skin	skin
Human Small intestine	gastrointestinal tract
Human Spleen	spleen
Human Synovial membrane	synovium
Human Testis	testis
Human Thymus gland	uncharacterized tissue
Human Thyroid gland	thyroid
Human Uterus	uterus
Human White blood cells	whole blood
Infant Brain	uncharacterized tissue
Hippocampus	uncharacterized tissue
Human brain ARSanders	uncharacterized tissue

cDNA Library	Designated Tissue for Library
Human osteosarcoma EGracia	uncharacterized tissue
Human glioblastoma UFischer	uncharacterized tissue
Human lung adenocarcinoma A549	uncharacterized tissue
Human cord blood mononuclear cell mRNA	uncharacterized tissue
Human (NSHeiss)	uncharacterized tissue
RA-MO-III	uncharacterized tissue
Human (Fan,Wufang)	uncharacterized tissue
Human adult (K.Okubo)	uncharacterized tissue
Soares retina N2b5HR	retina
Human heart cDNA (YNakamura)	heart
Human peripheral blood (Steve Elledge)	uncharacterized tissue
HPLA CCLee	placenta
Human pancreatic cancer (CWallrapp)	uncharacterized tissue
Human fetal brain S.Meier-Ewert	brain
Human alveolar rhabdomyosarcoma	uncharacterized tissue
Stratagene colon (#937204)	colon
Stratagene corneal stroma (#937222)	eye
Human endothelial cell (Y.Mitsui)	vascular
Stratagene pancreas (#937208)	pancreas
Stratagene fibroblast (#937212)	skin
Testis 5	testis
Human brain cDNA	brain
Stratagene neuroepithelium (#937231)	cerebrum
Clontech adult lung cDNA library (HL1158a)	uncharacterized tissue
Human (P.Hui)	uncharacterized tissue
Human exocervical cells (CGLee)	uncharacterized tissue
Human placenta cDNA (TFujiwara)	placenta
Stratagene ovarian cancer (#937219)	ovary
Human 23132 gastric carcinoma cell line	stomach
Chromosome 7 Fetal Brain cDNA Library	brain
Chromosome 7 Placental cDNA Library	placenta
Chromosome 7 HeLa cDNA Library	uncharacterized tissue
Chromosome 7 Thymus cDNA Library	thymus
Stratagene colon HT29 (#937221)	colon
Stratagene endothelial cell 937223	vascular
Stratagene neuroepithelium NT2RAMI 937234	cerebrum
Stratagene hNT neuron (#937233)	peripheral nervous system
Stratagene HeLa cell s3 937216	cervix
Stratagene muscle 937209	muscle
Human thymus (V.L.Boyartchuk)	uncharacterized tissue

cDNA Library	Designated Tissue for Library
Human fetal brain (G.G.Consalez)	uncharacterized tissue
Stratagene NT2 neuronal precursor 937230	peripheral nervous system
Stratagene lung carcinoma 937218	lung
Stratagene fetal retina 937202	retina
Human newborn melanocytes (T.Vogt)	skin
Human neuroblastoma SK-ER3 cells (M.Garnier)	uncharacterized tissue
Human thymus 610t (M.D'Urso)	uncharacterized tissue
Human keratinocyte differential display (B.Lin)	uncharacterized tissue
Human infant brain (J.-F.Cheng)	uncharacterized tissue
Human Ulcerative Colitis Mucosa Express Library	uncharacterized tissue
Human GM-CSF-deprived TF-1 cell line (Liu,Hongtao)	uncharacterized tissue
Soares placenta Nb2HP-B	uncharacterized tissue
Human microdissection mediated cDNA capture clones	uncharacterized tissue
Human cell line TF-1 (D.L.Ma)	uncharacterized tissue
KG1-a Lambda Zap Express cDNA library	uncharacterized tissue
Human RT-PCR (S.Lefebvre)	uncharacterized tissue
Human breast cancer cell line Bcap 37	uncharacterized tissue
Human HeLa cells (M.Lovett)	uncharacterized tissue
Human fetal brain (M.Lovett)	uncharacterized tissue
Human (M.Lovett)	uncharacterized tissue
Human fetal heart, Lambda ZAP Express	heart
Stratagene schizo brain S11	cerebrum
Human retina (D.Swanson)	uncharacterized tissue
Human LAK cell (Y.Abe)	uncharacterized tissue
Human placental cDNA (Cheng Chi Lee)	uncharacterized tissue
Human YAC-derived (I.H.Still)	uncharacterized tissue
Fetal brain	uncharacterized tissue
Heart III	uncharacterized tissue
Lung	lung
Bone VII	bone
Colon I	colon
Liver I	liver
Brain IX	uncharacterized tissue
Cornea I	uncharacterized tissue
Thymus I	thymus
Cornea II	uncharacterized tissue
Trachea tumor	uncharacterized tissue
Fetal lung I	lung
Fibrosarcoma	uncharacterized tissue
Lung tumor I	uncharacterized tissue

cDNA Library	Designated Tissue for Library
Platelet	uncharacterized tissue
Skin tumor I	skin
Thymus III	thymus
Frontal lobe	uncharacterized tissue
Lung tumor II	lung
Retina I	uncharacterized tissue
Skin tumor II	uncharacterized tissue
Supt cells	uncharacterized tissue
Fetal heart I	uncharacterized tissue
Fetal lung III	lung
Fetal spleen	spleen
Testis I	testis
Uterus	uterus
Fetal brain VI	uncharacterized tissue
Fetal heart II	heart
Fetal liver IV	liver
MCF7 cell line	uncharacterized tissue
Ovary II	ovary
Fetal brain III	brain
Fetal duramater	uncharacterized tissue
Fetal kidney I	kidney
Fetal liver III	liver
Infant brain	brain
LNCAP cells I	prostate
Retina II	retina
Thymus tumor I	thymus
Thyroid	thyroid
Uterus tumor I	uterus
CAMA1Ee cell line I	uncharacterized tissue
Fetal kidney II	kidney
Leiomyloid tumor	uncharacterized tissue
Skeletal muscle	uncharacterized tissue
Thymus tumor II	thymus
Uterus tumor II	uncharacterized tissue
CAMA1Ee cell line II	uncharacterized tissue
Embryo, 7 week	whole body
Embryo, 9 week	whole body
Fetal bone	bone
Lymph node I	lymph node
Macrophage I	uncharacterized tissue

cDNA Library	Designated Tissue for Library
T-cell lymphoma	uncharacterized tissue
Testis tumor	testis
Thymus tumor III	uncharacterized tissue
Epithilioid sarcoma	skin
Jurkat T-cells I	uncharacterized tissue
Jurkat T-cells V	uncharacterized tissue
Liver III	liver
Lymph node II	uncharacterized tissue
Macrophage II	uncharacterized tissue
Epididymus	genitourinary
Esophagus tumor	esophagus
Heart I	heart
Jurkat T-cells II	uncharacterized tissue
Jurkat T-cells VI	uncharacterized tissue
Namalwa B cells I	uncharacterized tissue
Pancreas tumor I	pancreas
Embryo, 6 week II	whole body
Greater omentum IV	soft tissue
Jurkat T-cells III	uncharacterized tissue
Liver II	uncharacterized tissue
Namalwa B cells II	uncharacterized tissue
Ovarian cancer	uncharacterized tissue
Pancreas tumor II	uncharacterized tissue
Pineal gland I	uncharacterized tissue
Embryo, 12 week I	whole body
Fetal muscle	uncharacterized tissue
Ovary I	ovary
Pancreas tumor III	pancreas
Pineal gland II	pineal gland
Spleen I	spleen
Bone marrow	bone marrow
Cerebellum I	uncharacterized tissue
Embryo, 12 week II	whole body
Placenta II	placenta
Placenta I	placenta
Thymus II	thymus
Cerebellum II	cerebellum
Colon adenocarcinoma IV	colon
Greater omentum I	uncharacterized tissue
Greater omentum tumor	uncharacterized tissue

cDNA Library	Designated Tissue for Library
Kidney I	kidney
Salivary gland	salivary gland
Fetal lung II	lung
Greater omentum II	uncharacterized tissue
Kidney IX	uncharacterized tissue
Embryo, 6 week I	whole body
Greater omentum III	soft tissue
Pancreas I	pancreas
Pituitary gland	pituitary gland
Activated T-cells I	uncharacterized tissue
Activated T-cells V	uncharacterized tissue
Activated T-cells X	uncharacterized tissue
Brain IV	brain
Pancreas II	pancreas
Activated T-cells II	uncharacterized tissue
Activated T-cells IV	uncharacterized tissue
Activated T-cells IX	uncharacterized tissue
Activated T-cells VI	uncharacterized tissue
Activated T-cells XI	uncharacterized tissue
Activated T-cells XX	uncharacterized tissue
Adrenal gland tumor	endocrine
Adrenal gland	uncharacterized tissue
Esophagus	esophagus
Fetal brain I	brain
Fetal liver I	liver
Lymph node, subtracted	uncharacterized tissue
Paratid gland tumor	uncharacterized tissue
Small intestine I	gastrointestinal tract
Activated T-cells III	uncharacterized tissue
Activated T-cells VII	uncharacterized tissue
Activated T-cells XII	uncharacterized tissue
Fetal liver II	liver
Monocytes, stimulated II	uncharacterized tissue
Small intestine II	gastrointestinal tract
Activated T-cells VIII	uncharacterized tissue
Endometrial tumor	uterus
Fetal brain II	uncharacterized tissue
Red blood cell	bone marrow
Heart, subtracted (total cDNA)	uncharacterized tissue
Fetal kidney III	kidney

cDNA Library	Designated Tissue for Library
Ovary, subtracted	uncharacterized tissue
Prostate gland I	prostate
Rhabdomyosarcoma	muscle
Spleen, subtracted	uncharacterized tissue
Corpus callosum I	uncharacterized tissue
Frontal cortex II	uncharacterized tissue
Prostate gland V	uncharacterized tissue
Right hemisphere	uncharacterized tissue
Corpus callosum II	brain
Embryo, 8 week I	whole body
Fetal brain VII	uncharacterized tissue
Fetal skin	skin
Gall bladder I	uncharacterized tissue
Fetal heart, subtracted (total cDNA)	uncharacterized tissue
Gall bladder II	uncharacterized tissue
Hippocampus I	cerebrum
Adipose tissue, brown	adipose
CCRF-CEM cells, cyclohexamide treated I	uncharacterized tissue
Fetal brain, subtracted	uncharacterized tissue
HSC172 cells I	lung
Hippocampus II	cerebrum
Liver, subtracted (abundant clones) I	liver
Parathyroid gland tumor I	parathyroid
Raji cells, cyclohexamide treated I	uncharacterized tissue
HSC172 cells II	lung
Liver, subtracted (abundant clones) II	liver
Parathyroid gland tumor II	uncharacterized tissue
Thymus tumor, subtracted (total cDNA)	thymus
Adipose tissue, white I	adipose
Aorta endothelial cells	vascular
Adipose tissue, white II	adipose
Liver, hepatocellular carcinoma	liver
Macrophage, subtracted (total cDNA)	uncharacterized tissue
Synovial membrane	synovium
Left hemisphere	uncharacterized tissue
Synovial sarcoma	uncharacterized tissue
Infant adrenal gland I	endocrine
Embryo, 6 week, subtracted (total cDNA) I	whole body
Embryo, 7 week, subtracted (total cDNA) I	whole body
Infant adrenal gland II	endocrine

cDNA Library	Designated Tissue for Library
Placenta, subtracted (total cDNA)	placenta
Embryo, 6 week, subtracted (total cDNA) II	whole body
Embryo, 7 week, subtracted (total cDNA) II	whole body
Pancreas tumor, subtracted (abundant clones)	pancreas
Aorta endothelial cells, TNF alpha-treated	vascular
Right hemisphere, subtracted (mitochondria)	uncharacterized tissue
Umbilical vein endothelial cells I	vascular
Umbilical vein endothelial cells II	vascular
Pituitary gland, subtracted (prolactin/growth hormone) II	pituitary gland
Infant adrenal gland, subtracted (total cDNA) I	endocrine
Colon carcinoma (Caco-2) cell line I	colon
HCC cell line (matastasis to liver in mouse)	colon
Colon carcinoma (Caco-2) cell line II	colon
Retinal pigment epithelium 0041 cell line	uncharacterized tissue
Colon carcinoma (HCC) cell line	colon
HCC cell line (matastasis to liver in mouse) II	colon
Colon carcinoma (HCC) cell line II	colon
Human Breast Cancer	uncharacterized tissue
Fetal heart, Lambda ZAP Express	heart
Adult heart, Lambda gt11	heart
RA-MO-I	uncharacterized tissue
HM3	muscle
Human fetal brain QBoqin3	uncharacterized tissue
PN001-Normal Human Prostate	prostate
Human fetal brain QBoqin4	uncharacterized tissue
Gessler Wilms tumor	soft tissue
Human MCF7 cDNA subtracted with MDA-MB-231 cDNA	uncharacterized tissue
Human Bone Marrow Stromal Fibroblast	soft tissue
Human fetal liver (S.Xue)	uncharacterized tissue
Human RAP-PCR products	uncharacterized tissue
Human bone marrow stromal cells	bone marrow
Human erythroleukemia RAP-PCR products	uncharacterized tissue
Human promyelocytic HL60 cell line (S.Herblot)	uncharacterized tissue
Human immortalized fibroblasts (H.L.Ozer)	uncharacterized tissue
Human lung carcinoma (E.P.Diamandis)	uncharacterized tissue
Human chromosome 14	uncharacterized tissue
Human fetal brain chromosome 21	uncharacterized tissue
Human chromosome 6	uncharacterized tissue
GM10791 library (Eric D.Green)	uncharacterized tissue
Total human library (David Schlessinger)	uncharacterized tissue

cDNA Library	Designated Tissue for Library
4AF1/106/KO15 library (Lap-Chee Tsui)	uncharacterized tissue
Human chromosome 11q23 mRNA (M.Katoh)	uncharacterized tissue
DKFZphamy1	brain
DKFZphsnu1	brain
DKFZphthml	uncharacterized tissue
Human embryo lung HEL cell (Pingkun Zhou)	uncharacterized tissue
Regional genomic DNA specific cDNA library	uncharacterized tissue
subtracted 3' EST library	uncharacterized tissue
Human Glialblastoma Cell	brain
Human Brain, Clontech	uncharacterized tissue
Human brain frontal cortex	uncharacterized tissue
Human HaCaT keratinocyte cDNA	uncharacterized tissue
Human Fetal Brain+Liver+Heart	uncharacterized tissue
Human Lung cDNA Library	uncharacterized tissue
Barstead pancreas HPLRB1	pancreas
Barstead spleen HPLRB2	spleen
Human retina cell line ARPE-19	uncharacterized tissue
Normal Human Trabecular Bone Cells	bone
Clontech catalog #CLHL1076b	uncharacterized tissue
Stratagene catalog #936206	uncharacterized tissue
Human (J.Swensen)	uncharacterized tissue
Human pancreatic islet cell	uncharacterized tissue
EST from 8p21.3-p22	uncharacterized tissue
Human lung adenocarcinoma (M.Wu)	uncharacterized tissue
Human brain (K.Lueders)	uncharacterized tissue
Clontech #HL1181a	uncharacterized tissue
Clontech Hl1149x	uncharacterized tissue
Human HeLa (Y.Wang)	uncharacterized tissue
Human chromosome 13q14 cDNA	uncharacterized tissue
Human fetal trachea fibroblast	uncharacterized tissue
Human brain cDNA (Life Technologies)	uncharacterized tissue
Human cell line PCI-O6A	uncharacterized tissue
Human cell line PCI-O6B	uncharacterized tissue
Human mRNA (Tripodis and Ragoussis)	uncharacterized tissue
Human fibrosarcoma cell line HT1080	uncharacterized tissue
Human fibrosarcoma cell line HT1080-6TGc5	uncharacterized tissue
Human cell line SK-N-MC	uncharacterized tissue
Chromosome 5q33 transcripts	uncharacterized tissue
Human activated dendritic cell mRNA	uncharacterized tissue
Human cDNA (Chandrasekharappa,S.C.)	uncharacterized tissue

cDNA Library	Designated Tissue for Library
Human kidney (Bi,A.)	uncharacterized tissue
Human testis (Bi,A.)	uncharacterized tissue
Human bone marrow (Bi,A.)	uncharacterized tissue
Human skeletal muscle (Bi,A.)	uncharacterized tissue
Human fetal brain (R.L.Margolis)	uncharacterized tissue
Human cerebral cortex (R.L.Margolis)	uncharacterized tissue
Human cell line AGZY-83a	uncharacterized tissue
Human chromosome 5q31-q33 mRNA	uncharacterized tissue
Human lymphocytes cDNA (R.Meneveri)	uncharacterized tissue
Human small intestine cDNA (R.Meneveri)	uncharacterized tissue
Human ovary (H.Wang)	uncharacterized tissue
Human leukemia matchmaker, Clontech catalog #HL4015AB	uncharacterized tissue
Human lung cancer cell line A549.A549	uncharacterized tissue
Human salivary gland cell line HSG	salivary gland
Human Jurkat cell line mRNA (Thiele,K.)	uncharacterized tissue
Human testis (C.De Smet)	uncharacterized tissue
Human mRNA (H.M.Robertson)	uncharacterized tissue
Human mRNA (G.La Mantia)	uncharacterized tissue
Human T-cell cDNA library (M.G.Smirnova)	uncharacterized tissue
Human hippocampus, Stratagene catalog #936205	uncharacterized tissue
Human cDNA from Wiskott-Aldrich syndrome gene region	uncharacterized tissue
Human chromosome 21q22 mRNA	uncharacterized tissue
Human skin lesion from patient with systemic sclerosis	uncharacterized tissue
Human TNF-treated BG9 fibroblasts	uncharacterized tissue
Human primary melanocytes mRNA (I.M.Eisenbarth)	skin
Human leukocyte (M.L.Markelov)	uncharacterized tissue
Human female brain (Life Technologies)	uncharacterized tissue
Human fetal brain cDNA (T.M.Gress)	uncharacterized tissue
ICRFHFB	uncharacterized tissue
Human fetal brain (S.E.Mole)	uncharacterized tissue
Human colorectal cancer (Genome Res.1995)	uncharacterized tissue
BL29 Burkitt's lymphoma (K.M.Timms)	uncharacterized tissue
Human adult thymus NSTH II	uncharacterized tissue
AP20 melanoma mRNA	uncharacterized tissue
Human lymphocyte mRNA (Zhu,L.-P.)	uncharacterized tissue
human glioblastoma library	uncharacterized tissue
Human neuroepithelium (N.Jiang)	uncharacterized tissue
HL60 cDNA library	uncharacterized tissue
Human gastric cancer SGC-7901 cell line	stomach
Johnston frontal cortex	cerebrum

cDNA Library	Designated Tissue for Library
monocyte-derived dendritic cell library	uncharacterized tissue
Barstead aorta HPLRB3	vascular
Barstead prostate BPH HPLRB4	uncharacterized tissue
Human fetal liver (J.Zhang)	uncharacterized tissue
Human medullary thyroid carcinoma	uncharacterized tissue
Human gastric carcinoma mRNA	stomach
Human adult aorta	uncharacterized tissue
Human fetal brain (Zakharev,V.M.and Belyavsky,A.V.)	uncharacterized tissue
Human intestine from Crohn’s disease patients	uncharacterized tissue
Human fetal liver cDNA library	liver
Yunnan Tin miner lung cancer tissue mRNA	uncharacterized tissue
HeLa cDNA (T.Noma)	uncharacterized tissue
Human brain mRNA (U.K.Rout)	uncharacterized tissue
human embryo cDNA library	whole body
human kidney (N.Fuse)	uncharacterized tissue
Human trapped exon (S.E.Antonarakis)	uncharacterized tissue
Schiller astrocytoma	brain
Schiller glioblastoma multiforme	uncharacterized tissue
Schiller meningioma	brain
Schiller oligodendroglioma	brain
Human oral keratinocytes	uncharacterized tissue
Subtracted cDNA library of activated B lymphocyte	uncharacterized tissue
cDNA library of activated B cell line 3D5	uncharacterized tissue
Human placenta RT-PCR amplified cDNA	uncharacterized tissue
Human OCI LY8-C3P	uncharacterized tissue
Human C3-A11N	uncharacterized tissue
Human liver EST (Y.L.Yu)	uncharacterized tissue
Human cell line A431 subclone	uncharacterized tissue
Stanley Frontal NB pool 2	cerebrum
Stanley Frontal NS pool 2	cerebrum
Stanley Frontal SB pool 1	cerebrum
Stanley Frontal SN individual	cerebrum
Stanley Frontal SN pool 1	cerebrum
Stanley Frontal SN pool 2	cerebrum
Stanley Hippocampus NB pool 1	uncharacterized tissue
Stanley Hippocampus SB pool 1	cerebrum
Stanley Hippocampus SN pool 1	cerebrum
Human E8CASS	uncharacterized tissue
Retinoid treated HeLa cells	uncharacterized tissue
Barstead prostate BPH HPLRB4 1	prostate

cDNA Library	Designated Tissue for Library
Human nasopharyngeal carcinoma cell line HNE1	uncharacterized tissue
Homo sapiens normalized K562	uncharacterized tissue
Homo sapiens from hamster-human somatic cell-hybrid Q1Z	uncharacterized tissue
Homo sapiens brain fetus	uncharacterized tissue
Homo sapiens brain (M.Centola et al.)	uncharacterized tissue
Homo sapiens laryngeal cancer	uncharacterized tissue
PMA-induced HL60 cell subtraction library	uncharacterized tissue
Barstead aorta HPLRB6	vascular
Barstead colon HPLRB7	colon
Homo sapiens liver fetal	uncharacterized tissue
Human heart cDNA (CCLee)	uncharacterized tissue
Homo sapiens MCF7	uncharacterized tissue
Homo sapiens library (Carn G)	uncharacterized tissue
Homo sapiens placenta	uncharacterized tissue
Homo sapiens library (Jene Q)	uncharacterized tissue
Homo sapiens brain fetal (Jene Q)	uncharacterized tissue
Homo sapiens KM3	uncharacterized tissue
Homo sapiens fetal brain 18 weeks	uncharacterized tissue
Homo sapiens library (Sepe V)	uncharacterized tissue
Homo sapiens library (Yamagata K)	uncharacterized tissue
Homo sapiens mesothelium	uncharacterized tissue
Homo sapiens HBE-M	uncharacterized tissue
NCI_CGAP_HN5	head and neck
NCI_CGAP_HN6	head and neck
Homo sapiens (Yang,T.)	uncharacterized tissue
Human tonsils DDRT-PCR	uncharacterized tissue
HeLa SRIG (Synthetic retinoids induced genes)	uncharacterized tissue
Homo sapiens monocyte-derived macrophages	uncharacterized tissue
NCI_CGAP_HSC3	bone marrow
NCI_CGAP_HSC4	bone marrow
NCI_CGAP_Brn21	brain
Homo sapiens foreskin fibroblast	uncharacterized tissue
Homo sapiens library (Seranski P)	uncharacterized tissue
subtracted retina cDNA library	uncharacterized tissue
subtracted RPE cDNA library	uncharacterized tissue
NCI_CGAP_Ov35	ovary
NCI_CGAP_Brn52	brain
conorm	prostate
SAGE_OVCA432-2	ovary
SAGE_OV1063-3	ovary

cDNA Library	Designated Tissue for Library
Human pancreatic cancer cell line PANC1	uncharacterized tissue
bvtumor	prostate
tumor1	prostate
DU-145	uncharacterized tissue
PC-3	uncharacterized tissue
LnCap	uncharacterized tissue
bvnorm	prostate
ecnorm	prostate
mynorm	prostate
yodnorm	prostate
tumor2	prostate
misc	prostate
ECR1	uncharacterized tissue
SAGE_Duke_mhh-1	brain
Subtracted syncytiotrophoblast cDNA library	uncharacterized tissue
Homo sapiens lung (Chang L-Y)	uncharacterized tissue
Homo sapiens liver (Chang L-Y)	uncharacterized tissue
Homo sapiens brain (Chang L-Y)	uncharacterized tissue
Homo sapiens kidney (Chang L-Y)	uncharacterized tissue
Soares_Dieckgraefe_colon_NHCD	colon
Soares_Dieckgraefe_colon_NHUC	colon
Soares_thymus_NHFTh	thymus
Proliferating Erythroid Cells (LCB:ad library)	whole blood
Homo sapiens left brain fetus	uncharacterized tissue
J.Jerspeth, et al., Strategies 5 (1), 1992	uncharacterized tissue
564 (synonym: hfbr2)	brain
547 (synonym: hfbr1)	brain
566 (synonym: hfkd2)	kidney
434 (synonym: htes3)	testis
586 (synonym: hute1)	uterus
Homo sapiens library (Spurkland A)	uncharacterized tissue
Homo sapiens library (Yu Y)	uncharacterized tissue
SAGE_Duke_H341	brain
SAGE_HOSE_4	ovary
Human fetal heart cDNA library	uncharacterized tissue
Homo sapiens HL-60	uncharacterized tissue
Homo sapiens library (Nguyen MH)	uncharacterized tissue
Homo sapiens testis (Ladomery MR)	uncharacterized tissue
Homo sapiens brain fetal (Zhang LD)	uncharacterized tissue
Homo sapiens oc3-VGH	uncharacterized tissue

cDNA Library	Designated Tissue for Library
Homo sapiens umbilical vein	uncharacterized tissue
SAGE_OVP-5	ovary
Homo sapiens nasopharynx carcinoma	uncharacterized tissue
Homo sapiens nasopharynx	uncharacterized tissue
Homo sapiens liver	uncharacterized tissue
Homo sapiens ovary epithelium	uncharacterized tissue
Ovarian cancer cell lines SKOV3; 3AO	uncharacterized tissue
pancreatic cancer cell line PANC1, down-regulated by TGF-beta1	uncharacterized tissue
pancreatic cancer cell line PANC1, up-regulated by TGF-beta1	uncharacterized tissue
Homo sapiens kidney fetal	uncharacterized tissue
Homo sapiens HNE-1	uncharacterized tissue
Homo sapiens non-small-cell lung carcinoma	uncharacterized tissue
Homo sapiens BEAS-2B	uncharacterized tissue
NCI_CGAP_Ov18	ovary
Homo sapiens testis (Wang LF)	uncharacterized tissue
Human mRNA from cd34+ stem cells	whole blood
SAGE_LNCaP	prostate
SAGE_OVT-6	ovary
SAGE_H1126	brain
polydA/dT(-) colon cDNA library	uncharacterized tissue
NCI_CGAP_CML1	whole blood
NCI_CGAP_Co17	colon
NCI_CGAP_Pit1	pituitary gland
NCI_CGAP_Sar4	soft tissue
NCI_CGAP_Brn53	brain
NCI_CGAP_Br18	mammary gland
NCI_CGAP_Lu27	lung
NCI_CGAP_Lu28	lung
NCI_CGAP_GU1	genitourinary
NCI_CGAP_Kid13	kidney
NCI_CGAP_Mel15	skin
NCI_CGAP_Ov38	ovary
NCI_CGAP_Co18	colon
NCI_CGAP_Co19	colon
NCI_CGAP_Co20	colon
NCI_CGAP_Co21	colon
NCI_CGAP_Lu31	lung
CT0014	colon
HT0012	head and neck
HT0023	head and neck

cDNA Library	Designated Tissue for Library
ST0007	stomach
Human fetal brain Express Library	uncharacterized tissue
Homo sapiens nasopharyngeal carcinoma	uncharacterized tissue
NCI_CGAP_Pr1	prostate
NCI_CGAP_Pr2	prostate
NCI_CGAP_Pr3	prostate
NCI_CGAP_Pr4	prostate
NCI_CGAP_Ov1	ovary
NCI_CGAP_AR1	muscle
NCI_CGAP_Co1	colon
NCI_CGAP_GC1	germ cell
NCI_CGAP_GC2	germ cell
NCI_CGAP_Co2	colon
NCI_CGAP_Lu1	lung
NCI_CGAP_Sch1	peripheral nervous system
NCI_CGAP_AA1	adrenal cortex
NCI_CGAP_Co3	colon
NCI_CGAP_Pr5	prostate
NCI_CGAP_Pr6	prostate
NCI_CGAP_Pr7	prostate
NCI_CGAP_Pr8	prostate
NCI_CGAP_Pr9	prostate
NCI_CGAP_Pr10	prostate
NCI_CGAP_Pr11	prostate
NCI_CGAP_Pr12	prostate
NCI_CGAP_Li1	uncharacterized tissue
NCI_CGAP_Li2	uncharacterized tissue
NCI_CGAP_Lip2	adipose
NCI_CGAP_Kid1	kidney
NCI_CGAP_Thy1	thyroid
NCI_CGAP_Ov2	ovary
NCI_CGAP_Alv1	muscle
NCI_CGAP_Ew1	uncharacterized tissue
NCI_CGAP_Co4	colon
NCI_CGAP_Pr21	prostate
NCI_CGAP_Pr22	prostate
NCI_CGAP_Br1.1	mammary gland
NCI_CGAP_Br2	mammary gland
NCI_CGAP_Pr20	uncharacterized tissue
NCI_CGAP_Pr18	uncharacterized tissue

cDNA Library	Designated Tissue for Library
NCI_CGAP_Pr16	uncharacterized tissue
NCI_CGAP_GC3	germ cell
NCI_CGAP_GC4	germ cell
NCI_CGAP_Lei2	muscle
NCI_CGAP_Kid3	kidney
NCI_CGAP_Kid5	kidney
NCI_CGAP_Co9	colon
NCI_CGAP_Co10	colon
NCI_CGAP_Lu5	lung
NCI_CGAP_Co8	colon
NCI_CGAP_GCB1	lymph node
NCI_CGAP_Br3	mammary gland
NCI_CGAP_Gas1	stomach
NCI_CGAP_Co11	colon
NCI_CGAP_GC5	germ cell
NCI_CGAP_Pr23	prostate
NCI_CGAP_SS1	soft tissue
NCI_CGAP_Phe1	adrenal medulla
NCI_CGAP_Lar1	head and neck
NCI_CGAP_Pr4.1	prostate
NCI_CGAP_Co12	colon
NCI_CGAP_Kid6	kidney
NCI_CGAP_HSC1	bone marrow
NCI_CGAP_Lym3	lymph node
NCI_CGAP_Br4	uncharacterized tissue
NCI_CGAP_Br5	uncharacterized tissue
NCI_CGAP_Ov5	uncharacterized tissue
NCI_CGAP_Ov6	uncharacterized tissue
NCI_CGAP_Pr24	prostate
NCI_CGAP_Pr25	prostate
NCI_CGAP_Kid7	kidney
NCI_CGAP_Thym1	thymus
NCI_CGAP_CLL1	whole blood
NCI_CGAP_GCB0	lymph node
NCI_CGAP_CNS1	brain
NCI_CGAP_Ov8	ovary
NCI_CGAP_Br7	mammary gland
NCI_CGAP_Li5	liver
NCI_CGAP_PNS1	peripheral nervous system
NCI_CGAP_Lu6	lung

cDNA Library	Designated Tissue for Library
NCI_CGAP_HN1	head and neck
NCI_CGAP_HN2	head and neck
NCI_CGAP_Mel3	skin
NCI_CGAP_HN3	head and neck
NCI_CGAP_HN4	head and neck
NCI_CGAP_Lym5	lymph node
NCI_CGAP_Lym6	lymph node
NCI_CGAP_Eso2	esophagus
NCI_CGAP_Brn20	brain
Soares_testis_NHT	testis
Soares_pineal_gland_N3HPG	pineal gland
Soares_parathyroid_tumor_NbHPA	parathyroid
Soares_NFL_T_GBC_S1	pooled tissue
Soares_multiple_sclerosis_2NbHMSP	brain
Soares_fetal_liver_spleen_1NFLS_S1	pooled tissue
Soares_total_fetus_Nb2HF8_9w	whole body
Soares_NbHFB	brain
Soares_NhHMPu_S1	pooled tissue
NCI_CGAP_Brn23	brain
NCI_CGAP_Brn25	brain
Soares_senescent_fibroblasts_NbHSF	skin
Soares_fetal_heart_NbHH19W	heart
Soares_pregnant_uterus_NbHPU	uterus
Soares_placenta_8to9weeks_2NbHP8to9W	placenta
Soares_fetal_lung_NbHL19W	lung
SAGE_HCT116	colon
SAGE_Caco_2	colon
NCI_CGAP_Lu19	lung
NCI_CGAP_Kid8	kidney
NCI_CGAP_Co14	colon
NCI_CGAP_Ov23	ovary
NCI_CGAP_Ut1	uterus
NCI_CGAP_Ut2	uterus
NCI_CGAP_Ut3	uterus
NCI_CGAP_Ut4	uterus
NCI_CGAP_Brn35	brain
NCI_CGAP_Lym12	lymph node
NCI_CGAP_Pan1	pancreas
NCI_CGAP_Gas4	stomach
NCI_CGAP_Ov26	ovary

cDNA Library	Designated Tissue for Library
SAGE_Duke_H392	brain
SAGE_Duke_GBM_H1110	brain
SAGE_SW837	colon
SAGE_RKO	colon
NCI_CGAP_Pr28	prostate
NCI_CGAP_GC6	germ cell
SAGE_pooled_GBM	brain
SAGE_BB542_whitematter	brain
NCI_CGAP_Br12	mammary gland
NCI_CGAP_Ov31	ovary
NCI_CGAP_Ov32	ovary
NCI_CGAP_Ov33	ovary
NCI_CGAP_Ov34	ovary
SAGE_NHA(5th)	brain
SAGE_normal_pool(6th)	brain
NCI_CGAP_Kid11	kidney
NCI_CGAP_Kid12	kidney
NCI_CGAP_Co16	colon
NCI_CGAP_Lu24	lung
SAGE_NC1	colon
SAGE_NC2	colon
NCI_CGAP_Ov36	ovary
NCI_CGAP_Br13	mammary gland
NCI_CGAP_Br14	mammary gland
SAGE_Tu102	colon
SAGE_Tu98	colon
NCI_CGAP_Br15	mammary gland
NCI_CGAP_Br16	mammary gland
NCI_CGAP_Br17	mammary gland
NCI_CGAP_HSC2	bone marrow
NCI_CGAP_Ov37	ovary
NCI_CGAP_Lu25	lung
NCI_CGAP_Lu26	lung
Soares_NSF_F8_9W_OT_PA_P_S1	pooled tissue
SAGE_ES2-1	ovary
Fetal Brain, Bento Soares	brain
Hippocampus, Ruben Moreno	brain
Temporal Cortex, Stratagene (cat.#935205)	cerebrum
Whole Brain, Clontech mRNA, Ruben Moreno	brain
Subtracted Hippocampus, Stratagene (cat.#936205)	brain

cDNA Library	Designated Tissue for Library
Subtracted human retinal pigment epithelium (RPE)	retina
Stratagene cDNA library Human heart, cat#936208	heart
Liver HepG2 cell line.	liver
B, Human Liver tissue	liver
H, Human adult Brain Cortex tissue	cerebrum
JG, Human foetal Kidney tissue	uncharacterized tissue
O, Human adult Bone tissue	uncharacterized tissue
P, Human foetal Brain Whole tissue	brain
S, Human foetal Adrenals tissue	endocrine
T, Human adult Rhabdomyosarcoma cell-line	muscle
TEST1, Human adult Testis tissue	testis
V, Human Placenta tissue	placenta
W, Human Liver tissue	uncharacterized tissue
X, Human Liver tissue	uncharacterized tissue
Y, Human Placenta tissue	placenta
Stratagene cDNA library Human fibroblast, cat#937212	uncharacterized tissue
Human Infant Brain, Bento Soares	uncharacterized tissue
GeneTrack, 4p16.3 JM Rommens	pooled tissue
Stratagene cat#937212 (1992)	uncharacterized tissue
Fetal brain, Stratagene (cat#936206)	brain
Hippocampus, prescreened, Stratagene (cat#936205)	uncharacterized tissue
Hippocampus, random, Stratagene (cat#936205)	uncharacterized tissue
Hippocampus, subtracted, Stratagene (cat#936205)	uncharacterized tissue
Stratagene human foetal retina	uncharacterized tissue
b4HB3MA Cot8-HAP-Ft	brain
Chromosome 9 exon	uncharacterized tissue
Human pancreatic islet	pancreatic islet
Heart	heart
Atrium cDNA library Human heart	heart
Hippocampus, Stratagene (cat.#936205)	cerebrum
Chromosome 9 exon II	uncharacterized tissue
HL 1011	uncharacterized tissue
Human K562 erythroleukemic cells	uncharacterized tissue
Fetal brain, Stratagene	brain
Normalized infant brain, Bento Soares	brain
Infant Brain, Bento Soares	brain
Infant brain, Bento Soares	brain
Human whole brain	uncharacterized tissue
Human CGM	uncharacterized tissue
Human promyelocyte	bone marrow

cDNA Library	Designated Tissue for Library
Human aortic endothelium	vascular
Human colon mucosa	colon
1HB3MK	uncharacterized tissue
2HB3MA	uncharacterized tissue
2HB3MK	uncharacterized tissue
2HFBAK19-Cot-inf-FTA	uncharacterized tissue
4HFLSK20	uncharacterized tissue
b4HB3MA-Cot0.38-HAP-B	uncharacterized tissue
b4HB3MA-Cot109+10-Bio	uncharacterized tissue
b4HB3MA-Cot109+103+85-Bio	uncharacterized tissue
b4HB3MA-Cot109+103-Bio	uncharacterized tissue
b4HB3MA-Cot12-HAP-B	uncharacterized tissue
b4HB3MA-Cot12-HAP-Ft	uncharacterized tissue
b4HB3MA-Cot14.5	uncharacterized tissue
b4HB3MA-Cot18-Bio	uncharacterized tissue
b4HB3MA-Cot51.5-HAP-Ft	uncharacterized tissue
b4HB3MK	uncharacterized tissue
b4HFLSK20	uncharacterized tissue
Cot250Ft-b4HB3MA	uncharacterized tissue
HMSWMYK	uncharacterized tissue
HWM42YA	uncharacterized tissue
N-b4HB3MA-Cot109	uncharacterized tissue
N2HFBA19	uncharacterized tissue
NHB3MK	uncharacterized tissue
3HFLSK20	pooled tissue
4HB3MK	uncharacterized tissue
4HFLSA20	uncharacterized tissue
b4HB3MA Cot109+10-Bio	uncharacterized tissue
b4HB3MA Cot109+103+85-Bio	uncharacterized tissue
b4HB3MA Cot109+103-Bio	uncharacterized tissue
b4HB3MA Cot14.5	uncharacterized tissue
b4HB3MA Cot18-Bio	uncharacterized tissue
Cot1374Ft 4HB3MA	uncharacterized tissue
Cot250Ft b4HB3MA	uncharacterized tissue
Cot274 N4HB3MA	uncharacterized tissue
HB3MK	uncharacterized tissue
N-b4HB3MA Cot109	uncharacterized tissue
N3HFLSK20	pooled tissue
N4HB3MK	uncharacterized tissue
b4HB3MA	uncharacterized tissue

cDNA Library	Designated Tissue for Library
b4HB3MA-Cot109-Bio	uncharacterized tissue
HB3MA	uncharacterized tissue
b4HB3MA-FT20N-b4HB3MA	uncharacterized tissue
b4HB3MA-Cot0.38-HAP-Ft-6	uncharacterized tissue
Human brain striatum	brain
Human colorectal cancer	colon
4HB3MA-Bio	uncharacterized tissue
ClonTech HL 1065a	brain
Human epidermal keratinocyte	skin
Human placenta	placenta
NEM subtracted human fetal kidney cDNA	kidney
HTCDL1	thymus
Human islet	uncharacterized tissue
Testis 1	testis
Testis 2	testis
BL29 Burkitt's lymphoma, Pascalis Sideras	uncharacterized tissue
CD34+DIRECTIONAL	uncharacterized tissue
Clontech adult human fat cell library HL1108A	uncharacterized tissue
Human Genomic	uncharacterized tissue
SAGE_Duke_H247_normal	brain
SAGE_Duke_H247_Hypoxia	brain
SAGE_Duke-H988	brain
NCI_CGAP_Sub3	uncharacterized tissue
radiation induced genes library	uncharacterized tissue
Homo sapiens endometrium adult	uncharacterized tissue
Human umbilical venous cord	uncharacterized tissue
Homo sapiens u937	uncharacterized tissue
Homo sapiens differential display product	uncharacterized tissue
SAGE_Duke_post_crisis_fibroblasts	gastrointestinal tract
Myosin light chain	uncharacterized tissue
cDNA converted from mRNA of normal human small intestine	uncharacterized tissue
cDNA converted from thymus mRNA	uncharacterized tissue
cDNA converted from placenta mRNA	uncharacterized tissue
cDNA converted from testis mRNA	uncharacterized tissue
cDNA converted from bone marrow mRNA	uncharacterized tissue
cDNA converted from trachea mRNA	uncharacterized tissue
cDNA converted from colon mRNA	uncharacterized tissue
NIH_MGC_2	uncharacterized tissue
NIH_MGC_3	lymph node

cDNA Library	Designated Tissue for Library
NIH_MGC_4	cervix
NIH_MGC_35	cervix
NIH_MGC_7	lung
NIH_MGC_5	cervix
NIH_MGC_8	lymph node
NIH_MGC_14	kidney
NIH_MGC_15	colon
NIH_MGC_20	skin
NIH_MGC_21	placenta
NIH_MGC_10	placenta
NIH_MGC_12	cervix
BT0041	mammary gland
BT0043	mammary gland
BT0046	mammary gland
BT0057	mammary gland
BT0068	mammary gland
BT0074	mammary gland
BT0077	mammary gland
BT0078	mammary gland
BT0091	mammary gland
BT0152	mammary gland
BT0214	mammary gland
BT0217	mammary gland
BT0228	mammary gland
BT0233	mammary gland
BT0246	mammary gland
BT0247	mammary gland
CT0027	colon
CT0063	colon
CT0066	colon
CT0068	colon
CT0084	colon
CT0094	colon
CT0102	colon
CT0106	colon
CT0113	colon
CT0127	colon
CT0128	colon
CT0129	colon
CT0131	colon

cDNA Library	Designated Tissue for Library
CT0145	colon
CT0149	colon
CT0151	colon
CT0153	colon
CT0155	colon
CT0159	colon
CT0161	colon
CT0172	colon
CT0197	colon
CT0199	colon
HT0018	head and neck
HT0030	head and neck
HT0031	head and neck
HT0032	head and neck
HT0034	head and neck
HT0038	head and neck
HT0059	head and neck
HT0060	head and neck
HT0061	head and neck
HT0062	head and neck
HT0065	head and neck
HT0066	head and neck
HT0067	head and neck
HT0070	head and neck
HT0074	head and neck
HT0091	head and neck
HT0105	head and neck
HT0106	head and neck
HT0111	head and neck
HT0112	head and neck
HT0113	head and neck
HT0114	head and neck
HT0115	head and neck
HT0116	head and neck
HT0118	head and neck
HT0123	head and neck
HT0125	head and neck
HT0134	head and neck
ST0008	stomach
ST0011	stomach

cDNA Library	Designated Tissue for Library
ST0012	stomach
ST0013	stomach
ST0019	stomach
ST0032	stomach
ST0033	stomach
ST0036	stomach
ST0065	stomach
ST0066	stomach
ST0067	stomach
ST0069	stomach
ST0070	stomach
ST0071	stomach
ST0072	stomach
ST0074	stomach
ST0092	stomach
ST0093	stomach
ST0094	stomach
ST0115	stomach
ST0120	stomach
NCI_CGAP_Thy4	thyroid
NCI_CGAP_RDF1	uncharacterized tissue
NCI_CGAP_RDF2	prostate
SAGE_Duke_precrisis_fibroblasts	gastrointestinal tract
NIH_MGC_36	lymph node
NIH_MGC_37	lymph node
NIH_MGC_38	lymph node
human islet cDNA differential display	uncharacterized tissue
NCI_CGAP_Thy5	uncharacterized tissue
NCI_CGAP_Thy6	thyroid
NCI_CGAP_Thy7	thyroid
NCI_CGAP_Thy8	thyroid
NCI_CGAP_Thy10	thyroid
NCI_CGAP_Sub4	uncharacterized tissue
SAGE_DCIS_2	mammary gland
SAGE_Br_N	mammary gland
SAGE_A+	prostate
Uni-ZAP XR retinal pigment epithelium	uncharacterized tissue
SAGE_IOSE29-11	ovary
Breast Cancer Associated Library	uncharacterized tissue
NIH_MGC_17	muscle

cDNA Library	Designated Tissue for Library
cDNA Library from Human Neuroblastoma SK-N-SH	uncharacterized tissue
cDNA Library from Human Neuroepithelioma SK-N-MC	uncharacterized tissue
NCI_CGAP_Sub5	uncharacterized tissue
NCI_CGAP_Sub6	uncharacterized tissue
SAGE_Duke_H1043	brain
gastric epithelial cell GES-1	uncharacterized tissue
NCI_CGAP_Co23	colon
NCI_CGAP_HN14	uncharacterized tissue
NCI_CGAP_HN15	uncharacterized tissue
NCI_CGAP_HN16	head and neck
cDNA Library from rIL-2 activated lymphocytes	uncharacterized tissue
NCI_CGAP_Brn41	brain
Homo sapiens LNCaP prostate cancer	uncharacterized tissue
LoVo express library before ATRA and D3 induced	uncharacterized tissue
LoVo express library after ATRA and D3 induced	uncharacterized tissue
gastric carcinoma cell GC7901	uncharacterized tissue
NIH_MGC_9	ovary
NCI_CGAP_Thy3	thyroid
NCI_CGAP_Ov41	ovary
NCI_CGAP_Pan3	pancreas
NCI_CGAP_HN13	head and neck
NCI_CGAP_Adr1	endocrine
NCI_CGAP_Ut7	uterus
NCI_CGAP_Sub7	uncharacterized tissue
SAGE_Duke_1273	cerebellum
Homo sapiens endothelial cell	uncharacterized tissue
human melanoma microcell hybrid line, MelJuSo-28(1n)	uncharacterized tissue
human melanoma line, MelJuSo	uncharacterized tissue
FIBHSVI	uncharacterized tissue
FIBCTGF	uncharacterized tissue
FIBHAV	uncharacterized tissue
SAGE_TSU	prostate
NIH_MGC_16	eye
NIH_MGC_19	brain
Homo sapiens inflammatory skin	uncharacterized tissue
Homo sapiens psoriatic skin	uncharacterized tissue
LTI_NFL011_NBC1	brain
LTI_NFL004_NBC2	brain
LTI_NFL003_NBC3	brain
LTI_NFL001_NBC4	brain

cDNA Library	Designated Tissue for Library
LTI_FL011_BC1	brain
LTI_FL012_TC1	uncharacterized tissue
LTI_FL002_PL1	placenta
LTI_FL013_FBrn1	brain
NCI_CGAP_Thy11	thyroid
NCI_CGAP_Thy12	thyroid
NIH_MGC_50	lymph node
NIH_MGC_51	lymph node
NIH_MGC_52	lymph node
SAGE_A-	prostate
Human fetal heart cDNA library (Liu,L.and Gu,J.)	uncharacterized tissue
Immunomagnetically sorted breast epithelial cells (differential display)	uncharacterized tissue
SAGE_Duke_thalamus	brain
Homo sapiens prostate adult	uncharacterized tissue
Human differential display products	uncharacterized tissue
SAGE_Duke_H1020	brain
Homo sapiens PC-3M high metastasis subline 1E8	uncharacterized tissue
Homo sapiens PC-3M low metastasis subline 2B4	uncharacterized tissue
CT0114	colon
CT0117	colon
CT0135	colon
CT0141	colon
CT0143	colon
CT0160	colon
CT0163	colon
CT0165	colon
CT0167	colon
CT0176	colon
CT0177	colon
CT0196	colon
CT0198	colon
CT0200	colon
CT0201	colon
CT0202	colon
CT0203	colon
HT0082	head and neck
HT0107	head and neck
HT0117	head and neck
HT0124	head and neck
HT0128	head and neck

cDNA Library	Designated Tissue for Library
HT0130	head and neck
HT0136	head and neck
HT0137	head and neck
HT0138	head and neck
HT0139	head and neck
HT0140	head and neck
HT0141	head and neck
ST0078	stomach
ST0080	stomach
ST0081	stomach
ST0114	stomach
SAGE_Duke_BB542_normal_cerebellum	cerebellum
BN0001	mammary gland
BN0002	mammary gland
BN0003	mammary gland
BN0004	mammary gland
BN0005	mammary gland
BN0012	mammary gland
BN0013	mammary gland
BN0014	mammary gland
BN0015	mammary gland
BT0019	mammary gland
BT0040	mammary gland
BT0155	mammary gland
BT0248	mammary gland
BT0249	mammary gland
BT0252	mammary gland
BT0253	mammary gland
BT0254	mammary gland
BT0255	mammary gland
BT0256	mammary gland
BT0257	mammary gland
BT0258	mammary gland
BT0259	mammary gland
BT0260	mammary gland
BT0263	mammary gland
BT0264	mammary gland
BT0265	mammary gland
BT0266	mammary gland
BT0274	mammary gland

cDNA Library	Designated Tissue for Library
BT0275	mammary gland
BT0276	mammary gland
BT0277	mammary gland
BT0278	mammary gland
BT0279	mammary gland
BT0280	mammary gland
BT0281	mammary gland
BT0282	mammary gland
BT0283	mammary gland
BT0284	mammary gland
BT0286	mammary gland
BT0287	mammary gland
BT0291	mammary gland
BT0292	mammary gland
BT0293	mammary gland
BT0294	mammary gland
BT0295	mammary gland
BT0296	mammary gland
BT0297	mammary gland
BT0300	mammary gland
BT0301	mammary gland
BT0302	mammary gland
BT0303	mammary gland
BT0304	mammary gland
BT0305	mammary gland
BT0306	mammary gland
BT0308	mammary gland
BT0309	mammary gland
BT0310	mammary gland
BT0311	mammary gland
BT0312	mammary gland
BT0313	mammary gland
BT0314	mammary gland
BT0316	mammary gland
BT0317	mammary gland
BT0318	mammary gland
BT0319	mammary gland
BT0320	mammary gland
BT0321	mammary gland
BT0322	mammary gland

cDNA Library	Designated Tissue for Library
BT0324	mammary gland
BT0325	mammary gland
BT0326	mammary gland
BT0327	mammary gland
BT0329	mammary gland
BT0332	mammary gland
BT0333	mammary gland
BT0335	mammary gland
BT0336	mammary gland
BT0338	mammary gland
BT0339	mammary gland
BT0340	mammary gland
BT0341	mammary gland
BT0346	mammary gland
BT0348	mammary gland
BT0349	mammary gland
BT0350	mammary gland
BT0362	mammary gland
BT0364	mammary gland
BT0365	mammary gland
BT0366	mammary gland
BT0367	mammary gland
BT0368	mammary gland
BT0375	mammary gland
BT0377	mammary gland
BT0379	mammary gland
BT0380	mammary gland
BT0381	mammary gland
BT0382	mammary gland
BT0383	mammary gland
BT0385	mammary gland
BT0386	mammary gland
BT0387	mammary gland
BT0388	mammary gland
BT0391	mammary gland
BT0393	mammary gland
BT0396	mammary gland
BT0397	mammary gland
BT0401	mammary gland
BT0500	mammary gland

cDNA Library	Designated Tissue for Library
BT0502	mammary gland
BT0503	mammary gland
BT0505	mammary gland
BT0506	mammary gland
BT0508	mammary gland
BT0509	mammary gland
BT0510	mammary gland
BT0511	mammary gland
BT0513	mammary gland
BT0515	mammary gland
BT0518	mammary gland
BT0519	mammary gland
BT0520	mammary gland
BT0522	mammary gland
BT0523	mammary gland
BT0524	mammary gland
BT0531	mammary gland
BT0533	mammary gland
BT0534	mammary gland
BT0536	mammary gland
BT0537	mammary gland
BT0538	mammary gland
BT0539	mammary gland
BT0542	mammary gland
BT0543	mammary gland
BT0545	mammary gland
BT0546	mammary gland
BT0547	mammary gland
BT0548	mammary gland
BT0549	mammary gland
BT0552	mammary gland
BT0556	mammary gland
BT0558	mammary gland
BT0560	mammary gland
BT0567	mammary gland
BT0568	mammary gland
CT0017	colon
CT0056	colon
CT0057	colon
CT0058	colon

cDNA Library	Designated Tissue for Library
CT0059	colon
CT0061	colon
CT0115	colon
CT0116	colon
CT0121	colon
CT0147	colon
CT0152	colon
CT0173	colon
CT0175	colon
CT0178	colon
CT0179	colon
CT0180	colon
CT0181	colon
CT0186	colon
CT0189	colon
CT0192	colon
CT0194	colon
CT0195	colon
CT0204	colon
CT0205	colon
CT0206	colon
CT0207	colon
CT0208	colon
CT0209	colon
CT0210	colon
CT0212	colon
CT0213	colon
CT0214	colon
CT0219	colon
CT0220	colon
CT0222	colon
CT0223	colon
CT0224	colon
CT0225	colon
CT0227	colon
CT0236	colon
CT0237	colon
CT0238	colon
CT0239	colon
CT0240	colon

cDNA Library	Designated Tissue for Library
CT0241	colon
CT0242	colon
CT0243	colon
CT0244	colon
CT0245	colon
CT0246	colon
CT0247	colon
CT0248	colon
CT0249	colon
CT0250	colon
CT0251	colon
CT0252	colon
CT0253	colon
CT0254	colon
CT0255	colon
CT0256	colon
CT0257	colon
CT0260	colon
CT0261	colon
CT0263	colon
CT0264	colon
CT0265	colon
CT0266	colon
CT0267	colon
CT0268	colon
CT0275	colon
CT0276	colon
CT0277	colon
CT0278	colon
CT0279	colon
CT0280	colon
CT0281	colon
CT0282	colon
CT0283	colon
CT0284	colon
CT0285	colon
CT0286	colon
CT0287	colon
CT0288	colon
CT0289	colon

cDNA Library	Designated Tissue for Library
CT0290	colon
CT0291	colon
CT0292	colon
CT0293	colon
CT0294	colon
CT0295	colon
CT0297	colon
CT0298	colon
CT0299	colon
CT0300	colon
CT0301	colon
CT0302	colon
CT0303	colon
CT0304	colon
CT0305	colon
CT0306	colon
CT0307	colon
CT0308	colon
CT0309	colon
CT0311	colon
CT0312	colon
CT0313	colon
CT0314	colon
CT0315	colon
CT0317	colon
CT0318	colon
CT0320	colon
CT0321	colon
CT0322	colon
CT0323	colon
CT0324	colon
CT0325	colon
CT0326	colon
CT0328	colon
CT0329	colon
CT0330	colon
SN0034	stomach
SN0035	stomach
SN0036	stomach
SN0037	stomach

cDNA Library	Designated Tissue for Library
SN0038	stomach
SN0039	stomach
SN0040	stomach
SN0042	stomach
SN0043	stomach
SN0044	stomach
SN0045	stomach
SN0046	stomach
SN0047	stomach
SN0052	stomach
SN0056	stomach
SN0060	stomach
SN0061	stomach
SN0062	stomach
SN0063	stomach
SN0064	stomach
SN0065	stomach
SN0066	stomach
SN0067	stomach
SN0068	stomach
SN0070	stomach
SN0071	stomach
SN0072	stomach
SN0073	stomach
SN0074	stomach
SN0075	stomach
PT0010	uncharacterized tissue
PT0011	uncharacterized tissue
PT0022	uncharacterized tissue
PT0034	uncharacterized tissue
PT0039	uncharacterized tissue
PT0051	uncharacterized tissue
PT0052	uncharacterized tissue
PT0053	uncharacterized tissue
PT0054	uncharacterized tissue
PT0073	uncharacterized tissue
OT0005	ovary
OT0006	ovary
OT0007	ovary
OT00078	ovary

cDNA Library	Designated Tissue for Library
OT0008	ovary
OT0009	ovary
OT0010	ovary
OT0011	ovary
OT0012	ovary
OT0014	ovary
OT0015	ovary
OT0019	ovary
OT0022	ovary
OT0024	ovary
OT0025	ovary
OT0028	ovary
OT0029	ovary
OT0030	ovary
OT0031	ovary
OT0032	ovary
OT0033	ovary
OT0035	ovary
OT0036	ovary
OT0037	ovary
OT0039	ovary
OT0040	ovary
OT0043	ovary
OT0044	ovary
OT0045	ovary
OT0046	ovary
OT0048	ovary
OT0049	ovary
OT0050	ovary
OT0051	ovary
OT0053	ovary
OT0054	ovary
OT0055	ovary
OT0057	ovary
OT0058	ovary
OT0062	ovary
OT0063	ovary
OT0064	ovary
OT0065	ovary
OT0066	ovary

cDNA Library	Designated Tissue for Library
OT0067	ovary
OT0068	ovary
OT0069	ovary
OT0071	ovary
OT0072	ovary
OT0073	ovary
OT0075	ovary
OT0076	ovary
OT00778	ovary
OT0078	ovary
OT0079	ovary
OT0080	ovary
OT0081	ovary
OT0082	ovary
OT0083	ovary
OT0084	ovary
OT0086	ovary
OT0088	ovary
OT0089	ovary
OT0091	ovary
OT0092	ovary
OT0093	ovary
OT0094	ovary
OT0095	ovary
OT0096	ovary
OT0708	ovary
Human matrix tissue expression library	uncharacterized tissue
NN0001	cerebrum
NN0003	cerebrum
NN0004	cerebrum
NN0005	cerebrum
NN0006	cerebrum
NN0007	cerebrum
NN0008	cerebrum
NN0009	cerebrum
NN0010	cerebrum
NN0011	cerebrum
NN0012	cerebrum
NN0021	cerebrum
NN0023	cerebrum

cDNA Library	Designated Tissue for Library
NN0024	cerebrum
NN0025	cerebrum
NN0026	cerebrum
NN0027	cerebrum
NN0028	cerebrum
NN0030	cerebrum
NN0031	cerebrum
NN0032	cerebrum
NN0033	cerebrum
NN0034	cerebrum
NN0035	cerebrum
NN0036	cerebrum
NN0037	cerebrum
NN0038	cerebrum
NN0039	cerebrum
NN0040	cerebrum
NN0041	cerebrum
NN0046	cerebrum
NN0047	cerebrum
NN0048	cerebrum
NN0049	cerebrum
NN0050	cerebrum
NN0051	cerebrum
NN0052	cerebrum
NN0053	cerebrum
NN0054	cerebrum
NN0055	cerebrum
NN0056	cerebrum
NN0057	cerebrum
NN0058	cerebrum
NN0059	cerebrum
NN0060	cerebrum
NN0061	cerebrum
NN0062	cerebrum
NN0063	cerebrum
NN0064	cerebrum
NN0066	cerebrum
NN0067	cerebrum
NN0068	cerebrum
NN0070	cerebrum

cDNA Library	Designated Tissue for Library
NN0071	cerebrum
NN0072	cerebrum
NN0073	cerebrum
NN0074	cerebrum
NN0075	cerebrum
NN0076	cerebrum
NN0078	cerebrum
NN0079	cerebrum
NN0080	cerebrum
NN0081	cerebrum
NN0082	cerebrum
NN0083	cerebrum
NN0084	cerebrum
NN0087	cerebrum
NN0088	cerebrum
NN0089	cerebrum
NN0090	cerebrum
NN0091	cerebrum
NN0092	cerebrum
NN1002	cerebrum
NN1003	cerebrum
NN1004	cerebrum
NN1005	cerebrum
NN1006	cerebrum
NN1008	cerebrum
NN1009	cerebrum
NN1010	cerebrum
NN1011	cerebrum
NN1012	cerebrum
NN1013	cerebrum
NN1014	cerebrum
NN1015	cerebrum
NN1018	cerebrum
NN1020	cerebrum
NN1021	cerebrum
NN1022	cerebrum
NN1023	cerebrum
NN1024	cerebrum
NN1025	cerebrum
NN1026	cerebrum

cDNA Library	Designated Tissue for Library
NN1027	cerebrum
NN1028	cerebrum
NN1029	cerebrum
NN1030	cerebrum
NN1031	cerebrum
NN1032	cerebrum
NN1037	cerebrum
NN1038	cerebrum
NN1039	cerebrum
NN1040	cerebrum
NN1041	cerebrum
NN1043	cerebrum
NN1044	cerebrum
NN1046	cerebrum
NN1047	cerebrum
NN1048	cerebrum
NN1050	cerebrum
NN1055	cerebrum
NN1059	cerebrum
NN1060	cerebrum
NN1061	cerebrum
NN1062	cerebrum
NN1063	cerebrum
NN1064	cerebrum
NN1065	cerebrum
NN1066	cerebrum
NN1067	cerebrum
NN1071	cerebrum
NN1072	cerebrum
NN1073	cerebrum
NN1080	cerebrum
NN1082	cerebrum
NN1083	cerebrum
NN1084	cerebrum
NN1085	cerebrum
NN1086	cerebrum
NN1089	cerebrum
NT0002	brain
NT0003	brain
NT0004	brain

cDNA Library	Designated Tissue for Library
NT0005	brain
NT0006	brain
NT0007	brain
NT0010	brain
NT0011	brain
NT0012	brain
NT0013	brain
NT0014	brain
NT0015	brain
NT0020	brain
NT0027	brain
NT0028	brain
NT0029	brain
NT0031	brain
NT0032	brain
NT0033	brain
NT0035	brain
NT0036	brain
NT0037	brain
NT0038	brain
NT0039	brain
NT0040	brain
NT0045	brain
NT0052	brain
NT0053	brain
NT0054	brain
NT0057	brain
NT0058	brain
NT0072	brain
NT0073	brain
NT0075	brain
NT0076	brain
NT0077	brain
NT0078	brain
NT0079	brain
NT0080	brain
NT0081	brain
NT0082	brain
NT0083	brain
NT0084	brain

cDNA Library	Designated Tissue for Library
NT0088	brain
NT0089	brain
NT0090	brain
NT0118	brain
NT0119	brain
NT0120	brain
Homo sapiens liver 16 week fetus	uncharacterized tissue
LST1	uncharacterized tissue
DT0023	uncharacterized tissue
DT0024	uncharacterized tissue
DT0033	uncharacterized tissue
DT0038	uncharacterized tissue
DT0047	uncharacterized tissue
DT0048	uncharacterized tissue
DT0059	uncharacterized tissue
DT0060	uncharacterized tissue
DT0066	uncharacterized tissue
DT0068	uncharacterized tissue
DT0071	uncharacterized tissue
DT0077	uncharacterized tissue
DT0078	uncharacterized tissue
DT0079	uncharacterized tissue
MAGE resequences, MAGA	uncharacterized tissue
EN0001	lung
EN0002	lung
EN0003	lung
EN0004	lung
EN0005	lung
EN0006	lung
EN0007	lung
EN0008	lung
EN0011	lung
EN0012	lung
EN0013	lung
EN0014	lung
EN0020	lung
EN0022	lung
EN0024	lung
EN0027	lung
EN0032	lung

cDNA Library	Designated Tissue for Library
EN0040	lung
ET0001	lung
ET0017	lung
ET0018	lung
ET0019	lung
ET0020	lung
ET0021	lung
ET0022	lung
ET0023	lung
ET0024	lung
ET0027	lung
ET0039	lung
ET0040	lung
ET0041	lung
MT0002	whole blood
MT0003	whole blood
MT0004	whole blood
MT0005	whole blood
MT0006	whole blood
MT0007	whole blood
MT0008	whole blood
MT0009	whole blood
MT0010	whole blood
MT0011	whole blood
MT0012	whole blood
MT0013	whole blood
MT0014	whole blood
MT0015	whole blood
FN0040	prostate
FN0041	prostate
FN0042	prostate
FN0043	prostate
FN0044	prostate
FT0001	prostate
FT0002	prostate
FT0004	prostate
FT0005	prostate
FT0006	prostate
NIH_MGC_53	genitourinary
NIH_MGC_54	bone marrow

cDNA Library	Designated Tissue for Library
NIH_MGC_55	bone marrow
NIH_MGC_56	brain
NIH_MGC_57	brain
NIH_MGC_58	kidney
NIH_MGC_59	salivary gland
NIH_MGC_60	prostate
NIH_MGC_61	testis
NIH_MGC_62	skin
MAGE resequences, MAGB	uncharacterized tissue
MAGE resequences, MAGC	uncharacterized tissue
MAGE resequences, MAGD	uncharacterized tissue
MAGE resequences, MAGE	uncharacterized tissue
MAGE resequences, MAGF	uncharacterized tissue
MAGE resequences, MAGG	uncharacterized tissue
MAGE resequences, MAGH	uncharacterized tissue
MAGE resequences, MAGI	uncharacterized tissue
MAGE resequences, MAGJ	uncharacterized tissue
MAGE resequences, MAGK	uncharacterized tissue
MAGE resequences, MAGL	uncharacterized tissue
MAGE resequences, MAGM	uncharacterized tissue
MAGE resequences, MAGN	uncharacterized tissue
MAGE resequences, MAGO	uncharacterized tissue
MAGE resequences, MAGP	uncharacterized tissue
HN0001	head and neck
HN0002	head and neck
HN0003	head and neck
HN0004	head and neck
HN0005	head and neck
HN0006	head and neck
HN0007	head and neck
HN0008	head and neck
HN0009	head and neck
HN0010	head and neck
HN0011	head and neck
HN0012	head and neck
HN0013	head and neck
HN0014	head and neck
HN0015	head and neck
BN0006	mammary gland
BN0007	mammary gland

cDNA Library	Designated Tissue for Library
BN0022	mammary gland
BN0023	mammary gland
BN0024	mammary gland
BN0030	mammary gland
BN0032	mammary gland
BN0033	mammary gland
BN0034	mammary gland
BN0035	mammary gland
BN0036	mammary gland
BN0037	mammary gland
BN0038	mammary gland
BN0039	mammary gland
BN0040	mammary gland
BN0041	mammary gland
BN0042	mammary gland
BN0043	mammary gland
BN0044	mammary gland
BN0045	mammary gland
BN0046	mammary gland
BN0047	mammary gland
BN0048	mammary gland
BN0049	mammary gland
BN0050	mammary gland
BN0051	mammary gland
BN0052	mammary gland
BN0053	mammary gland
BN0054	mammary gland
BN0055	mammary gland
BN0056	mammary gland
BN0057	mammary gland
BN0058	mammary gland
BN0059	mammary gland
BN0060	mammary gland
BN0063	mammary gland
BN0064	mammary gland
BN0065	mammary gland
BN0066	mammary gland
BN0067	mammary gland
BN0068	mammary gland
BN0070	mammary gland

cDNA Library	Designated Tissue for Library
BN0072	mammary gland
BN0073	mammary gland
BN0074	mammary gland
BN0075	mammary gland
BN0077	mammary gland
BN0078	mammary gland
BN0080	mammary gland
BN0081	mammary gland
BN0082	mammary gland
BN0083	mammary gland
BN0084	mammary gland
BN0085	mammary gland
BN0087	mammary gland
BN0090	mammary gland
BN0091	mammary gland
BN0093	mammary gland
BN0095	mammary gland
BN0096	mammary gland
BN0097	mammary gland
BN0101	mammary gland
BN0102	mammary gland
BN0103	mammary gland
BN0105	mammary gland
BN0106	mammary gland
BN0109	mammary gland
BN0111	mammary gland
BN0112	mammary gland
BN0113	mammary gland
BN0114	mammary gland
BN0115	mammary gland
BN0116	mammary gland
BN0117	mammary gland
BN0118	mammary gland
BN0120	mammary gland
BN0121	mammary gland
BN0123	mammary gland
BN0124	mammary gland
BN0125	mammary gland
BN0126	mammary gland
BN0127	mammary gland

cDNA Library	Designated Tissue for Library
BN0128	mammary gland
BN0129	mammary gland
BN0130	mammary gland
BN0131	mammary gland
BN0132	mammary gland
BN0133	mammary gland
BN0136	mammary gland
BN0137	mammary gland
BN0138	mammary gland
BN0139	mammary gland
BN0140	mammary gland
BN0141	mammary gland
BN0142	mammary gland
BN0143	mammary gland
BN0144	mammary gland
BN0146	mammary gland
BN0147	mammary gland
BN0148	mammary gland
BN0151	mammary gland
BN0153	mammary gland
BN0154	mammary gland
BN0155	mammary gland
BN0157	mammary gland
BN0158	mammary gland
BN0159	mammary gland
BN0160	mammary gland
BN0161	mammary gland
BN0162	mammary gland
BN0163	mammary gland
BN0164	mammary gland
BN0165	mammary gland
BN0167	mammary gland
BN0169	mammary gland
BN0170	mammary gland
BN0171	mammary gland
BN0172	mammary gland
BN0173	mammary gland
BN0174	mammary gland
BN0175	mammary gland
BN0176	mammary gland

cDNA Library	Designated Tissue for Library
BN0180	mammary gland
BN0181	mammary gland
BN0182	mammary gland
BN0183	mammary gland
BN0185	mammary gland
BN0186	mammary gland
BN0189	mammary gland
BN0190	mammary gland
BN0192	mammary gland
BN0193	mammary gland
BN0194	mammary gland
BN0195	mammary gland
BN0196	mammary gland
BN0197	mammary gland
BN0198	mammary gland
BN0217	mammary gland
BN0218	mammary gland
BN0219	mammary gland
BN0220	mammary gland
BN0221	mammary gland
BN0222	mammary gland
BN0223	mammary gland
BN0224	mammary gland
BN0225	mammary gland
BN0228	mammary gland
BN0230	mammary gland
BN0231	mammary gland
BN1057	mammary gland
BN1058	mammary gland
Human fetal brain cDNA	uncharacterized tissue
SAGE_Panc_91-16113	pancreas
SAGE_Panc_96-6252	pancreas
NCI_CGAP_Skn1	skin
BT0271	mammary gland
BT0273	mammary gland
BT0337	mammary gland
BT0347	mammary gland
BT0369	mammary gland
BT0370	mammary gland
BT0371	mammary gland

cDNA Library	Designated Tissue for Library
BT0374	mammary gland
BT0389	mammary gland
BT0404	mammary gland
BT0405	mammary gland
BT0406	mammary gland
BT0414	mammary gland
BT0521	mammary gland
BT0550	mammary gland
BT0551	mammary gland
BT0570	mammary gland
BT0571	mammary gland
BT0572	mammary gland
BT0575	mammary gland
BT0576	mammary gland
BT0577	mammary gland
BT0578	mammary gland
BT0580	mammary gland
BT0587	mammary gland
BT0589	mammary gland
BT0590	mammary gland
BT0591	mammary gland
BT0594	mammary gland
BT0598	mammary gland
BT0599	mammary gland
BT0610	mammary gland
BT0611	mammary gland
BT0612	mammary gland
BT0613	mammary gland
BT0614	mammary gland
BT0615	mammary gland
BT0617	mammary gland
BT0618	mammary gland
BT0619	mammary gland
BT0620	mammary gland
BT0621	mammary gland
BT0623	mammary gland
BT0624	mammary gland
BT0625	mammary gland
BT0626	mammary gland
BT0627	mammary gland

cDNA Library	Designated Tissue for Library
BT0628	mammary gland
BT0629	mammary gland
BT0630	mammary gland
BT0631	mammary gland
BT0632	mammary gland
BT0633	mammary gland
BT0635	mammary gland
BT0636	mammary gland
BT0637	mammary gland
BT0638	mammary gland
BT0639	mammary gland
BT0640	mammary gland
BT0641	mammary gland
BT0642	mammary gland
BT0643	mammary gland
BT0644	mammary gland
BT0645	mammary gland
BT0646	mammary gland
BT0649	mammary gland
BT0650	mammary gland
BT0651	mammary gland
BT0652	mammary gland
BT0653	mammary gland
BT0654	mammary gland
BT0655	mammary gland
BT0656	mammary gland
BT0657	mammary gland
BT0659	mammary gland
BT0663	mammary gland
BT0664	mammary gland
BT0665	mammary gland
BT0666	mammary gland
BT0667	mammary gland
BT0669	mammary gland
BT0670	mammary gland
BT0671	mammary gland
BT0672	mammary gland
BT0673	mammary gland
BT0675	mammary gland
BT0677	mammary gland

cDNA Library	Designated Tissue for Library
BT0678	mammary gland
BT0679	mammary gland
BT0680	mammary gland
BT0681	mammary gland
BT0682	mammary gland
BT0686	mammary gland
BT0687	mammary gland
BT0688	mammary gland
BT0689	mammary gland
BT0690	mammary gland
BT0691	mammary gland
BT0693	mammary gland
BT0694	mammary gland
BT0695	mammary gland
BT0696	mammary gland
BT0698	mammary gland
BT0700	mammary gland
BT0701	mammary gland
BT0702	mammary gland
BT0703	mammary gland
BT0704	mammary gland
BT0705	mammary gland
BT0707	mammary gland
BT0708	mammary gland
BT0709	mammary gland
BT0710	mammary gland
BT0711	mammary gland
BT0712	mammary gland
BT0713	mammary gland
BT0714	mammary gland
BT0716	mammary gland
BT0717	mammary gland
BT0718	mammary gland
BT0720	mammary gland
BT0721	mammary gland
BT0722	mammary gland
BT0723	mammary gland
BT0724	mammary gland
BT0725	mammary gland
BT0726	mammary gland

cDNA Library	Designated Tissue for Library
BT0727	mammary gland
BT0728	mammary gland
BT0729	mammary gland
BT0730	mammary gland
BT0731	mammary gland
BT0733	mammary gland
BT0734	mammary gland
BT0738	mammary gland
BT0739	mammary gland
BT0740	mammary gland
BT0741	mammary gland
BT0742	mammary gland
BT0744	mammary gland
BT0746	mammary gland
BT0747	mammary gland
BT0749	mammary gland
BT0750	mammary gland
BT0751	mammary gland
BT0752	mammary gland
BT0753	mammary gland
BT0754	mammary gland
BT0755	mammary gland
BT0756	mammary gland
BT0757	mammary gland
BT0758	mammary gland
BT0759	mammary gland
BT0761	mammary gland
BT0762	mammary gland
BT0763	mammary gland
BT0764	mammary gland
BT0765	mammary gland
BT0768	mammary gland
BT0769	mammary gland
BT0771	mammary gland
BT0772	mammary gland
BT0773	mammary gland
BT0774	mammary gland
BT0775	mammary gland
BT0778	mammary gland
BT0779	mammary gland

cDNA Library	Designated Tissue for Library
BT0782	mammary gland
BT0783	mammary gland
BT0785	mammary gland
BT0790	mammary gland
BT0791	mammary gland
BT0798	mammary gland
BT0880	mammary gland
Human Epidermal Keratinocyte Subtraction Library	skin
NCI_CGAP_Sub8	uncharacterized tissue
NCI_CGAP_HN20	head and neck
NCI_CGAP_HN21	head and neck
Human Liver MATCHMAKER cDNA Library(cat.#HL4024AH)	uncharacterized tissue
HT0001	head and neck
HT0002	head and neck
HT0003	head and neck
HT0005	head and neck
HT0006	head and neck
HT0007	head and neck
HT0008	head and neck
HT0011	head and neck
HT0014	head and neck
HT0015	head and neck
HT0016	head and neck
HT0024	head and neck
HT0378	head and neck
HT0379	head and neck
HT0404	thyroid
HT0405	thyroid
HT0406	thyroid
HT0408	thyroid
HT0412	thyroid
HT0424	thyroid
HT0436	thyroid
HT0437	thyroid
HT0446	thyroid
HT0447	thyroid
HT0448	thyroid
HT0457	thyroid
HT0458	thyroid
HT0460	thyroid

cDNA Library	Designated Tissue for Library
HT0461	thyroid
HT0463	thyroid
HT0464	thyroid
HT0471	thyroid
HT0472	thyroid
HT0473	thyroid
HT0474	thyroid
HT0475	thyroid
HT0489	thyroid
HT0490	thyroid
HT0493	thyroid
HT0494	thyroid
HT0495	thyroid
HT0499	thyroid
HT0501	thyroid
HT0502	thyroid
HT0508	thyroid
HT0509	thyroid
HT0511	thyroid
HT0512	thyroid
HT0513	thyroid
HT0514	thyroid
HT0515	thyroid
HT0516	thyroid
HT0517	thyroid
HT0518	thyroid
HT0520	thyroid
HT0521	thyroid
HT0522	thyroid
HT0524	thyroid
HT0525	thyroid
HT0527	thyroid
HT0529	thyroid
HT0530	thyroid
HT0532	thyroid
HT0534	thyroid
HT0535	thyroid
HT0536	thyroid
HT0537	thyroid
HT0538	thyroid

cDNA Library	Designated Tissue for Library
HT0539	thyroid
HT0543	thyroid
HT0544	thyroid
HT0545	thyroid
HT0546	thyroid
HT0547	thyroid
HT0548	thyroid
HT0550	thyroid
HT0551	thyroid
HT0552	thyroid
HT0555	thyroid
HT0556	thyroid
HT0557	thyroid
HT0558	thyroid
HT0559	thyroid
HT0560	thyroid
HT0561	thyroid
HT0562	thyroid
HT0564	thyroid
HT0565	thyroid
HT0567	thyroid
HT0569	thyroid
HT0572	thyroid
HT0573	thyroid
HT0574	thyroid
HT0575	thyroid
HT0577	thyroid
HT0578	thyroid
HT0580	thyroid
HT0581	thyroid
HT0582	thyroid
HT0583	thyroid
HT0584	thyroid
HT0585	thyroid
HT0586	thyroid
HT0587	thyroid
HT0588	thyroid
HT0592	thyroid
HT0593	thyroid
HT0595	thyroid

cDNA Library	Designated Tissue for Library
HT0596	thyroid
HT0597	thyroid
HT0598	thyroid
HT0600	thyroid
HT0603	thyroid
HT0604	thyroid
HT0605	thyroid
HT0606	thyroid
HT0607	thyroid
HT0608	thyroid
HT0609	thyroid
HT0612	thyroid
HT0613	thyroid
HT0615	thyroid
HT0618	thyroid
HT0621	thyroid
HT0622	thyroid
HT0623	thyroid
HT0625	thyroid
HT0626	thyroid
HT0627	head and neck
HT0628	head and neck
HT0629	head and neck
HT0630	head and neck
HT0631	head and neck
HT0632	head and neck
HT0633	head and neck
HT0634	head and neck
HT0635	head and neck
HT0638	head and neck
HT0639	head and neck
HT0641	head and neck
HT0642	head and neck
HT0643	head and neck
HT0644	head and neck
HT0645	head and neck
HT0646	head and neck
HT0647	head and neck
HT0648	head and neck
HT0649	head and neck

cDNA Library	Designated Tissue for Library
HT0650	head and neck
HT0651	head and neck
HT0652	head and neck
HT0653	head and neck
HT0654	head and neck
HT0655	head and neck
HT0657	head and neck
HT0658	head and neck
HT0664	head and neck
HT0668	head and neck
HT0669	head and neck
HT0670	head and neck
HT0671	head and neck
HT0672	head and neck
HT0673	head and neck
HT0674	head and neck
HT0675	head and neck
HT0676	head and neck
HT0677	head and neck
HT0683	head and neck
HT0685	head and neck
HT0686	head and neck
HT0688	head and neck
HT0689	head and neck
HT0691	head and neck
HT0707	head and neck
HT0709	head and neck
HT0729	head and neck
HT0730	head and neck
HT0731	head and neck
HT0738	head and neck
SAGE_SciencePark_MCF7_control_3h	mammary gland
SAGE_SciencePark_MCF7_Control_0h	mammary gland
SAGE_SciencePark_MCF7_estradiol_3h	mammary gland
SAGE_SciencePark_MCF7_estradiol_10h	mammary gland
SAGE_lacZ	mammary gland
SAGE_PTEN	mammary gland
Homo sapiens express Library	uncharacterized tissue
Homo sapiens astrocytoma	uncharacterized tissue
SAGE_95-347	mammary gland

cDNA Library	Designated Tissue for Library
SAGE_95-259	mammary gland
Homo sapiens adult T-cell leukemia cell line JuanaW	uncharacterized tissue
Homo sapiens stomach cancer	uncharacterized tissue
SAGE_95-260	mammary gland
SAGE_95-348	mammary gland
Homo sapiens library (Zhao JR)	uncharacterized tissue
Homo sapiens cartilage	uncharacterized tissue
HEL cell line cDNA library	uncharacterized tissue
Pediatric acute myelogenous leukemia cell (FAB M1) Baylor-HGSC	bone marrow
Pediatric pre-B cell acute lymphoblastic leukemia Baylor-HGSC	lymph node
RACE product	uncharacterized tissue
SAGE_D283	uncharacterized tissue
SAGE_4-HCR	uncharacterized tissue
human bone marrow cDNA library	uncharacterized tissue
Human umbilical vein cord	uncharacterized tissue
HTM1	bone
Differentiation Display RT-PCR of human alveolar macrophage mRNA	uncharacterized tissue
SAGE_Ped_GBM1062	brain
Retinoic acid-induced differential display cDNA library	uncharacterized tissue
suppression subtractive hybridization library SEC1	uncharacterized tissue
estradiol-responsive cDNAs from MCF7 cell line	uncharacterized tissue
Homo sapiens adult T-cell leukemia cell line JuaW	uncharacterized tissue
Homo sapiens HTLV-1 transformed T cell line C91-PL	uncharacterized tissue
human B lymphocyte cDNA library	uncharacterized tissue
NIH_MGC_73	brain
NIH_MGC_74	heart
NIH_MGC_76	liver
NIH_MGC_81	muscle
NIH_MGC_82	testis
Homo sapiens colon cancer	uncharacterized tissue
cDNA from T84 cells	uncharacterized tissue
gastrocarcinoma tissue cDNA library	uncharacterized tissue
Homo sapiens bone marrow	uncharacterized tissue
Homo sapiens fetal cerebral cortex mRNA	uncharacterized tissue
GLA	uncharacterized tissue
GLC	liver
GLD	uncharacterized tissue
NIH_MGC_65	colon
NIH_MGC_67	eye
NIH_MGC_68	lung

cDNA Library	Designated Tissue for Library
NIH_MGC_69	lung
NIH_MGC_70	pancreas
NIH_MGC_72	skin
NIH_MGC_66	ovary
NIH_MGC_71	uterus
colon carcinoma tissue cDNA library	uncharacterized tissue
3.4 (downregulated in larynx carcinoma)	uncharacterized tissue
3.3 (upregulated in larynx carcinoma)	uncharacterized tissue
Homo sapiens library (Heus HC)	uncharacterized tissue
NIH_MGC_78	pancreas
NIH_MGC_83	prostate
NIH_MGC_18	lung
NCI_CGAP_Co26	colon
NCI_CGAP_Co27	colon
NCI_CGAP_Co28	colon
NCI_CGAP_Co29	colon
NIH_MGC_46	uterus
NIH_MGC_48	uncharacterized tissue
NIH_MGC_75	kidney
NIH_MGC_77	lung
NIH_MGC_79	placenta
BT0616	mammary gland
BT0685	mammary gland
BT0736	mammary gland
BT0760	mammary gland
BT0767	mammary gland
BT0770	mammary gland
BT0777	mammary gland
BT0786	mammary gland
BT0789	mammary gland
BT0792	mammary gland
BT0793	mammary gland
BT0794	mammary gland
BT0795	mammary gland
BT0796	mammary gland
BT0800	mammary gland
CT0217	colon
CT0388	colon
CT0389	colon
CT0390	colon

cDNA Library	Designated Tissue for Library
CT0392	colon
CT0393	colon
CT0394	colon
CT0397	colon
CT0398	colon
CT0400	colon
CT0401	colon
CT0402	colon
CT0403	colon
CT0404	colon
CT0405	colon
CT0406	colon
CT0407	colon
CT0408	colon
CT0409	colon
CT0411	colon
CT0412	colon
CT0413	colon
CT0414	colon
CT0415	colon
CT0416	colon
CT0417	colon
CT0425	colon
CT0427	colon
CT0428	colon
CT0429	colon
CT0432	colon
CT0434	colon
CT0470	colon
CT0471	colon
CT0472	colon
CT0473	colon
CT0474	colon
CT0477	colon
CT0483	colon
CT0484	colon
CT0491	colon
CT0492	colon
CT0499	colon
CT0502	colon

cDNA Library	Designated Tissue for Library
HT0020	head and neck
HT0084	head and neck
HT0445	thyroid
HT0496	thyroid
HT0497	thyroid
HT0542	thyroid
HT0571	thyroid
HT0576	thyroid
HT0594	thyroid
HT0619	thyroid
HT0636	head and neck
HT0637	head and neck
HT0640	head and neck
HT0678	head and neck
HT0679	head and neck
HT0696	head and neck
HT0697	head and neck
HT0698	head and neck
HT0699	head and neck
HT0702	head and neck
HT0704	head and neck
HT0705	head and neck
HT0706	head and neck
HT0710	head and neck
HT0711	head and neck
HT0712	head and neck
HT0713	head and neck
HT0714	head and neck
HT0718	head and neck
HT0719	head and neck
HT0720	head and neck
HT0721	head and neck
HT0722	head and neck
HT0723	head and neck
HT0724	head and neck
HT0725	head and neck
HT0726	head and neck
HT0727	head and neck
HT0728	head and neck
HT0734	head and neck

cDNA Library	Designated Tissue for Library
HT0735	head and neck
HT0736	head and neck
HT0737	head and neck
HT0740	head and neck
HT0742	head and neck
HT0743	head and neck
HT0744	head and neck
HT0745	head and neck
HT0746	head and neck
HT0747	head and neck
HT0750	head and neck
HT0751	head and neck
HT0752	head and neck
HT0756	head and neck
HT0757	head and neck
HT0758	head and neck
HT0759	head and neck
HT0760	head and neck
HT0761	head and neck
HT0762	head and neck
HT0764	head and neck
HT0765	head and neck
HT0766	head and neck
HT0767	head and neck
HT0769	head and neck
HT0771	head and neck
HT0773	head and neck
HT0776	head and neck
HT0778	head and neck
HT0779	head and neck
HT0781	head and neck
HT0782	head and neck
HT0783	head and neck
HT0785	head and neck
HT0787	head and neck
HT0788	head and neck
HT0790	head and neck
HT0791	head and neck
HT0792	head and neck
HT0793	head and neck

cDNA Library	Designated Tissue for Library
HT0795	head and neck
HT0796	head and neck
HT0797	head and neck
HT0798	head and neck
HT0799	head and neck
HT0800	head and neck
HT0801	head and neck
HT0802	head and neck
HT0804	head and neck
HT0805	head and neck
HT0806	head and neck
HT0807	head and neck
HT0808	head and neck
HT0809	head and neck
HT0810	head and neck
HT0815	head and neck
HT0819	head and neck
HT0821	head and neck
HT0822	head and neck
HT0824	head and neck
HT0825	head and neck
HT0826	head and neck
HT0827	head and neck
HT0828	head and neck
HT0830	head and neck
HT0837	head and neck
HT0838	head and neck
HT0839	head and neck
HT0840	head and neck
HT0841	head and neck
HT0843	head and neck
HT0845	head and neck
HT0846	head and neck
HT0847	head and neck
HT0850	head and neck
HT0851	head and neck
HT0852	head and neck
HT0854	head and neck
HT0855	head and neck
HT0857	head and neck

cDNA Library	Designated Tissue for Library
HT0858	head and neck
HT0859	head and neck
HT0861	head and neck
HT0862	head and neck
HT0864	head and neck
HT0865	head and neck
HT0868	head and neck
HT0869	head and neck
HT0871	head and neck
HT0872	head and neck
HT0875	head and neck
HT0876	head and neck
HT0877	head and neck
HT0878	head and neck
HT0880	head and neck
HT0881	head and neck
HT0882	head and neck
HT0883	head and neck
HT0885	head and neck
HT0886	head and neck
HT0887	head and neck
HT0893	head and neck
HT0894	head and neck
HT0895	head and neck
HT0905	head and neck
HT0934	head and neck
NN0098	cerebrum
NN0099	cerebrum
NN0101	cerebrum
NN0104	cerebrum
NN0107	cerebrum
NN0110	cerebrum
NN0116	cerebrum
NN0117	cerebrum
NN0118	cerebrum
NN0119	cerebrum
NN0120	cerebrum
NN0127	cerebrum
NN0128	cerebrum
NN0129	cerebrum

cDNA Library	Designated Tissue for Library
NN0130	cerebrum
NN0133	cerebrum
NN0134	cerebrum
NN0135	cerebrum
NN0136	cerebrum
NN0141	cerebrum
NN0142	cerebrum
NN0143	cerebrum
NN0144	cerebrum
NN0145	cerebrum
NN0146	cerebrum
NN0157	cerebrum
NN0165	cerebrum
NN0166	cerebrum
NN0168	cerebrum
NN0169	cerebrum
NN0170	cerebrum
NN0171	cerebrum
NN0172	cerebrum
NN0174	cerebrum
NN0177	cerebrum
NN0178	cerebrum
NN0179	cerebrum
NN0182	cerebrum
NN0183	cerebrum
NN0184	cerebrum
NN0198	cerebrum
NN1035	cerebrum
NN1045	cerebrum
NN1068	cerebrum
NN1075	cerebrum
NN1076	cerebrum
NN1077	cerebrum
NN1078	cerebrum
NN1101	cerebrum
NN1104	cerebrum
NN1105	cerebrum
NN1111	cerebrum
NN1112	cerebrum
NN1120	cerebrum

cDNA Library	Designated Tissue for Library
NN1121	cerebrum
NN1122	cerebrum
NN1123	cerebrum
NN1124	cerebrum
NN1125	cerebrum
NN1126	cerebrum
UT0001	uterus
UT0003	uterus
UT0005	uterus
UT0008	uterus
UT0012	uterus
UT0021	uterus
UT0022	uterus
UT0023	uterus
UT0027	uterus
UT0028	uterus
UT0029	uterus
UT0031	uterus
UT0033	uterus
UT0034	uterus
UT0038	uterus
UT0039	uterus
UT0041	uterus
SAGE_PrCA-1	prostate
SAGE_normal_prostate	prostate
DT0058	uncharacterized tissue
NT0001	brain
NT0008	brain
NT0018	brain
NT0022	brain
NT0023	brain
NT0024	brain
NT0025	brain
NT0042	brain
NT0043	brain
NT0046	brain
NT0048	brain
NT0050	brain
NT0071	brain
NT0074	brain

cDNA Library	Designated Tissue for Library
NT0086	brain
NT0087	brain
NT0092	brain
NT0098	brain
NT0099	brain
NT0100	brain
NT0101	brain
NT0102	brain
NT0103	brain
NT0104	brain
NT0105	brain
NT0109	brain
NT0110	brain
NT0112	brain
NT0113	brain
NT0115	brain
NT0116	brain
NT0117	brain
NT0121	brain
NT0122	brain
NT0123	brain
NT0124	brain
NT0125	brain
NT0126	brain
NT0128	brain
NT0129	brain
NT0837	brain
GN0013	placenta
GN0021	placenta
GN0023	placenta
GN0036	placenta
GN0037	placenta
GN0042	placenta
GN0045	placenta
GN0046	placenta
GN0047	placenta
GN0049	placenta
GN0050	placenta
GN0052	placenta
GN0061	placenta

cDNA Library	Designated Tissue for Library
GN0063	placenta
GN0065	placenta
FT0010	prostate
FT0014	prostate
FT0015	prostate
FT0018	prostate
FT0019	prostate
FT0020	prostate
FT0021	prostate
FT0022	prostate
FT0023	prostate
FT0024	prostate
FT0025	prostate
FT0027	prostate
FT0028	prostate
CT0331	colon
CT0334	colon
CT0336	colon
CT0337	colon
CT0338	colon
CT0339	colon
CT0341	colon
CT0342	colon
CT0343	colon
CT0345	colon
CT0347	colon
CT0348	colon
CT0349	colon
CT0357	colon
CT0358	colon
CT0360	colon
CT0361	colon
CT0363	colon
CT0369	colon
CT0373	colon
CT0376	colon
CT0378	colon
CT0379	colon
CT0380	colon
CT0396	colon

cDNA Library	Designated Tissue for Library
DT0001	uncharacterized tissue
DT0002	uncharacterized tissue
DT0003	uncharacterized tissue
DT0005	uncharacterized tissue
DT0006	uncharacterized tissue
DT0007	uncharacterized tissue
DT0008	uncharacterized tissue
DT0012	uncharacterized tissue
DT0017	uncharacterized tissue
DT0018	uncharacterized tissue
DT0019	uncharacterized tissue
DT0021	uncharacterized tissue
DT0025	uncharacterized tissue
DT0028	uncharacterized tissue
DT0029	uncharacterized tissue
DT0032	uncharacterized tissue
DT0035	uncharacterized tissue
DT0036	uncharacterized tissue
DT0037	uncharacterized tissue
DT0040	uncharacterized tissue
DT0041	uncharacterized tissue
DT0043	uncharacterized tissue
DT0044	uncharacterized tissue
DT0045	uncharacterized tissue
DT0049	uncharacterized tissue
DT0050	uncharacterized tissue
DT0051	uncharacterized tissue
DT0052	uncharacterized tissue
DT0053	uncharacterized tissue
DT0054	uncharacterized tissue
DT0056	uncharacterized tissue
DT0057	uncharacterized tissue
DT0064	uncharacterized tissue
DT0076	uncharacterized tissue
HT0063	head and neck
HT0064	head and neck
HT0068	head and neck
HT0069	head and neck
HT0071	head and neck
HT0072	head and neck

cDNA Library	Designated Tissue for Library
HT0075	head and neck
HT0076	head and neck
HT0077	head and neck
HT0078	head and neck
HT0079	head and neck
HT0080	head and neck
HT0081	head and neck
HT0083	head and neck
HT0085	head and neck
HT0087	head and neck
HT0089	head and neck
HT0092	head and neck
HT0099	head and neck
HT0101	head and neck
HT0103	head and neck
HT0119	head and neck
HT0120	head and neck
HT0121	head and neck
HT0122	head and neck
HT0127	head and neck
HT0129	head and neck
HT0132	head and neck
HT0135	head and neck
HT0142	head and neck
HT0143	head and neck
HT0144	head and neck
HT0146	head and neck
HT0147	head and neck
HT0148	head and neck
HT0149	head and neck
HT0150	head and neck
HT0152	head and neck
HT0153	head and neck
HT0154	head and neck
HT0155	head and neck
HT0156	head and neck
HT0157	head and neck
HT0158	head and neck
HT0159	head and neck
HT0160	head and neck

cDNA Library	Designated Tissue for Library
HT0161	head and neck
HT0162	head and neck
HT0164	head and neck
HT0165	head and neck
HT0166	head and neck
HT0167	head and neck
HT0168	head and neck
HT0169	head and neck
HT0170	head and neck
HT0171	head and neck
HT0174	head and neck
HT0175	head and neck
HT0176	head and neck
HT0177	head and neck
HT0178	head and neck
HT0179	head and neck
HT0180	head and neck
HT0181	head and neck
HT0182	head and neck
HT0183	head and neck
HT0184	head and neck
HT0185	head and neck
HT0186	head and neck
HT0187	head and neck
HT0189	head and neck
HT0190	head and neck
HT0191	head and neck
HT0192	head and neck
HT0193	head and neck
HT0194	head and neck
HT0195	head and neck
HT0197	head and neck
HT0198	head and neck
HT0199	head and neck
HT0200	head and neck
HT0201	head and neck
HT0203	head and neck
HT0204	head and neck
HT0205	head and neck
HT0206	head and neck

cDNA Library	Designated Tissue for Library
HT0207	head and neck
HT0208	head and neck
HT0209	head and neck
HT0214	head and neck
HT0215	head and neck
HT0216	head and neck
HT0217	head and neck
HT0218	head and neck
HT0219	head and neck
HT0220	head and neck
HT0221	head and neck
HT0222	head and neck
HT0223	head and neck
HT0224	head and neck
HT0225	head and neck
HT0226	head and neck
HT0227	head and neck
HT0228	head and neck
HT0229	head and neck
HT0230	head and neck
HT0231	head and neck
HT0232	head and neck
HT0233	head and neck
HT0240	head and neck
HT0241	head and neck
HT0242	head and neck
HT0243	head and neck
HT0244	head and neck
HT0245	head and neck
HT0247	head and neck
HT0248	head and neck
HT0249	head and neck
HT0250	head and neck
HT0251	head and neck
HT0252	head and neck
HT0253	head and neck
HT0254	head and neck
HT0255	head and neck
HT0256	head and neck
HT0257	head and neck

cDNA Library	Designated Tissue for Library
HT0258	head and neck
HT0259	head and neck
HT0260	head and neck
HT0261	head and neck
HT0262	head and neck
HT0263	head and neck
HT0264	head and neck
HT0266	head and neck
HT0267	head and neck
HT0268	head and neck
HT0269	head and neck
HT0270	head and neck
HT0272	head and neck
HT0273	head and neck
HT0274	head and neck
HT0275	head and neck
HT0276	head and neck
HT0277	head and neck
HT0278	head and neck
HT0280	head and neck
HT0281	head and neck
HT0282	head and neck
HT0283	head and neck
HT0285	head and neck
HT0286	head and neck
HT0287	head and neck
HT0289	head and neck
HT0290	head and neck
HT0292	head and neck
HT0293	head and neck
HT0295	head and neck
HT0296	head and neck
HT0297	head and neck
HT0298	head and neck
HT0299	head and neck
HT0300	head and neck
HT0301	head and neck
HT0302	head and neck
HT0303	head and neck
HT0304	head and neck

cDNA Library	Designated Tissue for Library
HT0305	head and neck
HT0309	head and neck
HT0310	head and neck
HT0311	head and neck
HT0312	head and neck
HT0313	head and neck
HT0314	head and neck
HT0315	head and neck
HT0316	head and neck
HT0321	head and neck
HT0322	head and neck
HT0323	head and neck
HT0324	head and neck
HT0325	head and neck
HT0326	head and neck
HT0327	head and neck
HT0328	head and neck
HT0329	head and neck
HT0330	head and neck
HT0331	head and neck
HT0333	head and neck
HT0334	head and neck
HT0335	head and neck
HT0336	head and neck
HT0337	head and neck
HT0338	head and neck
HT0339	head and neck
HT0340	head and neck
HT0342	head and neck
HT0343	head and neck
HT0344	head and neck
HT0345	head and neck
HT0346	head and neck
HT0347	head and neck
HT0348	head and neck
HT0349	head and neck
HT0350	head and neck
HT0351	head and neck
HT0352	head and neck
HT0353	head and neck

cDNA Library	Designated Tissue for Library
HT0354	head and neck
HT0363	head and neck
HT0364	head and neck
HT0366	head and neck
HT0367	head and neck
HT0369	head and neck
HT0370	head and neck
HT0371	head and neck
HT0372	head and neck
HT0373	head and neck
HT0374	head and neck
HT0375	head and neck
HT0376	head and neck
HT0383	head and neck
HT0384	head and neck
HT0386	head and neck
HT0390	head and neck
HT0391	head and neck
HT0392	head and neck
HT0393	head and neck
HT0394	head and neck
HT0395	head and neck
HT0396	head and neck
HT0397	head and neck
HT0400	head and neck
HT0401	thyroid
HT0409	thyroid
HT0410	thyroid
HT0411	thyroid
HT0417	thyroid
HT0418	thyroid
HT0419	thyroid
HT0422	thyroid
HT0425	thyroid
HT0427	thyroid
HT0439	thyroid
HT0441	thyroid
HT0442	thyroid
HT0444	thyroid
HT0449	thyroid

cDNA Library	Designated Tissue for Library
HT0450	thyroid
HT0451	thyroid
HT0452	thyroid
HT0454	thyroid
HT0456	thyroid
LT0001	muscle
LT0002	muscle
LT0003	muscle
LT0007	muscle
LT0008	muscle
LT0009	muscle
LT0011	muscle
LT0016	muscle
LT0017	muscle
LT0018	muscle
LT0021	muscle
LT0022	muscle
LT0023	muscle
LT0025	muscle
LT0027	muscle
LT0030	muscle
LT0031	muscle
LT0032	muscle
LT0042	muscle
LT0045	muscle
LT0057	muscle
LT0058	muscle
LT0069	muscle
LT0074	muscle
LT0075	muscle
PT0001	uncharacterized tissue
PT0002	uncharacterized tissue
PT0003	uncharacterized tissue
PT0004	uncharacterized tissue
PT0005	uncharacterized tissue
PT0006	uncharacterized tissue
PT0007	uncharacterized tissue
PT0013	uncharacterized tissue
PT0014	uncharacterized tissue
PT0015	uncharacterized tissue

cDNA Library	Designated Tissue for Library
PT0016	uncharacterized tissue
PT0019	uncharacterized tissue
PT0023	uncharacterized tissue
PT0025	uncharacterized tissue
PT0026	uncharacterized tissue
PT0027	uncharacterized tissue
PT0028	uncharacterized tissue
PT0030	uncharacterized tissue
PT0031	uncharacterized tissue
PT0047	uncharacterized tissue
PT0048	uncharacterized tissue
PT0049	uncharacterized tissue
PT0050	uncharacterized tissue
ST0016	stomach
ST0063	stomach
ST0088	stomach
ST0089	stomach
ST0090	stomach
ST0091	stomach
ST0096	stomach
ST0098	stomach
ST0110	stomach
ST0111	stomach
ST0112	stomach
ST0113	stomach
ST0116	stomach
ST0117	stomach
ST0118	stomach
ST0119	stomach
ST0121	stomach
ST0122	stomach
ST0124	stomach
ST0125	stomach
ST0129	stomach
ST0130	stomach
ST0131	stomach
ST0132	stomach
ST0133	stomach
ST0134	stomach
ST0136	stomach

cDNA Library	Designated Tissue for Library
ST0137	stomach
ST0138	stomach
ST0139	stomach
ST0142	stomach
ST0144	stomach
ST0145	stomach
ST0150	stomach
ST0152	stomach
ST0156	stomach
ST0157	stomach
ST0158	stomach
ST0159	stomach
ST0160	stomach
ST0162	stomach
ST0163	stomach
ST0164	stomach
ST0165	stomach
ST0166	stomach
ST0168	stomach
ST0169	stomach
ST0170	stomach
ST0171	stomach
ST0172	stomach
ST0173	stomach
ST0174	stomach
ST0175	stomach
ST0176	stomach
ST0177	stomach
ST0178	stomach
ST0179	stomach
ST0180	stomach
ST0181	stomach
ST0182	stomach
ST0183	stomach
ST0184	stomach
ST0185	stomach
ST0186	stomach
ST0188	stomach
ST0189	stomach
ST0190	stomach

cDNA Library	Designated Tissue for Library
ST0191	stomach
ST0192	stomach
ST0194	stomach
ST0196	stomach
ST0197	stomach
ST0198	stomach
ST0199	stomach
ST0200	stomach
ST0201	stomach
ST0203	stomach
ST0204	stomach
ST0208	stomach
ST0209	stomach
ST0212	stomach
ST0213	stomach
ST0214	stomach
ST0215	stomach
ST0216	stomach
ST0218	stomach
ST0220	stomach
ST0222	stomach
ST0224	stomach
ST0225	stomach
ST0227	stomach
ST0228	stomach
ST0230	stomach
ST0231	stomach
ST0232	stomach
ST0233	stomach
ST0234	stomach
ST0235	stomach
ST0236	stomach
ST0240	stomach
ST0241	stomach
ST0242	stomach
ST0244	stomach
ST0246	stomach
ST0256	stomach
ST0258	stomach
ST0259	stomach

cDNA Library	Designated Tissue for Library
ST0260	stomach
ST0261	stomach
ST0262	stomach
ST0263	stomach
ST0264	stomach
ST0265	stomach
ST0266	stomach
ST0268	stomach
ST0272	stomach
ST0273	stomach
ST0274	stomach
ST0275	stomach
ST0276	stomach
ST0277	stomach
ST0279	stomach
ST0280	stomach
ST0281	stomach
ST0282	stomach
ST0283	stomach
ST0284	stomach
ST0285	stomach
ST0286	stomach
ST0287	stomach
ST0288	stomach
ST0292	stomach
ST0293	stomach
ST0297	stomach
ST0299	stomach
ST0300	stomach
ST0301	stomach
ST0302	stomach
ST0303	stomach
ST0307	stomach
ST0308	stomach
ST0311	stomach
ST0314	stomach
ST0315	stomach
ST0317	stomach
TT0002	testis
TT0003	testis

cDNA Library	Designated Tissue for Library
TT0004	testis
TT0005	testis
TT0006	testis
TT0007	testis
TT0008	testis
TT0009	testis
TT0010	testis
TT0011	testis
TT0012	testis
TT0013	testis
TT0014	testis
SAGE_293-CTRL	kidney
Human malignant glioma cell cDNA fragment	uncharacterized tissue
Fetal brain library	brain
Salivary Gland Library	uncharacterized tissue
FLE	uncharacterized tissue
Homo sapiens HaCaT-keratinocytes	uncharacterized tissue
Homo sapiens ATCC HTB-12; SW1088	uncharacterized tissue
Homo sapiens PC-3 prostate tumor	uncharacterized tissue
Neuronal Differentiation of the NT2/D1 cell line.	uncharacterized tissue
fetal human kidney lambda Triplex library	uncharacterized tissue
NCI_CGAP_HN17	head and neck
NCI_CGAP_HN19	head and neck
Homo sapiens PC-3M subline IE8	uncharacterized tissue
Human Fetal Brain cDNA in Uni-ZAP XR Vector	uncharacterized tissue
brain III, cerebral cortex	uncharacterized tissue
brain I, Batten’s subtracted	uncharacterized tissue
Homo sapiens HTLV-1 positive adult T-cell leukemia cell line JuanaW	uncharacterized tissue
Human (activated) smooth muscle cells, DD/RT-PCR products	uncharacterized tissue
SAGE_Chen_LNCaP	prostate
SAGE_Chen_LNCaP_no-DHT	prostate
SAGE_Chen_Normal_Pr	prostate
SAGE_Chen_Tumor_Pr	prostate
Human leukocyte MATCHMAKER cDNA Library	uncharacterized tissue
Human Fetal Brain MATCHMAKER cDNA Library	uncharacterized tissue
Human umbilical vein Endothelial Cell cDNA library	uncharacterized tissue
BN0011	mammary gland
BT0054	mammary gland
BT0272	mammary gland
BT0343	mammary gland

cDNA Library	Designated Tissue for Library
BT0353	mammary gland
BT0354	mammary gland
BT0355	mammary gland
BT0356	mammary gland
BT0373	mammary gland
BT0384	mammary gland
BT0392	mammary gland
BT0501	mammary gland
BT0526	mammary gland
BT0527	mammary gland
BT0528	mammary gland
BT0557	mammary gland
BT0559	mammary gland
BT0561	mammary gland
BT0562	mammary gland
BT0563	mammary gland
BT0592	mammary gland
BT0593	mammary gland
BT0596	mammary gland
BT0597	mammary gland
CN0003	colon
CN0004	colon
CN0005	colon
CN0006	colon
CN0008	colon
CN0010	colon
CN0011	colon
CN0013	colon
CN0017	colon
CN0018	colon
CN0019	colon
CN0031	colon
CN0039	colon
CN0040	colon
CN0043	colon
CN0044	colon
CN0045	colon
CT0258	colon
CT0333	colon
CT0370	colon

cDNA Library	Designated Tissue for Library
CT0381	colon
CT0382	colon
DT0042	uncharacterized tissue
DT0061	uncharacterized tissue
DT0062	uncharacterized tissue
DT0065	uncharacterized tissue
DT0067	uncharacterized tissue
DT0075	uncharacterized tissue
HT0368	head and neck
HT0377	head and neck
HT0407	thyroid
HT0435	thyroid
HT0453	thyroid
HT0470	thyroid
HT0482	thyroid
HT0484	thyroid
LT0004	muscle
LT0056	muscle
LT0059	muscle
LT0061	muscle
LT0063	muscle
LT0065	muscle
LT0066	muscle
LT0067	muscle
LT0070	muscle
LT0094	muscle
PT0009	uncharacterized tissue
PT0033	uncharacterized tissue
PT0043	uncharacterized tissue
PT0046	uncharacterized tissue
ST0205	stomach
ST0247	stomach
ST0278	stomach
ST0290	stomach
Human Pancreatic Islets	pancreatic islet
Homo sapiens MG63	uncharacterized tissue
Homo sapiens U937	uncharacterized tissue
pGEM-T library	uncharacterized tissue
Human pancreas expression library	uncharacterized tissue
Human cDNA Lung Library	uncharacterized tissue

cDNA Library	Designated Tissue for Library
BN0008	mammary gland
BN0025	mammary gland
BN0026	mammary gland
BT0007	mammary gland
BT0357	mammary gland
BT0358	mammary gland
BT0359	mammary gland
BT0360	mammary gland
BT0398	mammary gland
BT0399	mammary gland
BT0400	mammary gland
BT0402	mammary gland
BT0403	mammary gland
BT0407	mammary gland
BT0529	mammary gland
BT0532	mammary gland
BT0564	mammary gland
BT0566	mammary gland
BT0569	mammary gland
BT0583	mammary gland
BT0584	mammary gland
BT0586	mammary gland
BT0595	mammary gland
BT0601	mammary gland
BT0602	mammary gland
BT0603	mammary gland
BT0604	mammary gland
BT0605	mammary gland
BT0606	mammary gland
BT0608	mammary gland
BT0609	mammary gland
CN0007	colon
CN0009	colon
CN0012	colon
CN0014	colon
CN0016	colon
CN0025	colon
CN0026	colon
CN0027	colon
CN0028	colon

cDNA Library	Designated Tissue for Library
CN0029	colon
CN0030	colon
CN0033	colon
CN0041	colon
CN0042	colon
CN0046	colon
CN0048	colon
CN0049	colon
CN0050	colon
CN0051	colon
CN0053	colon
CN0054	colon
CN0056	colon
CT0310	colon
CT0364	colon
CT0365	colon
CT0366	colon
DT0004	uncharacterized tissue
DT0020	uncharacterized tissue
DT0031	uncharacterized tissue
DT0063	uncharacterized tissue
DT0069	uncharacterized tissue
DT0070	uncharacterized tissue
DT0072	uncharacterized tissue
DT0080	uncharacterized tissue
DT0081	uncharacterized tissue
HT0358	head and neck
HT0380	head and neck
HT0398	head and neck
HT0413	thyroid
HT0433	thyroid
HT0443	thyroid
HT0468	thyroid
HT0469	thyroid
HT0481	thyroid
HT0483	thyroid
HT0485	thyroid
HT0486	thyroid
HT0487	thyroid
HT0488	thyroid

cDNA Library	Designated Tissue for Library
HT0491	thyroid
HT0492	thyroid
HT0505	thyroid
HT0506	thyroid
HT0507	thyroid
HT0510	thyroid
HT0528	thyroid
LT0048	muscle
LT0054	muscle
LT0079	muscle
PT0012	uncharacterized tissue
PT0018	uncharacterized tissue
PT0041	uncharacterized tissue
PT0045	uncharacterized tissue
ST0206	stomach
ST0296	stomach
ST0316	stomach
ST0379	stomach
UM0001	uterus
UM0002	uterus
UM0005	uterus
SAGE_CAPAN1	pancreas
SAGE_CAPAN2	pancreas
SAGE_HS766T	pancreas
SAGE_Panc1	pancreas
SAGE_HX	pancreas
SAGE_H126	pancreas
SAGE_Duke_H54_lacZ	brain
SAGE_Duke_H54_EGFRvIII	brain
Placenta Matchmaker cDNA library(Clontech)	uncharacterized tissue
JAR cells, Homo sapiens	uncharacterized tissue
Lupski_dorsal_root_ganglion	nervous
Human rhabdomyosarcoma cDNA	uncharacterized tissue
Sugano cDNA library	uncharacterized tissue
DDRT-PCR	uncharacterized tissue
SAGE_Duke_cerebellum	cerebellum
NIH_MGC_11	placenta
HUVEC Subtracted Library 1	uncharacterized tissue
SAGE_CPDR_LNCaP-C	prostate
SAGE_CPDR_LNCaP-T	prostate

cDNA Library	Designated Tissue for Library
SAGE_293-IND	kidney
Homo sapiens prostate cancer	uncharacterized tissue
Subtracted cDNA libraries from human Jurkat cells	uncharacterized tissue
SAGE_PR317_normal_prostate	prostate
SAGE_PR317_prostate_tumor	prostate
gastric carcinoma cell SGC7901	uncharacterized tissue
Homo sapiens entorhinal cortex adult/elderly	uncharacterized tissue
Clontech HL1008b	uncharacterized tissue
BN0020	mammary gland
BT0197	mammary gland
BT0219	mammary gland
BT0230	mammary gland
BT0363	mammary gland
BT0512	mammary gland
BT0582	mammary gland
BT0585	mammary gland
CN0032	colon
CT0001	colon
CT0006	colon
CT0008	colon
CT0009	colon
CT0010	colon
CT0011	colon
CT0043	colon
Homo sapiens acute myeloid leukemia	uncharacterized tissue
Human cardiac muscle expression library	uncharacterized tissue
BL2	uncharacterized tissue
Human Leukemia K562 Cell Line Express Library	uncharacterized tissue
NCI_CGAP_Br22	mammary gland
pUC-derived chromosome 13 enriched cDNA library	uncharacterized tissue
FIBHBVA	uncharacterized tissue
UM0003	uterus
UM0004	uterus
UM0006	uterus
UM0007	uterus
UM0008	uterus
UM0009	uterus
UM0010	uterus
UM0011	uterus
UM0012	uterus

cDNA Library	Designated Tissue for Library
UM0014	uterus
UM0015	uterus
UM0016	uterus
UM0017	uterus
UM0018	uterus
UM0019	uterus
UM0020	uterus
UM0021	uterus
UM0022	uterus
UM0023	uterus
UM0024	uterus
UM0025	uterus
UM0026	uterus
UM0027	uterus
UM0028	uterus
UM0029	uterus
UM0030	uterus
UM0031	uterus
UM0032	uterus
UM0033	uterus
UM0034	uterus
UM0035	uterus
UM0036	uterus
UM0037	uterus
UM0038	uterus
UM0039	uterus
UM0040	uterus
UM0041	uterus
UM0042	uterus
UM0043	uterus
UM0044	uterus
UM0045	uterus
UM0046	uterus
UM0047	uterus
UM0049	uterus
UM0050	uterus
UM0051	uterus
UM0052	uterus
UM0053	uterus
UM0054	uterus

cDNA Library	Designated Tissue for Library
UM0055	uterus
UM0056	uterus
UM0057	uterus
UM0058	uterus
UM0060	uterus
UM0061	uterus
UM0062	uterus
UM0063	uterus
UM0064	uterus
UM0065	uterus
UM0067	uterus
UM0068	uterus
UM0070	uterus
UM0071	uterus
UM0072	uterus
UM0073	uterus
UM0076	uterus
UM0077	uterus
UM0079	uterus
UM0081	uterus
UM0082	uterus
UM0084	uterus
UM0085	uterus
UM0086	uterus
UM0088	uterus
UM0089	uterus
UM0091	uterus
UM0092	uterus
UM0093	uterus
UM0094	uterus
UM0095	uterus
UM0098	uterus
UM0099	uterus
UM0100	uterus
UM0101	uterus
UM0102	uterus
UM0103	uterus
UM0104	uterus
UM0105	uterus
UM0106	uterus

cDNA Library	Designated Tissue for Library
UM0108	uterus
UM0109	uterus
UM0111	uterus
UM0112	uterus
UM0113	uterus
UM0114	uterus
UM0115	uterus
ST0001	stomach
ST0002	stomach
ST0003	stomach
ST0004	stomach
ST0006	stomach
ST0009	stomach
ST0014	stomach
ST0026	stomach
ST0062	stomach
ST0079	stomach
ST0141	stomach
ST0143	stomach
ST0167	stomach
ST0270	stomach
ST0294	stomach
ST0298	stomach
ST0305	stomach
ST0309	stomach
LT0013	muscle
LT0014	muscle
LT0015	muscle
LT0019	muscle
LT0026	muscle
LT0033	muscle
LT0035	muscle
LT0036	muscle
LT0037	muscle
LT0038	muscle
LT0039	muscle
LT0051	muscle
LT0053	muscle
LT0055	muscle
LT0071	muscle

cDNA Library	Designated Tissue for Library
LT0072	muscle
LT0076	muscle
LT0077	muscle
LT0423	muscle
Homo sapiens cerebrospinal fluid 51 year old female	uncharacterized tissue
CN0002	colon
CN0023	colon
CN0024	colon
CN0035	colon
CN0036	colon
CN0037	colon
CN0038	colon
CN0055	colon
CN0057	colon
NIH_MGC_39	pancreas
NIH_MGC_44	uterus
CT0045	colon
CT0215	colon
CT0216	colon
CT0221	colon
CT0319	colon
CT0350	colon
CT0352	colon
CT0353	colon
CT0354	colon
CT0355	colon
CT0367	colon
CT0383	colon
CT0386	colon
CT0387	colon
SN0004	stomach
SN0005	stomach
SN0007	stomach
SN0009	stomach
SN0010	stomach
SN0011	stomach
SN0012	stomach
SN0013	stomach
SN0014	stomach
SN0015	stomach

cDNA Library	Designated Tissue for Library
SN0016	stomach
SN0017	stomach
SN0018	stomach
SN0019	stomach
SN0020	stomach
SN0021	stomach
SN0022	stomach
SN0023	stomach
SN0024	stomach
SN0027	stomach
SN0028	stomach
SN0029	stomach
SN0030	stomach
SN0031	stomach
SN0032	stomach
SN0033	stomach
FT0032	prostate
FT0037	prostate
FT0038	prostate
FT0039	prostate
FT0040	prostate
FT0041	prostate
FT0042	prostate
FT0044	prostate
FT0045	prostate
FT0046	prostate
FT0049	prostate
FT0050	prostate
FT0051	prostate
FT0052	prostate
FT0053	prostate
FT0054	prostate
FT0055	prostate
FT0056	prostate
FT0057	prostate
FT0058	prostate
FT0059	prostate
FT0060	prostate
FT0061	prostate
FT0062	prostate

cDNA Library	Designated Tissue for Library
FT0067	prostate
FT0068	prostate
FT0069	prostate
FT0070	prostate
FT0071	prostate
FT0073	prostate
FT0074	prostate
FT0077	prostate
FT0079	prostate
FT0080	prostate
FT0082	prostate
FT0083	prostate
FT0089	prostate
FT0093	prostate
FT0095	prostate
FT0096	prostate
FT0097	prostate
FT0099	prostate
FT0100	prostate
FT0101	prostate
FT0103	prostate
FT0104	prostate
FT0111	prostate
FT0115	prostate
FT0116	prostate
FT0117	prostate
FT0119	prostate
FT0122	prostate
FT0123	prostate
FT0126	prostate
FT0128	prostate
FT0129	prostate
FT0130	prostate
FT0131	prostate
FT0133	prostate
FT0134	prostate
FT0144	prostate
FT0145	prostate
FT0148	prostate
FT0149	prostate

cDNA Library	Designated Tissue for Library
FT0150	prostate
FT0151	prostate
FT0152	prostate
FT0153	prostate
FT0157	prostate
FT0159	prostate
FT0160	prostate
FT0162	prostate
FT0163	prostate
FT0164	prostate
FT0166	prostate
FT0167	prostate
FT0169	prostate
FT0170	prostate
FT0179	prostate
FT0180	prostate
FT0181	prostate
FT0182	prostate
PT0151	uncharacterized tissue
AN0001	uncharacterized tissue
AN0003	uncharacterized tissue
AN0004	uncharacterized tissue
AN0005	uncharacterized tissue
AN0006	uncharacterized tissue
AN0007	uncharacterized tissue
AN0012	uncharacterized tissue
AN0013	uncharacterized tissue
AN0014	uncharacterized tissue
AN0016	uncharacterized tissue
AN0017	uncharacterized tissue
AN0024	uncharacterized tissue
AN0025	uncharacterized tissue
AN0026	uncharacterized tissue
AN0027	uncharacterized tissue
AN0029	uncharacterized tissue
AN0032	uncharacterized tissue
AN0033	uncharacterized tissue
AN0034	uncharacterized tissue
AN0038	uncharacterized tissue
AN0040	uncharacterized tissue

cDNA Library	Designated Tissue for Library
AN0041	uncharacterized tissue
AN0042	uncharacterized tissue
AN0047	uncharacterized tissue
AN0048	uncharacterized tissue
AN0056	uncharacterized tissue
AN0058	uncharacterized tissue
AN0059	uncharacterized tissue
AN0060	uncharacterized tissue
AN0061	uncharacterized tissue
AN0062	uncharacterized tissue
AN0075	uncharacterized tissue
AN0078	uncharacterized tissue
AN0079	uncharacterized tissue
AN0081	uncharacterized tissue
AN0088	uncharacterized tissue
AN0094	uncharacterized tissue
BN0098	mammary gland
BN0149	mammary gland
BN0150	mammary gland
BN0177	mammary gland
BN0178	mammary gland
BN0179	mammary gland
BN0188	mammary gland
BN0191	mammary gland
BN0209	mammary gland
BN0210	mammary gland
BN0212	mammary gland
BN0213	mammary gland
BN0214	mammary gland
BN0215	mammary gland
BN0216	mammary gland
BN0227	mammary gland
BN0232	mammary gland
BN0233	mammary gland
BN0234	mammary gland
BN0235	mammary gland
BN0236	mammary gland
BN0237	mammary gland
BN0238	mammary gland
BN0243	mammary gland

cDNA Library	Designated Tissue for Library
BN0245	mammary gland
BN0246	mammary gland
BN0247	mammary gland
BN0248	mammary gland
BN0252	mammary gland
BN0253	mammary gland
BN0254	mammary gland
BN0255	mammary gland
BN0256	mammary gland
BN0257	mammary gland
BN0258	mammary gland
BN0259	mammary gland
BN0260	mammary gland
BN0262	mammary gland
BN0263	mammary gland
BN0264	mammary gland
BN0266	mammary gland
BN0267	mammary gland
BN0268	mammary gland
BN0269	mammary gland
BN0270	mammary gland
BN0271	mammary gland
BN0272	mammary gland
BN0273	mammary gland
BN0274	mammary gland
BN0275	mammary gland
BN0276	mammary gland
BN0277	mammary gland
BN0278	mammary gland
BN0279	mammary gland
BN0280	mammary gland
BN0282	mammary gland
BN0283	mammary gland
BN0284	mammary gland
BN0294	mammary gland
BN0295	mammary gland
BN0296	mammary gland
BN0297	mammary gland
BN0298	mammary gland
BN0299	mammary gland

cDNA Library	Designated Tissue for Library
BN0300	mammary gland
BN0301	mammary gland
BN0302	mammary gland
BN0303	mammary gland
BN0304	mammary gland
BN0305	mammary gland
BN0306	mammary gland
BN0308	mammary gland
BN0309	mammary gland
BN0310	mammary gland
BN0311	mammary gland
BN0312	mammary gland
BN0313	mammary gland
BN0314	mammary gland
BN0316	mammary gland
BN0318	mammary gland
BN0319	mammary gland
BN0320	mammary gland
BN0323	mammary gland
BN0324	mammary gland
BN0325	mammary gland
BN0326	mammary gland
BN0327	mammary gland
BN0328	mammary gland
BN0329	mammary gland
BN0332	mammary gland
BN0333	mammary gland
BN0334	mammary gland
BN0335	mammary gland
BN0348	mammary gland
BN0349	mammary gland
BN0357	mammary gland
BN0358	mammary gland
BN0359	mammary gland
BN0360	mammary gland
BN0361	mammary gland
BN0362	mammary gland
BN0363	mammary gland
SAGE_LN-1	prostate
Human kidney suppression subtractive hybridization	uncharacterized tissue

cDNA Library	Designated Tissue for Library
EN0009	lung
EN0010	lung
EN0018	lung
EN0023	lung
EN0025	lung
EN0026	lung
EN0029	lung
EN0030	lung
EN0031	lung
EN0042	lung
EN0220	lung
ET0005	lung
ET0008	lung
ET0009	lung
ET0011	lung
ET0014	lung
ET0015	lung
ET0016	lung
ET0026	lung
ET0028	lung
ET0029	lung
ET0030	lung
ET0031	lung
ET0037	lung
ET0038	lung
ET0042	lung
ET0043	lung
ET0044	lung
ET0045	lung
ET0046	lung
ET0047	lung
ET0058	lung
ET0060	lung
ET0061	lung
ET0063	lung
ET0066	lung
ET0067	lung
ET0068	lung
ET0070	lung
ET0072	lung

cDNA Library	Designated Tissue for Library
ET0073	lung
ET0074	lung
ET0078	lung
ET0079	lung
ET0080	lung
ET0081	lung
ET0082	lung
ET0084	lung
FN0004	prostate
FN0005	prostate
FN0006	prostate
FN0007	prostate
FN0009	prostate
FN0010	prostate
FN0011	prostate
FN0012	prostate
FN0019	prostate
FN0020	prostate
FN0021	prostate
FN0022	prostate
FN0023	prostate
FN0025	prostate
FN0026	prostate
FN0027	prostate
FN0028	prostate
FN0037	prostate
FN0039	prostate
FN0046	prostate
FN0047	prostate
FN0048	prostate
FN0050	prostate
FN0053	prostate
FN0055	prostate
FN0056	prostate
FN0057	prostate
FN0058	prostate
FN0059	prostate
FN0060	prostate
FN0061	prostate
FN0063	prostate

cDNA Library	Designated Tissue for Library
FN0064	prostate
FN0066	prostate
FN0067	prostate
FN0068	prostate
FN0070	prostate
FN0071	prostate
FN0072	prostate
FN0073	prostate
FN0074	prostate
FN0080	prostate
FN0081	prostate
FN0084	prostate
FN0086	prostate
FN0088	prostate
FN0089	prostate
FN0090	prostate
FN0094	prostate
FN0096	prostate
FN0097	prostate
FN0098	prostate
FN0100	prostate
FN0102	prostate
FN0103	prostate
FN0104	prostate
FN0105	prostate
FN0106	prostate
FN0107	prostate
FN0108	prostate
FN0109	prostate
FN0110	prostate
FN0111	prostate
FN0112	prostate
FN0113	prostate
FN0114	prostate
FN0115	prostate
FN0116	prostate
FN0119	prostate
FN0124	prostate
FN0127	prostate
FN0133	prostate

cDNA Library	Designated Tissue for Library
FN0138	prostate
FN0139	prostate
FN0140	prostate
FN0141	prostate
FN0142	prostate
FN0143	prostate
FN0149	prostate
FN0150	prostate
FN0152	prostate
FN0155	prostate
FN0156	prostate
FN0157	prostate
FN0162	prostate
FN0164	prostate
FN0165	prostate
FN0178	prostate
FN0180	prostate
FN0181	prostate
FN0182	prostate
FN0183	prostate
FN0186	prostate
FN0188	prostate
FN0769	prostate
MT0016	whole blood
MT0017	whole blood
MT0018	whole blood
MT0020	whole blood
MT0022	whole blood
MT0023	whole blood
MT0024	whole blood
MT0025	whole blood
MT0026	whole blood
MT0027	whole blood
MT0028	whole blood
MT0030	whole blood
MT0031	whole blood
MT0032	whole blood
MT0033	whole blood
MT0034	whole blood
MT0035	whole blood

cDNA Library	Designated Tissue for Library
MT0036	whole blood
MT0037	whole blood
MT0038	whole blood
MT0039	whole blood
MT0040	whole blood
MT0042	whole blood
MT0043	whole blood
MT0044	whole blood
MT0049	whole blood
MT0050	whole blood
MT0051	whole blood
MT0052	whole blood
MT0056	whole blood
MT0057	whole blood
MT0058	whole blood
MT0059	whole blood
MT0060	whole blood
MT0061	whole blood
MT0064	whole blood
MT0065	whole blood
MT0067	whole blood
MT0068	whole blood
MT0069	whole blood
MT0070	whole blood
MT0071	whole blood
MT0072	whole blood
MT0074	whole blood
MT0075	whole blood
MT0076	whole blood
MT0077	whole blood
MT0078	whole blood
MT0097	whole blood
MT0098	whole blood
MT0099	whole blood
MT0100	whole blood
MT0101	whole blood
MT0107	whole blood
MT0108	whole blood
MT0109	whole blood
MT0111	whole blood

cDNA Library	Designated Tissue for Library
MT0112	whole blood
MT0113	whole blood
MT0114	whole blood
MT0115	whole blood
MT0116	whole blood
MT0117	whole blood
MT0118	whole blood
MT0119	whole blood
MT0120	whole blood
MT0121	whole blood
MT0124	whole blood
MT0125	whole blood
MT0454	whole blood
OT0034	ovary
SN0006	stomach
SN0008	stomach
SN0041	stomach
SN0054	stomach
SN0057	stomach
SN0076	stomach
SN0077	stomach
SN0078	stomach
SN0080	stomach
TN0001	testis
TN0002	testis
TN0004	testis
TN0008	testis
TN0016	testis
TN0019	testis
TN0024	testis
TN0026	testis
TN0027	testis
TN0028	testis
TN0029	testis
TN0032	testis
TN0033	testis
TN0034	testis
TN0035	testis
TN0036	testis
TN0038	testis

cDNA Library	Designated Tissue for Library
TN0039	testis
TN0040	testis
TN0041	testis
TN0042	testis
TN0043	testis
TN0061	testis
TN0063	testis
TN0064	testis
TN0065	testis
TN0066	testis
TN0067	testis
TN0068	testis
TN0069	testis
TN0070	testis
TN0075	testis
TN0078	testis
TN0079	testis
TN0080	testis
TN0082	testis
TN0093	testis
TN0094	testis
TN0125	testis
TN0165	testis
Homo sapiens skin melanocytic cells	uncharacterized tissue
Homo sapiens HeLa	uncharacterized tissue
GKA	uncharacterized tissue
GKB	liver
GKC	liver
GKD	uncharacterized tissue
SAGE_MESO-12	uncharacterized tissue
Homo sapiens rheumatoid arthritis fibroblast-like synovial cells	uncharacterized tissue
NCI_CGAP_Brn64	pineal gland
NCI_CGAP_Brn65	brain
NCI_CGAP_Brn66	brain
NCI_CGAP_Brn67	brain
NCI_CGAP_Brn70	brain
AN0036	uncharacterized tissue
AN0037	uncharacterized tissue
AN0039	uncharacterized tissue
AN0064	uncharacterized tissue

cDNA Library	Designated Tissue for Library
AN0066	uncharacterized tissue
AN0076	uncharacterized tissue
AN0077	uncharacterized tissue
AN0085	uncharacterized tissue
AN0086	uncharacterized tissue
AN0087	uncharacterized tissue
AN0089	uncharacterized tissue
AN0090	uncharacterized tissue
AN0091	uncharacterized tissue
AN0092	uncharacterized tissue
AN0093	uncharacterized tissue
AN0095	uncharacterized tissue
AN0096	uncharacterized tissue
BN0062	mammary gland
BN0364	mammary gland
BN0365	mammary gland
BT0634	mammary gland
BT0661	mammary gland
BT0674	mammary gland
BT0676	mammary gland
BT06786	mammary gland
BT0684	mammary gland
CT0269	colon
CT0395	colon
CT0451	colon
CT0461	colon
CT0462	colon
CT0463	colon
CT0465	colon
CT0466	colon
CT0467	colon
CT0469	colon
CT0478	colon
CT0479	colon
CT0480	colon
CT0481	colon
CT0482	colon
CT0485	colon
CT0497	colon
CT0500	colon

cDNA Library	Designated Tissue for Library
CT0505	colon
CT0506	colon
CT0507	colon
CT0508	colon
CT0510	colon
CT0511	colon
CT0512	colon
CT0513	colon
CT0516	colon
CT0593	colon
FN0184	prostate
FT0175	prostate
FT0176	prostate
GN0012	placenta
GN0015	placenta
GN0016	placenta
GN0018	placenta
GN0019	placenta
GN0020	placenta
GN0024	placenta
GN0025	placenta
GN0027	placenta
GN0029	placenta
GN0032	placenta
GN0033	placenta
GN0039	placenta
GN0040	placenta
GN0048	placenta
GN0051	placenta
GN0053	placenta
GN0054	placenta
GN0056	placenta
GN0057	placenta
GN0058	placenta
GN0059	placenta
GN0064	placenta
GN0067	placenta
GN0070	placenta
GN0071	placenta
GN0072	placenta

cDNA Library	Designated Tissue for Library
GN0073	placenta
GN0074	placenta
GN0075	placenta
GN0076	placenta
GN0085	placenta
HT0570	thyroid
HT0616	thyroid
HT0617	thyroid
HT0656	head and neck
HT0817	head and neck
HT0860	head and neck
HT0870	head and neck
HT0873	head and neck
HT0874	head and neck
HT0879	head and neck
HT0884	head and neck
HT0888	head and neck
HT0890	head and neck
HT0892	head and neck
HT0900	head and neck
HT0906	head and neck
HT0907	head and neck
HT0911	head and neck
HT0912	head and neck
HT0913	head and neck
HT0914	head and neck
HT0915	head and neck
HT0917	head and neck
HT0918	head and neck
HT0919	head and neck
HT0923	head and neck
HT0926	head and neck
HT0927	head and neck
HT0929	head and neck
HT0931	head and neck
HT0932	head and neck
HT0933	head and neck
HT0935	head and neck
HT0939	head and neck
HT0940	head and neck

cDNA Library	Designated Tissue for Library
HT0943	head and neck
HT0944	head and neck
HT0952	head and neck
MT0104	whole blood
MT0105	whole blood
MT0106	whole blood
MT0110	whole blood
MT0126	whole blood
MT0127	whole blood
NN0042	cerebrum
NN0044	cerebrum
NN0045	cerebrum
NN0131	cerebrum
NN1042	cerebrum
NN1049	cerebrum
NN1128	cerebrum
ST0360	stomach
TN0020	testis
TN0021	testis
TN0023	testis
TN0025	testis
TN0030	testis
TN0037	testis
TN0044	testis
TN0045	testis
TN0046	testis
TN0047	testis
TN0048	testis
TN0049	testis
TN0050	testis
TN0055	testis
TN0060	testis
TN0077	testis
TN0083	testis
TN0084	testis
TN0086	testis
TN0095	testis
TN0096	testis
TN0097	testis
TN0098	testis

cDNA Library	Designated Tissue for Library
TN0108	testis
TN0113	testis
TN0117	testis
TN0118	testis
TN0119	testis
TN0120	testis
TN0121	testis
TN0122	testis
TN0123	testis
TN0124	testis
TN0128	testis
TN0129	testis
TN0130	testis
TN0131	testis
TN0132	testis
TN0133	testis
TN0143	testis
TN0184	testis
UT0002	uterus
UT0004	uterus
UT0007	uterus
UT0009	uterus
UT0010	uterus
UT0011	uterus
UT0018	uterus
UT0019	uterus
UT0032	uterus
UT0036	uterus
UT0040	uterus
UT0050	uterus
UT0056	uterus
UT0058	uterus
UT0059	uterus
UT0060	uterus
UT0061	uterus
UT0062	uterus
UT0063	uterus
UT0064	uterus
UT0065	uterus
UT0068	uterus

cDNA Library	Designated Tissue for Library
UT0069	uterus
UT0071	uterus
UT0072	uterus
UT0074	uterus
UT0082	uterus
ADA	endocrine
ADB	endocrine
ADC	endocrine
Cu	adrenal cortex
DCA	uncharacterized tissue
SAGE_Duke_H306	uncharacterized tissue
Human HO-1 melanoma cells	uncharacterized tissue
Homo sapiens skin adult differential display	uncharacterized tissue
DCB	uncharacterized tissue
GK	uncharacterized tissue
FHTA	uncharacterized tissue
FHTB	uncharacterized tissue
HTB	brain
HTC	brain
HTE	brain
HTF	brain
cdA	endocrine
Human esophageal cancer differentially expressed library	uncharacterized tissue
CB	whole blood
NP1	uncharacterized tissue
NPA	pituitary gland
NPB	uncharacterized tissue
NPC	pituitary gland
NPD	pituitary gland
TP	pituitary gland
AN0063	uncharacterized tissue
AN0067	uncharacterized tissue
AN0069	uncharacterized tissue
AN0082	uncharacterized tissue
AN0083	uncharacterized tissue
AN0084	uncharacterized tissue
BN0376	mammary gland
BN0377	mammary gland
BN0378	mammary gland
BN0379	mammary gland

cDNA Library	Designated Tissue for Library
BN0394	mammary gland
BN0395	mammary gland
BN0396	mammary gland
BN0397	mammary gland
BN0398	mammary gland
BT0812	mammary gland
BT0813	mammary gland
BT0820	mammary gland
BT0821	mammary gland
CI0005	colon
CI0006	colon
CI0010	colon
CI0015	colon
CI0017	colon
CI0018	colon
CS0007	colon
CS0010	colon
CS0011	colon
CS0012	colon
CT0086	colon
CT0437	colon
CT0452	colon
CT0454	colon
CT0456	colon
CT0458	colon
CT0459	colon
CT0486	colon
CT0487	colon
CT0488	colon
CT0514	colon
CT0515	colon
CT0520	colon
CT0522	colon
CT0523	colon
CT0524	colon
CT0527	colon
CT0528	colon
CT0529	colon
CT0531	colon
CT0532	colon

cDNA Library	Designated Tissue for Library
CT0534	colon
GN0014	placenta
GN0017	placenta
GN0030	placenta
GN0031	placenta
GN0034	placenta
GN0035	placenta
GN0038	placenta
GN0055	placenta
GN0077	placenta
GN0079	placenta
GN0080	placenta
GN0081	placenta
GN0083	placenta
GN0084	placenta
GN0087	placenta
GN0089	placenta
GN0090	placenta
GN0091	placenta
GN0092	placenta
HT0540	thyroid
HT0541	thyroid
HT0908	head and neck
HT0909	head and neck
HT0916	head and neck
HT0920	head and neck
HT0921	head and neck
HT0922	head and neck
HT0925	head and neck
HT0930	head and neck
HT0945	head and neck
HT0946	head and neck
HT0947	head and neck
HT0948	head and neck
HT0949	head and neck
HT0955	head and neck
HT0956	head and neck
NN1140	brain
NN1141	brain
SN0079	stomach

cDNA Library	Designated Tissue for Library
TN0022	testis
TN0071	testis
TN0072	testis
TN0073	testis
TN0099	testis
TN0101	testis
TN0103	testis
TN0104	testis
TN0106	testis
TN0107	testis
TN0109	testis
TN0110	testis
TN0111	testis
TN0112	testis
TN0114	testis
TN0115	testis
TN0126	testis
TN0135	testis
TN0136	testis
TN0138	testis
TN0139	testis
TN0140	testis
TN0141	testis
TN0148	testis
TN0149	testis
TN0150	testis
TN0154	testis
TN0155	testis
TN0156	testis
TN0162	testis
UT0006	uterus
UT0042	uterus
UT0043	uterus
UT0044	uterus
UT0045	uterus
UT0046	uterus
UT0047	uterus
UT0052	uterus
UT0053	uterus
UT0073	uterus

cDNA Library	Designated Tissue for Library
UT0075	uterus
UT0076	uterus
UT0077	uterus
UT0078	uterus
UT0087	uterus
UT0091	uterus
Soares_NPBMC	whole blood
BM	bone marrow
MDS	bone marrow
HEMBA1	head and neck
HEMBB1	uncharacterized tissue
MAMMA1	mammary gland
SAGE_Duke_H566	uncharacterized tissue
NT2RM1	nervous
NT2RM2	nervous
NT2RM4	nervous
NT2RP1	nervous
NT2RP2	nervous
NT2RP3	nervous
NT2RP4	nervous
NT2RP5	uncharacterized tissue
OVARC1	ovary
PLACE1	placenta
PLACE2	placenta
PLACE3	placenta
PLACE4	placenta
SKNMC1	uncharacterized tissue
THYRO1	thyroid
VESEN1	uncharacterized tissue
Y79AA1	eye
SAGE_Duke_precrisis_fibroblasts_B	uncharacterized tissue
SAGE_Duke_post_crisis_fibroblasts_B	uncharacterized tissue
NIH_MGC_95	cerebrum
NIH_MGC_96	brain
NIH_MGC_97	testis
SAGE_PERITO-13	soft tissue
HT0950	head and neck
HT0951	head and neck
BN0380	mammary gland
BN0386	mammary gland

cDNA Library	Designated Tissue for Library
BN0387	mammary gland
BN0388	mammary gland
BN0390	mammary gland
BN0406	mammary gland
BN0408	mammary gland
BN0409	mammary gland
BN0410	mammary gland
BN0422	mammary gland
BT0807	mammary gland
BT0811	mammary gland
BT0817	mammary gland
BT0832	mammary gland
BT0833	mammary gland
BT0839	mammary gland
BT0847	mammary gland
BT0848	mammary gland
BT0849	mammary gland
BT0850	mammary gland
BT0851	mammary gland
Homo sapiens BE1	uncharacterized tissue
Myeloma (PCL) cDNA library	whole blood
Myeloma (MYE) cDNA library	bone marrow
Human fetal mesenchymal stem cells library	uncharacterized tissue
Homo sapiens HepG2	uncharacterized tissue
Human H526 cDNA library	uncharacterized tissue
Human HBEC cDNA library	uncharacterized tissue
SAGE_Duke_H275	uncharacterized tissue
Human esophageal carcinoma mRNA	uncharacterized tissue
NIH_MGC_84	adrenal cortex
NIH_MGC_85	lymph node
NIH_MGC_86	bone
NIH_MGC_87	mammary gland
NIH_MGC_88	gastrointestinal tract
human gastric cancer cell line	uncharacterized tissue
CI0002	colon
CI0007	colon
CI0008	colon
CI0019	colon
CI0021	colon
CI0022	colon

cDNA Library	Designated Tissue for Library
CI0028	colon
CI0037	colon
CI0045	colon
CS0005	colon
CS0008	colon
CS0009	colon
CS0017	colon
CS0018	colon
CS0021	colon
CS0022	colon
CS0027	colon
CS0030	colon
CS0066	colon
CT0518	colon
CT0519	colon
CT0521	colon
CT0543	colon
CT0550	colon
CT0554	colon
CT0558	colon
CT0560	colon
CT0563	colon
CT0564	colon
CT0577	colon
CT0581	colon
CT5001	colon
CT5002	colon
CT5003	colon
Homo sapiens adult brain (Stavrides GS)	uncharacterized tissue
Homo sapiens fetal brain (Stavrides GS)	uncharacterized tissue
Homo sapiens Testis (Stavrides GS)	uncharacterized tissue
Homo sapiens adult lung (Stavrides GS)	uncharacterized tissue
Homo sapiens fetal lung (Stavrides GS)	uncharacterized tissue
Homo sapiens fetal liver (Stavrides GS)	uncharacterized tissue
Homo sapiens adult heart (Stavrides GS)	uncharacterized tissue
NIH_MGC_49	skin
NIH_MGC_45	kidney
NIH_MGC_43	eye
NIH_MGC_42	pancreas
HUVEC cDNA Library	uncharacterized tissue

cDNA Library	Designated Tissue for Library
Human H2170 cDNA library	uncharacterized tissue
Human HBECII cDNA library	uncharacterized tissue
AN0030	uncharacterized tissue
AN0068	uncharacterized tissue
BN0347	mammary gland
BN0368	mammary gland
BN0391	mammary gland
BT0814	mammary gland
BT0815	mammary gland
BT0841	mammary gland
CI0013	colon
CS0006	colon
CT0525	colon
CT0555	colon
CT0556	colon
MT0102	whole blood
MT0103	whole blood
MT0122	whole blood
MT0123	whole blood
NN0167	cerebrum
NN1130	cerebrum
NN1142	brain
NN1145	brain
NN1146	brain
NN1147	brain
NN1148	brain
NN1149	brain
NN1175	brain
GN0082	placenta
GN0088	placenta
GN0094	placenta
GN0101	placenta
GN0111	placenta
GN0112	placenta
GN0113	placenta
GN0120	placenta
GN0121	placenta
GN0122	placenta
GN0132	placenta
FT0029	prostate

cDNA Library	Designated Tissue for Library
FT0161	prostate
ST0310	stomach
TN0100	testis
TN0151	testis
TN0152	testis
SAGE_Duke_H1322	uncharacterized tissue
UT0035	uterus
UT0048	uterus
UT0054	uterus
UT0080	uterus
UT0083	uterus
UT0084	uterus
UT0085	uterus
UT0086	uterus
UT0088	uterus
UT0092	uterus
UT0093	uterus
UT0096	uterus
human fetal brain neuroepithelium cDNA library	uncharacterized tissue
Lupski_sciatic_nerve	peripheral nervous system
Lupski_sympathetic_trunk	peripheral nervous system
NIH_MGC_89	kidney
NIH_MGC_90	liver
NIH_MGC_91	prostate
NIH_MGC_92	testis
NIH_MGC_93	genitourinary
SAGE_Duke_H876	uncharacterized tissue
Unstable Angina of AnZhen Hospital	uncharacterized tissue
Homo Sapiens cDNA Library from Peripheral White Blood Cell	uncharacterized tissue
Human Psoriasis Differential Display	uncharacterized tissue
benign human mammary cell line library 123	uncharacterized tissue
Differentially expressed cDNA library of Latent membrane protein 1	uncharacterized tissue
Homo sapiens CD4+ T-cell clone HA1.7	uncharacterized tissue
normal human keratinocytes	uncharacterized tissue
microdissected normal human epidermis	skin
Human subtractive cDNA library	uncharacterized tissue
Homo sapiens library (Park SH)	uncharacterized tissue
Human Lens cDNA (Un-normalized, unamplified): BY	eye
Human Iris cDNA (Un-normalized, unamplified): BX	eye
Human hepatocellular carcinoma subtracted cDNA library	uncharacterized tissue

cDNA Library	Designated Tissue for Library
KN0006	uncharacterized tissue
KT0009	genitourinary
AN0080	uncharacterized tissue
KN0007	uncharacterized tissue
KT0001	genitourinary
KT0002	genitourinary
KT0003	genitourinary
KT0004	genitourinary
KT0005	genitourinary
KT0006	genitourinary
KT0007	genitourinary
KT0008	genitourinary
KT0012	genitourinary
KT0013	genitourinary
KT0014	genitourinary
KT0015	genitourinary
KT0016	genitourinary
KT0017	genitourinary
KT0018	genitourinary
KT0019	genitourinary
KT0023	genitourinary
KT0025	genitourinary
KT0030	genitourinary
KT0031	genitourinary
KT0033	genitourinary
KT0035	genitourinary
KT0036	genitourinary
KT0037	genitourinary
KT0038	genitourinary
KT0039	genitourinary
KT0040	genitourinary
KT0041	genitourinary
KT0042	genitourinary
KT0043	genitourinary
KT0044	genitourinary
KT0045	genitourinary
KT0046	genitourinary
KT0048	genitourinary
KT0050	genitourinary
KT0051	genitourinary

cDNA Library	Designated Tissue for Library
KT0058	genitourinary
KT0059	genitourinary
HB0002	thyroid
HB0004	thyroid
HB0006	thyroid
HB0007	thyroid
HB0017	thyroid
HB0026	thyroid
HB0029	thyroid
HB0031	thyroid
HB0032	thyroid
HB0033	thyroid
HB0034	thyroid
HB0035	thyroid
HB0037	thyroid
HB0038	thyroid
BT0182	mammary gland
BT0808	mammary gland
BT0809	mammary gland
BT0824	mammary gland
BT0826	mammary gland
BT0828	mammary gland
BT0831	mammary gland
BT0834	mammary gland
BT0835	mammary gland
BT0837	mammary gland
BT0838	mammary gland
BT0845	mammary gland
BT0846	mammary gland
BT0854	mammary gland
BT0856	mammary gland
BT0857	mammary gland
BT0858	mammary gland
BT0859	mammary gland
BT0860	mammary gland
BT0861	mammary gland
BT0862	mammary gland
BT0869	mammary gland
BTN0409	mammary gland
BN0381	mammary gland

cDNA Library	Designated Tissue for Library
BN0382	mammary gland
BN0383	mammary gland
BN0384	mammary gland
BN0389	mammary gland
BN0393	mammary gland
BN0401	mammary gland
BN0403	mammary gland
BN0404	mammary gland
BN0405	mammary gland
BN0407	mammary gland
BN0411	mammary gland
BN0412	mammary gland
BN0413	mammary gland
BN0414	mammary gland
BN0415	mammary gland
BN0416	mammary gland
BN0417	mammary gland
BN0418	mammary gland
BN0419	mammary gland
BN0420	mammary gland
BN0421	mammary gland
BN0423	mammary gland
BN0424	mammary gland
BN0425	mammary gland
BN0426	mammary gland
BN0427	mammary gland
BN0428	mammary gland
BN0531	mammary gland
BN0628	mammary gland
CT0109	colon
CT0123	colon
CT0424	colon
CT0453	colon
CT0517	colon
CT0537	colon
CT0538	colon
CT0539	colon
CT0540	colon
CT0541	colon
CT0545	colon

cDNA Library	Designated Tissue for Library
CT0546	colon
CT0547	colon
CT0557	colon
CT0559	colon
CT0561	colon
CT0562	colon
CT0566	colon
CT0570	colon
CT0572	colon
CT0574	colon
CT0583	colon
CT0586	colon
CT0588	colon
CT0589	colon
CT0591	colon
CT0592	colon
CT0594	colon
CT0595	colon
CT0596	colon
CT0597	colon
CT0598	colon
CT0599	colon
CT0600	colon
CT0601	colon
CT0605	colon
CT0606	colon
CT0621	colon
CT0628	colon
CT0634	colon
CT0637	colon
CT0638	colon
CT0641	colon
CT0642	colon
CT0644	colon
CT0648	colon
CT0649	colon
CT0651	colon
CT0652	colon
CT0653	colon
CT0654	colon

cDNA Library	Designated Tissue for Library
CT0685	colon
CT3001	colon
CT3002	colon
CS0015	colon
CS0016	colon
CS0028	colon
CS0031	colon
CS0032	colon
CS0033	colon
CS0034	colon
CS0036	colon
CS0037	colon
CS0038	colon
CS0039	colon
CS0040	colon
CS0041	colon
CS0042	colon
CS0043	colon
CS0044	colon
CS0045	colon
CS0046	colon
CS0047	colon
CS0048	colon
CS0049	colon
CS0050	colon
CS0051	colon
CS0052	colon
CS0068	colon
CS0071	colon
CS0072	colon
CS0073	colon
CI0001	colon
CI0003	colon
CI0012	colon
CI0023	colon
CI0024	colon
CI0029	colon
CI0030	colon
CI0031	colon
CI0032	colon

cDNA Library	Designated Tissue for Library
CI0033	colon
CI0035	colon
CI0040	colon
CI0042	colon
CI0043	colon
CI0044	colon
CI0046	colon
CI0047	colon
CI0048	colon
CI0049	colon
CI0050	colon
CI0051	colon
CI0054	colon
CI0055	colon
CI0056	colon
CI0057	colon
CI0059	colon
CI0060	colon
CI0061	colon
CI0062	colon
CI0063	colon
CI0064	colon
CI0066	colon
CI0075	colon
CI0079	colon
CI0080	colon
CI0081	colon
CI0082	colon
CI0083	colon
CI0084	colon
CI0087	colon
CI0088	colon
CI0089	colon
CI0090	colon
CI0091	colon
CI0092	colon
CI0093	colon
CI0094	colon
CI0095	colon
CI0096	colon

cDNA Library	Designated Tissue for Library
CI0098	colon
CI0099	colon
CI0100	colon
CI0101	colon
CI0102	colon
CI0103	colon
CI0105	colon
CI0107	colon
CI0108	colon
CI0109	colon
CI0110	colon
CI0111	colon
CI0112	colon
CI0113	colon
CI0114	colon
CI0115	colon
CI0116	colon
CI0117	colon
CI0118	colon
CI0119	colon
CI0120	colon
CI0121	colon
CI0122	colon
CI0123	colon
CI0127	colon
CI0128	colon
CI0130	colon
CI0131	colon
CI0132	colon
CI0134	colon
CI0135	colon
CI0136	colon
CI0137	colon
CI0138	colon
CI0139	colon
CI0140	colon
CI0141	colon
CI0142	colon
CI0143	colon
CI0144	colon

cDNA Library	Designated Tissue for Library
CI0145	colon
CI0146	colon
CI0147	colon
CI0148	colon
CI0149	colon
CI0150	colon
CI0151	colon
CI0152	colon
CI0153	colon
CI0154	colon
CI0155	colon
CI0156	colon
CI0157	colon
CI0158	colon
CI0159	colon
CI0160	colon
CI0161	colon
CI0162	colon
CI0163	colon
CI0165	colon
CI0166	colon
CI0167	colon
CI0168	colon
CI0170	colon
CI0172	colon
CI0173	colon
CI0175	colon
CI0177	colon
CI0179	colon
CI0180	colon
CI0181	colon
CI0182	colon
CI0183	colon
CI0184	colon
CI0185	colon
CI0186	colon
CI0189	colon
CI0190	colon
CI0191	colon
CI0193	colon

cDNA Library	Designated Tissue for Library
CI0194	colon
CI0195	colon
CI0196	colon
CI0197	colon
CI0198	colon
CI0199	colon
CI0200	colon
CI0201	colon
CI0415	colon
CI0596	colon
CN0060	colon
CN0061	colon
CN0076	colon
CN0077	colon
CN0078	colon
CN0080	colon
CN0081	colon
CN0082	colon
CN0083	colon
CN0084	colon
CN0085	colon
CN0095	colon
CN0096	colon
CN0097	colon
CN0098	colon
CN0099	colon
CN0104	colon
CN0105	colon
CN0109	colon
CN0110	colon
CN0112	colon
IT0003	genitourinary
IT0004	genitourinary
IT0006	genitourinary
IT0007	genitourinary
IT0008	genitourinary
IT0009	genitourinary
IT0010	genitourinary
IT0011	genitourinary
IT0012	genitourinary

cDNA Library	Designated Tissue for Library
IT0013	genitourinary
IT0014	genitourinary
IT0015	genitourinary
IT0018	genitourinary
IT0019	genitourinary
IT0021	genitourinary
IT0022	genitourinary
IT0023	genitourinary
IT0024	genitourinary
IT0025	genitourinary
IT0026	genitourinary
IT0027	genitourinary
IT0028	genitourinary
IT0029	genitourinary
IT0031	genitourinary
IT0032	genitourinary
IT0033	genitourinary
IT0037	genitourinary
IT0038	genitourinary
IT0039	genitourinary
IT0041	genitourinary
IT0042	genitourinary
IT0043	genitourinary
IT0044	genitourinary
IT0045	genitourinary
IT0046	genitourinary
IT0047	genitourinary
IT0048	genitourinary
HT0043	head and neck
HT01007	head and neck
HT0610	thyroid
HT0924	head and neck
HT0959	head and neck
HT0961	head and neck
HT0962	head and neck
HT0963	head and neck
HT0964	head and neck
HT0965	head and neck
HT0966	head and neck
HT0967	head and neck

cDNA Library	Designated Tissue for Library
HT0968	head and neck
HT0969	head and neck
HT0970	head and neck
HT0973	head and neck
HT0974	head and neck
HT0975	head and neck
HT0976	head and neck
HT0977	head and neck
HT0978	head and neck
HT0979	head and neck
HT0980	head and neck
HT0981	head and neck
HT0982	head and neck
HT0983	head and neck
HT0985	head and neck
HT0986	head and neck
HT0990	head and neck
HT0993	head and neck
HT0994	head and neck
HT1002	head and neck
HT1003	head and neck
HT1007	head and neck
HT1009	head and neck
HT1010	head and neck
HT1011	head and neck
HT1012	head and neck
HT1013	head and neck
HT1014	head and neck
HT1015	head and neck
HT1016	head and neck
HT1017	head and neck
HT1018	head and neck
HT1035	thyroid
HT1036	thyroid
HT1037	thyroid
HT1038	thyroid
HT1048	thyroid
HT1055	thyroid
HT1056	thyroid
HT1057	thyroid

cDNA Library	Designated Tissue for Library
HT1058	thyroid
HT1059	thyroid
HT1060	thyroid
HT1061	thyroid
HT1062	thyroid
HT1064	thyroid
HT1065	thyroid
HT1067	thyroid
HT1068	thyroid
HT1076	thyroid
HT1077	thyroid
HT1078	thyroid
HT1079	thyroid
HT1082	thyroid
HT1085	thyroid
HT1086	thyroid
HT1090	thyroid
HT1092	thyroid
HT1106	thyroid
HT1109	thyroid
HT1110	thyroid
HT1111	thyroid
HT1127	thyroid
HT1136	head and neck
HT1138	head and neck
HT1140	head and neck
HT1145	head and neck
HT1151	head and neck
HT1152	head and neck
HT1183	thyroid
HT1184	thyroid
HT1185	thyroid
HT1188	thyroid
HN0016	head and neck
HN0019	head and neck
HN0020	head and neck
HN0021	head and neck
HN0022	head and neck
HN0023	head and neck
HN0024	head and neck

cDNA Library	Designated Tissue for Library
HN0025	head and neck
HN0027	head and neck
HN0028	head and neck
HN0029	head and neck
HN0030	head and neck
HN0031	head and neck
HN0033	thyroid
HN0034	thyroid
HN0035	pooled tissue
HN0036	pooled tissue
HN0039	thyroid
HN0042	thyroid
HN0043	thyroid
HN0047	thyroid
HN0050	thyroid
HN0052	thyroid
HN0062	thyroid
HN0063	thyroid
HN0065	thyroid
HN0066	thyroid
HN0068	thyroid
HN0069	pooled tissue
HN0070	thyroid
HN0071	thyroid
HN0081	thyroid
HN0082	thyroid
HN0113	thyroid
RT0001	kidney
RT0006	kidney
RT0007	kidney
RT0008	kidney
RT0009	kidney
RT0010	kidney
RT0011	kidney
RT0012	kidney
RT0015	kidney
RT0017	kidney
RT0018	kidney
RT0019	kidney
RT0020	kidney

cDNA Library	Designated Tissue for Library
RT0022	kidney
RT0023	kidney
RT0024	kidney
RT0025	kidney
RT0028	kidney
RT0029	kidney
RT0030	kidney
RT0031	kidney
RT0036	kidney
RT0037	kidney
RT0038	kidney
RT0039	kidney
RT0040	kidney
RT0041	kidney
RT0042	kidney
RT0043	kidney
RT0051	kidney
RT0052	kidney
RT0055	kidney
RT0057	kidney
RT0062	kidney
RT0063	kidney
RT0077	kidney
RT0078	kidney
RT0079	kidney
RT0080	kidney
EN0045	lung
EN0046	lung
EN0047	lung
EN0048	lung
EN0049	lung
EN0051	lung
EN0052	lung
EN0053	lung
EN0054	lung
EN0055	lung
EN0058	lung
EN0059	lung
EN0060	lung
EN0061	lung

cDNA Library	Designated Tissue for Library
EN0063	lung
EN0064	lung
EN0065	lung
EN0066	lung
EN0067	lung
EN0068	lung
EN0071	lung
EN0072	lung
EN0073	lung
EN0074	lung
EN0075	lung
EN0077	lung
EN0078	lung
EN0079	lung
EN0080	lung
EN0081	lung
EN0083	lung
EN0084	lung
EN0085	lung
EN0086	lung
EN0087	lung
EN0088	lung
EN0089	lung
EN0090	lung
EN0091	lung
EN0092	lung
EN0093	lung
EN0094	lung
EN0095	lung
EN0101	lung
EN0102	lung
EN0109	lung
EN0111	lung
EN0112	lung
EN0132	lung
EN0133	lung
EN0194	lung
ET0093	lung
ET0094	lung
ET0095	lung

cDNA Library	Designated Tissue for Library
ET0096	lung
ET0097	lung
ET0098	lung
ET0099	lung
ET0100	lung
ET0101	lung
ET0102	lung
ET0103	lung
ET0104	lung
ET0105	lung
ET0106	lung
ET0107	lung
ET0108	lung
ET0109	lung
ET0110	lung
ET0111	lung
ET0112	lung
ET0113	lung
ET0114	lung
ET0115	lung
ET0116	lung
ET0117	lung
ET0118	lung
ET0119	lung
ET0120	lung
ET0121	lung
ET0122	lung
ET0123	lung
ET0124	lung
ET0125	lung
ET0126	lung
ET0127	lung
ET0129	lung
ET0130	lung
ET0131	lung
ET0132	lung
ET0133	lung
ET0134	lung
ET0135	lung
ET0136	lung

cDNA Library	Designated Tissue for Library
ET0144	lung
ET0145	lung
ET0146	lung
ET0147	lung
ET0148	lung
ET0149	lung
ET0150	lung
ET0151	lung
ET0152	lung
ET0153	lung
ET0154	lung
ET0155	lung
ET0163	lung
ET0164	lung
ET0171	lung
ET0172	lung
ET0173	lung
ET0174	lung
ET0175	lung
ET0176	lung
ET0177	lung
ET0178	lung
ET0180	lung
ET0181	lung
ET0183	lung
ET0184	lung
ET0185	lung
ET0188	lung
ET0191	lung
ET0192	lung
ET0193	lung
ET0195	lung
ET0196	lung
ET0197	lung
ET0198	lung
ET0199	lung
ET0200	lung
ET0201	lung
ET0202	lung
ET0203	lung

cDNA Library	Designated Tissue for Library
ET0205	lung
ET0207	lung
ET0208	lung
ET0209	lung
ET0211	lung
MT0128	whole blood
MT0129	whole blood
MT0130	whole blood
MT0131	whole blood
MT0132	whole blood
MT0133	whole blood
MT0134	whole blood
MT0135	whole blood
MT0136	whole blood
MT0137	whole blood
MT0138	whole blood
MT0139	whole blood
MT0140	whole blood
MT0141	whole blood
MT0142	whole blood
MT0143	whole blood
MT0144	whole blood
MT0146	whole blood
MT0147	whole blood
MT0148	whole blood
MT0149	whole blood
MT0150	whole blood
MT0151	whole blood
MT0152	whole blood
MT0154	whole blood
MT0157	whole blood
MT0158	whole blood
MT0159	whole blood
MT0160	whole blood
MT0161	whole blood
MT0162	whole blood
MT0165	whole blood
MT0166	whole blood
MT0167	whole blood
MT0168	whole blood

cDNA Library	Designated Tissue for Library
MT0170	whole blood
MT0179	whole blood
MT0180	whole blood
MT0181	whole blood
MT0185	whole blood
MT0188	whole blood
MT0189	whole blood
MT0190	whole blood
MT0191	whole blood
MT0192	whole blood
MT0193	whole blood
MT0195	whole blood
MT0198	whole blood
MT0199	whole blood
MT0200	whole blood
MT0201	whole blood
MT0203	whole blood
MT0204	whole blood
MT0205	whole blood
MT0207	whole blood
MT0208	whole blood
MT0209	whole blood
MT0210	whole blood
MT0211	whole blood
MT0212	whole blood
MT0213	whole blood
MT0214	whole blood
MT0215	whole blood
MT0217	whole blood
MT0219	whole blood
MT0220	whole blood
MT0222	whole blood
MT0223	whole blood
MT0224	whole blood
MT0225	whole blood
MT0226	whole blood
MT0227	whole blood
MT0228	whole blood
MT0229	whole blood
MT0231	whole blood

cDNA Library	Designated Tissue for Library
MT0233	whole blood
MT0234	whole blood
MT0235	whole blood
MT0238	whole blood
MT0242	whole blood
MT0244	whole blood
MT0245	whole blood
MT0256	whole blood
MT0259	whole blood
MT0267	whole blood
MT0275	whole blood
MT0281	whole blood
MT0282	whole blood
MT0283	whole blood
MT0285	whole blood
MT0309	whole blood
MT0327	whole blood
MT0328	whole blood
MT0329	whole blood
MT0330	whole blood
NN0155	cerebrum
NN0186	cerebrum
NN0187	cerebrum
NN0189	cerebrum
NN0190	cerebrum
NN0191	cerebrum
NN0193	cerebrum
NN0194	cerebrum
NN0195	cerebrum
NN0196	cerebrum
NN0199	cerebrum
NN0200	cerebrum
NN0203	cerebrum
NN0204	cerebrum
NN0205	cerebrum
NN0206	cerebrum
NN0208	cerebrum
NN0209	cerebrum
NN0210	cerebrum
NN0211	cerebrum

cDNA Library	Designated Tissue for Library
NN0212	cerebrum
NN0213	cerebrum
NN0214	cerebrum
NN0215	cerebrum
NN0216	cerebrum
NN0217	cerebrum
NN0218	cerebrum
NN0219	cerebrum
NN0220	cerebrum
NN0221	brain
NN0222	brain
NN0223	brain
NN0225	brain
NN0226	brain
NN0227	brain
NN0228	brain
NN0229	brain
NN0230	brain
NN0231	brain
NN0232	brain
NN0233	brain
NN0234	brain
NN0237	brain
NN0238	brain
NN0243	brain
NN0244	brain
NN0245	brain
NN0246	brain
NN0247	brain
NN0248	brain
NN0249	brain
NN0250	brain
NN0251	brain
NN1081	cerebrum
NN1152	brain
NN1153	brain
NN1154	brain
NN1155	brain
NN1156	brain
NN1157	brain

cDNA Library	Designated Tissue for Library
NN1158	brain
NN1161	brain
NN1162	brain
NN1163	brain
NN1164	brain
NN1165	brain
NN1169	brain
NN1170	brain
NN1171	brain
NN1172	brain
NN1173	brain
NN1174	brain
NN1176	brain
NN1177	brain
NN1178	brain
NN1179	brain
NN1180	brain
NN1182	brain
NN1183	brain
NN1184	brain
NN1186	brain
NN1187	brain
NN1188	brain
NN1189	brain
NN1191	brain
NN1193	brain
NN1194	brain
NN1195	brain
NN1196	brain
NN1198	brain
NN1199	brain
NN1200	brain
NN1201	brain
NN1202	nervous
NN1203	nervous
NN1204	nervous
NN1205	nervous
NN1207	nervous
NN1209	nervous
NN1210	nervous

cDNA Library	Designated Tissue for Library
NN1211	nervous
NN1216	nervous
NT0131	brain
NT0132	brain
NT0133	brain
NT0135	brain
NT0136	brain
NT0137	brain
NT0138	brain
NT0139	brain
NT0140	brain
NT0141	brain
NT0142	brain
NT0143	brain
NT0144	brain
NT0146	brain
NT0147	brain
NT0148	brain
NT0149	brain
NT0150	brain
NT0151	brain
NT0152	brain
NT0153	brain
NT0154	brain
NT0155	brain
NT0156	brain
NT0157	brain
NT0158	brain
NT0159	brain
NT0162	brain
NT0163	brain
NT0164	brain
NT0167	brain
NT0168	brain
NT0169	brain
NT0170	brain
NT0171	brain
NT0175	brain
NT0176	brain
NT0177	brain

cDNA Library	Designated Tissue for Library
NT0178	brain
NT0179	brain
NT0180	brain
NT0181	brain
NT0182	brain
NT0185	brain
NT0186	brain
NT0187	brain
NT0189	brain
NT0192	brain
NT0193	brain
NT0194	brain
NT0196	brain
NT0197	brain
NT0198	brain
NT0199	brain
NT0200	brain
NT0201	brain
NT0202	brain
NT0203	brain
NT0204	brain
NT0209	brain
NT0210	brain
NT0211	brain
NT0213	brain
NT0214	brain
NT0215	brain
NT0216	brain
NT0217	brain
NT0221	brain
NT0225	brain
NT0226	brain
NT0227	brain
NT0228	brain
NT0229	brain
NT0230	brain
NT0232	brain
NT0235	brain
NT0236	brain
NT0237	brain

cDNA Library	Designated Tissue for Library
NT0239	brain
NT0242	brain
NT0243	brain
NT0244	brain
NT0245	brain
NT0246	brain
NT0247	brain
NT0248	brain
NT0249	brain
NT0250	brain
NT0251	brain
NT0252	brain
NT0256	brain
NT0257	brain
NT0270	brain
NT0271	brain
NT0273	brain
NT0284	brain
NT0289	brain
NT0299	brain
NT1006	brain
OT0123	ovary
GN0026	placenta
GN0066	placenta
GN0068	placenta
GN0093	placenta
GN0097	placenta
GN0099	placenta
GN0100	placenta
GN0102	placenta
GN0103	placenta
GN0104	placenta
GN0107	placenta
GN0108	placenta
GN0109	placenta
GN0110	placenta
GN0123	placenta
GN0126	placenta
GN0127	placenta
GN0131	placenta

cDNA Library	Designated Tissue for Library
GN0133	placenta
GN0134	placenta
GN0135	placenta
GN0136	placenta
GN0137	placenta
GN0138	placenta
GN0139	placenta
GN0140	placenta
GN0141	placenta
GN0143	placenta
GN0144	placenta
GN0149	placenta
GN0150	placenta
GN0151	placenta
GN0152	placenta
GN0153	placenta
GN0154	placenta
GN0157	placenta
GN0158	placenta
GN0160	placenta
GN0161	placenta
GN0162	placenta
GN0163	placenta
GN0164	placenta
GN0165	placenta
GN0166	placenta
GN0167	placenta
GN0171	placenta
GN0172	placenta
GN0173	placenta
GN0176	placenta
GN0177	placenta
GN0178	placenta
GN0179	placenta
GN0180	placenta
GN0182	placenta
GN0183	placenta
GN0184	placenta
GN0185	placenta
GN0186	placenta

cDNA Library	Designated Tissue for Library
GN0187	placenta
GN0188	placenta
GN0189	placenta
GN0190	placenta
GN0191	placenta
GN0192	placenta
GN0193	placenta
GN0194	placenta
GN0195	placenta
GN0196	placenta
GN0197	placenta
GN0198	placenta
GN0199	placenta
GN0200	placenta
GN0201	placenta
GN0202	placenta
GN0203	placenta
GN0204	placenta
GN0205	placenta
GN0206	placenta
GN0207	placenta
GN0208	placenta
GN0209	placenta
GN0210	placenta
GN0211	placenta
GN0212	placenta
GN0213	placenta
GN0214	placenta
GN0215	placenta
GN0216	placenta
GN0217	placenta
GN0220	placenta
GN0221	placenta
GN0227	placenta
GN0229	placenta
GN0230	placenta
GN0231	placenta
GN0235	placenta
GN0236	placenta
GN0238	placenta

cDNA Library	Designated Tissue for Library
GN0239	placenta
GN0240	placenta
GN0241	placenta
GN0246	placenta
GN0247	placenta
GN0248	placenta
GN0249	placenta
GN0250	placenta
GN0253	placenta
GN0257	placenta
GN0264	placenta
GN0268	placenta
GN0269	placenta
GN0270	placenta
GN0271	placenta
GN0272	placenta
GN0273	placenta
GN0274	placenta
GN0276	placenta
GN0280	placenta
GN0283	placenta
GN0284	placenta
GN0287	placenta
GN0290	placenta
GN0291	placenta
GN0296	placenta
GN0297	placenta
GN0298	placenta
GN0300	placenta
GN0301	placenta
GN0302	placenta
GN0303	placenta
GN0304	placenta
GN0305	placenta
GN0306	placenta
GN0307	placenta
GN0309	placenta
GN0310	placenta
GN0311	placenta
GN0314	placenta

cDNA Library	Designated Tissue for Library
GN0315	placenta
GN0317	placenta
GN0319	placenta
GN0320	placenta
GN0321	placenta
GN0323	placenta
GN0324	placenta
GN0326	placenta
GN0327	placenta
GN0332	placenta
GN0333	placenta
GN0335	placenta
GN0336	placenta
GN0340	placenta
GN0344	placenta
GN0353	placenta
GN0354	placenta
GN0356	placenta
GN0357	placenta
GN0359	placenta
GN0361	placenta
GN0362	placenta
GN0363	placenta
GN0364	placenta
GN0366	placenta
GN0380	placenta
GN0385	placenta
GN0392	placenta
GN0393	placenta
GT0691	placenta
FN0082	prostate
FN0193	prostate
FN0194	prostate
FN0195	prostate
FN0197	prostate
FN0198	prostate
FN0200	prostate
FN0201	prostate
FN0202	prostate
FN0203	prostate

cDNA Library	Designated Tissue for Library
FN0204	prostate
FN0210	prostate
FN0211	prostate
FN0212	prostate
FN0213	prostate
FT0183	prostate
FT0184	prostate
FT0190	prostate
FT0191	prostate
FT0192	prostate
FT0193	prostate
FT0194	prostate
FT0195	prostate
FT0199	prostate
FT0200	prostate
FT0201	prostate
FT0202	prostate
FT0203	prostate
FT0204	prostate
FT0205	prostate
FT0207	prostate
FT0208	prostate
FT0209	prostate
FT0210	prostate
FT0211	prostate
FT0212	prostate
TN0105	testis
TN0116	testis
TN0134	testis
TN0142	testis
TN0146	testis
TN0147	testis
TN0164	testis
TN0166	testis
TN0167	testis
TN0168	testis
TN0170	testis
TN0171	testis
TN0173	testis
TN0174	testis

cDNA Library	Designated Tissue for Library
TN0175	testis
TN0176	testis
TN0177	testis
TN0178	testis
TN0179	testis
TN0181	testis
TN0182	testis
TN0196	testis
UT0049	uterus
UT0051	uterus
UT0089	uterus
UT0090	uterus
UT0094	uterus
UT0095	uterus
UT0097	uterus
UT0098	uterus
UT0099	uterus
UT0101	uterus
UT0102	uterus
UT0103	uterus
UT0104	uterus
UT0105	uterus
UT0106	uterus
UT0108	uterus
UT0109	uterus
UT0112	uterus
UT0113	uterus
UT0114	uterus
UT0115	uterus
UT0127	uterus
UT0129	uterus
Homo sapiens paratumor gastric mucosa	uncharacterized tissue
human CD15+ myeloid progenitor cells cDNA Library	uncharacterized tissue
Homo sapiens HeLa (Suzuki,T.)	uncharacterized tissue
NCI_CGAP_Sub9	uncharacterized tissue
Human Retinal Pigment Epithelium cDNA	retina
Melton Human Islets HIZ1	pancreatic islet
SAGE_Meso-12	soft tissue
Saos-2 cDNA library	uncharacterized tissue
4-week-old human embryo cDNA library	uncharacterized tissue

cDNA Library	Designated Tissue for Library
Athersys RAGE Library	uncharacterized tissue
SAGE_Duke_H484	uncharacterized tissue
SAGE_Duke_C609	uncharacterized tissue
NIH_MGC_40	prostate
NIH_MGC_41	skin
LTI_NFL006_PL2	placenta
LTI_FL015_Brn1	brain
LTI_NFL008_TC2	uncharacterized tissue
LTI_NFL010_BC2	uncharacterized tissue
SAGE_Duke_Kidney	kidney
Human THP1 cell line library	uncharacterized tissue
Human carcinoma of esophagus	uncharacterized tissue
Human Keratinocyte Subtraction Library- Downregulated Transcripts	skin
Homo sapiens SiHa	uncharacterized tissue
Homo sapiens atherosclerosis related cDNA library	uncharacterized tissue
NIH_MGC_47	brain
NCI_CGAP_Skn3	skin
NCI_CGAP_Skn4	skin
SAGE_Duke_96-04-P019	brain
Human placenta cDNA expression library	uncharacterized tissue
Human cDNA expression libraries	uncharacterized tissue
Human transformed 16HBE cDNA	uncharacterized tissue
451 (synonym: hlcc1) spinal cord	nervous
Human esophageal carcinoma subtracted cDNA library	uncharacterized tissue
Human esophageal carcinoma cDNA library	uncharacterized tissue
Homo sapiens brain (H Wang)	uncharacterized tissue
FIBHAVI	uncharacterized tissue
Human nasopharynx subtractive cDNA Library	uncharacterized tissue
SAGE_Duke_98-04-P494	cerebellum
Human Genome Express Library	uncharacterized tissue
SKBR3 Lambda Zap Express Library (ATCC#HTB-30)	uncharacterized tissue
Homo sapiens liver fetal (Kishimoto,T.)	uncharacterized tissue
Dimethylfumarate induced differential expression in U937 cells	uncharacterized tissue
human uterine leiomyoma subtractive library	uncharacterized tissue
Sugano Homo sapiens cDNA library	uncharacterized tissue
NIH_MGC_106	uncharacterized tissue
NIH_MGC_113	spleen
SAGE_Duke_H1126	brain
SSH-HCA-D library	uncharacterized tissue
SSH-HCA-U library	uncharacterized tissue

cDNA Library	Designated Tissue for Library
Human lymphocytes	uncharacterized tissue
Human HIV associated B-cell centroblastic lymphoma	uncharacterized tissue
human activated B lymphocyte	uncharacterized tissue
Breast Cells Differential Display ARE-cDNA PCR	uncharacterized tissue
THP-1 Differential Display ARE-cDNA PCR	uncharacterized tissue
Kidney Cells Differential Display ARE-cDNA PCR	uncharacterized tissue
Monocytes Differential Display ARE-cDNA PCR	uncharacterized tissue
SAGE_gastric_cancer_xenograft_X101	stomach
SAGE_Duke_98-05-P608	brain
SAGE_gastric_cancer-G234	stomach
Human rhabdomyosarcoma cDNA Library	uncharacterized tissue
HR85 islet	pancreatic islet
ciliated epithelial cDNA cell library	uncharacterized tissue
SAGE_Duke_leukocyte	whole blood
SAGE_Duke_97-05-P015	brain
SAGE_normal_lung	lung
Homo sapiens differential display (Yong LL)	uncharacterized tissue
SAGE_ependymoblastoma_819	brain
Homo sapiens lung squamous cell carcinoma	uncharacterized tissue
SAGE_breast_myoepithelial	mammary gland
SAGE_breast_hyperplasia	mammary gland
HOA (Human Osteoarthritic Cartilage)	uncharacterized tissue
SAGE_OC14	ovary
NIH_MGC_98	brain
HNC (Human Normal Cartilage)	uncharacterized tissue
Human Fetal cDNA Library	uncharacterized tissue
NIH_MGC_99	uncharacterized tissue
NIH_MGC_100	liver
Homo sapiens hepatocellular carcinoma	uncharacterized tissue
Proliferating Human Erythroid Cells (LCB:ax library)	whole blood
BT0394	uncharacterized tissue
BT0829	uncharacterized tissue
CN0062	uncharacterized tissue
CN0063	uncharacterized tissue
CN0065	colon
CN0067	uncharacterized tissue
CN0068	colon
CN0089	colon
CN0090	uncharacterized tissue
CN0091	uncharacterized tissue

cDNA Library	Designated Tissue for Library
CN0092	uncharacterized tissue
CN0093	uncharacterized tissue
CN0094	uncharacterized tissue
CN0100	uncharacterized tissue
CN0101	uncharacterized tissue
CN0114	uncharacterized tissue
CN0115	uncharacterized tissue
CN0120	uncharacterized tissue
CN0129	uncharacterized tissue
CN0136	uncharacterized tissue
CN0137	uncharacterized tissue
CN0138	uncharacterized tissue
CN0147	colon
CN0150	colon
CN0151	uncharacterized tissue
CN0153	uncharacterized tissue
CN0155	colon
CN0156	uncharacterized tissue
CN0159	uncharacterized tissue
CN0160	uncharacterized tissue
CN0161	uncharacterized tissue
CN0162	uncharacterized tissue
CN0164	uncharacterized tissue
CN0165	uncharacterized tissue
CN0173	uncharacterized tissue
CN0174	uncharacterized tissue
CN0344	uncharacterized tissue
CN0513	uncharacterized tissue
CN0612	uncharacterized tissue
CT0372	uncharacterized tissue
CT0377	uncharacterized tissue
CT0602	uncharacterized tissue
CT0603	uncharacterized tissue
CT0607	uncharacterized tissue
CT0608	uncharacterized tissue
CT0616	uncharacterized tissue
CT0618	uncharacterized tissue
CT0623	uncharacterized tissue
CT0624	uncharacterized tissue
CT0625	uncharacterized tissue

cDNA Library	Designated Tissue for Library
CT0626	uncharacterized tissue
CT0627	uncharacterized tissue
CT0629	colon
CT0631	uncharacterized tissue
CT0635	uncharacterized tissue
CT0639	uncharacterized tissue
CT0640	uncharacterized tissue
CT0643	uncharacterized tissue
CT0645	uncharacterized tissue
CT0655	uncharacterized tissue
CT0656	colon
CT0657	uncharacterized tissue
CT0658	colon
CT0659	uncharacterized tissue
CT0660	uncharacterized tissue
CT0661	uncharacterized tissue
CT0662	uncharacterized tissue
CT0663	uncharacterized tissue
CT0664	uncharacterized tissue
CT0669	uncharacterized tissue
CT0674	colon
CT0676	uncharacterized tissue
CT0683	uncharacterized tissue
CT0691	uncharacterized tissue
CT0692	uncharacterized tissue
CT0695	uncharacterized tissue
CT0697	uncharacterized tissue
CT0709	uncharacterized tissue
CT0710	uncharacterized tissue
CT0711	uncharacterized tissue
CT0714	uncharacterized tissue
CT0715	uncharacterized tissue
CT0716	uncharacterized tissue
CT0730	uncharacterized tissue
CT0731	uncharacterized tissue
CT0732	uncharacterized tissue
CT0734	uncharacterized tissue
CT0735	uncharacterized tissue
CT0736	uncharacterized tissue
CT0737	uncharacterized tissue

cDNA Library	Designated Tissue for Library
CT0741	uncharacterized tissue
CT0743	uncharacterized tissue
CT0744	uncharacterized tissue
CT0748	uncharacterized tissue
CT0749	uncharacterized tissue
CT0753	uncharacterized tissue
CT0754	uncharacterized tissue
CT0772	uncharacterized tissue
CT0779	uncharacterized tissue
CT0780	uncharacterized tissue
CT0781	uncharacterized tissue
CT0782	uncharacterized tissue
CT0783	uncharacterized tissue
CT0793	uncharacterized tissue
CT0794	uncharacterized tissue
CT0795	uncharacterized tissue
CT0798	uncharacterized tissue
CT0799	uncharacterized tissue
CT0800	uncharacterized tissue
CT0801	colon
CT0802	colon
CT0803	uncharacterized tissue
CT0804	colon
CT0805	uncharacterized tissue
CT0806	colon
CT0807	uncharacterized tissue
CT0808	uncharacterized tissue
CT0809	uncharacterized tissue
CT0810	uncharacterized tissue
CT0811	uncharacterized tissue
CT0813	uncharacterized tissue
CT0814	uncharacterized tissue
CT0815	uncharacterized tissue
CT0817	uncharacterized tissue
CT0818	uncharacterized tissue
CT0819	uncharacterized tissue
CT0824	uncharacterized tissue
EN0096	lung
EN0097	uncharacterized tissue
EN0098	lung

cDNA Library	Designated Tissue for Library
EN0100	uncharacterized tissue
ET0138	lung
ET0139	uncharacterized tissue
ET0140	lung
ET0141	uncharacterized tissue
ET0142	uncharacterized tissue
ET0206	uncharacterized tissue
ET0226	uncharacterized tissue
ET0227	uncharacterized tissue
ET0237	uncharacterized tissue
ET0245	uncharacterized tissue
ET0251	uncharacterized tissue
ET0254	uncharacterized tissue
ET0255	uncharacterized tissue
ET0256	lung
ET0257	uncharacterized tissue
ET0258	uncharacterized tissue
ET0259	uncharacterized tissue
ET0260	uncharacterized tissue
ET0262	lung
ET0266	uncharacterized tissue
ET0268	lung
ET0272	lung
ET0273	uncharacterized tissue
ET0274	uncharacterized tissue
ET0277	lung
ET0675	uncharacterized tissue
FN0206	prostate
FN0209	prostate
FN0214	uncharacterized tissue
FT0186	uncharacterized tissue
FT0206	uncharacterized tissue
GN0170	uncharacterized tissue
GN0175	placenta
GN0233	uncharacterized tissue
GN0275	uncharacterized tissue
GN0277	uncharacterized tissue
GN0281	uncharacterized tissue
GN0282	uncharacterized tissue
GN0288	uncharacterized tissue

cDNA Library	Designated Tissue for Library
GN0289	uncharacterized tissue
GN0294	placenta
GN0295	uncharacterized tissue
GN0313	uncharacterized tissue
GN0328	uncharacterized tissue
GN0329	uncharacterized tissue
GN0330	uncharacterized tissue
GN0334	uncharacterized tissue
GN0337	uncharacterized tissue
GN0338	uncharacterized tissue
GN0339	uncharacterized tissue
GN0345	uncharacterized tissue
GN0348	uncharacterized tissue
GN0349	uncharacterized tissue
GN0351	uncharacterized tissue
GN0365	uncharacterized tissue
GN0367	uncharacterized tissue
GN0374	uncharacterized tissue
GN0375	placenta
GN0376	uncharacterized tissue
GN0405	uncharacterized tissue
GN0406	uncharacterized tissue
GN0407	uncharacterized tissue
GN0408	uncharacterized tissue
GN0410	uncharacterized tissue
GN0411	uncharacterized tissue
GN0412	uncharacterized tissue
GN0428	uncharacterized tissue
GN0429	uncharacterized tissue
GN0430	uncharacterized tissue
GN0431	uncharacterized tissue
GN0432	uncharacterized tissue
GN0434	uncharacterized tissue
GN0437	uncharacterized tissue
GN0446	uncharacterized tissue
GN0461	uncharacterized tissue
GN0468	uncharacterized tissue
GN0470	uncharacterized tissue
GN0471	uncharacterized tissue
GN0473	uncharacterized tissue

cDNA Library	Designated Tissue for Library
GN0474	uncharacterized tissue
GN0486	uncharacterized tissue
GN0488	uncharacterized tissue
GN0493	uncharacterized tissue
GN0494	uncharacterized tissue
GN0495	uncharacterized tissue
GN0496	uncharacterized tissue
GN0497	placenta
GN0498	uncharacterized tissue
GN0499	uncharacterized tissue
GN0500	uncharacterized tissue
GN0501	uncharacterized tissue
GN0502	uncharacterized tissue
GN0506	uncharacterized tissue
GN0511	placenta
GN0513	placenta
GN0516	placenta
HN0045	thyroid
HN0046	thyroid
HN0049	thyroid
HN0053	thyroid
HN0054	pooled tissue
HN0055	thyroid
HN0056	thyroid
HN0057	thyroid
HN0058	thyroid
HN0060	thyroid
HN0061	thyroid
HN0073	pooled tissue
HN0074	thyroid
HN0075	thyroid
HN0076	pooled tissue
HN0077	thyroid
HN0078	pooled tissue
HN0079	pooled tissue
HN0083	thyroid
HN0114	thyroid
HN0123	thyroid
HN0124	thyroid
HN0125	thyroid

cDNA Library	Designated Tissue for Library
HN0126	thyroid
HN0127	thyroid
HN0132	thyroid
HN0135	thyroid
HN0145	thyroid
HN0148	thyroid
HN0149	thyroid
HN0150	pooled tissue
HN0151	thyroid
HN0152	thyroid
HN0153	thyroid
HN0157	thyroid
HN0158	thyroid
HN0159	thyroid
HN0160	thyroid
HN0161	thyroid
HN0163	thyroid
HN0164	thyroid
HN0166	thyroid
HN0175	thyroid
HN0177	thyroid
HN0179	thyroid
HN0183	thyroid
HN0184	thyroid
HN0185	thyroid
HN0188	thyroid
HN0190	thyroid
HN0203	thyroid
HN0205	thyroid
HN0206	thyroid
HN0209	thyroid
HT0972	head and neck
HT0996	head and neck
HT0999	head and neck
HT1032	thyroid
HT1033	thyroid
HT1039	thyroid
HT1050	thyroid
HT1051	thyroid
HT1052	thyroid

cDNA Library	Designated Tissue for Library
HT1075	thyroid
HT1087	thyroid
HT1088	thyroid
HT1091	thyroid
HT1093	thyroid
HT1100	thyroid
HT1104	thyroid
HT1132	head and neck
HT1141	head and neck
HT1144	head and neck
HT1146	head and neck
HT1150	head and neck
HT1155	head and neck
HT1160	head and neck
HT1161	head and neck
HT1162	head and neck
HT1163	head and neck
HT1165	head and neck
HT1166	head and neck
HT1167	head and neck
HT1168	head and neck
HT1169	head and neck
HT1170	head and neck
HT1171	head and neck
HT1173	thyroid
HT1174	thyroid
HT1176	thyroid
HT1189	head and neck
HT1190	head and neck
HT1191	head and neck
HT1192	head and neck
HT1193	head and neck
HT1194	head and neck
HT1195	head and neck
HT1197	head and neck
HT1198	head and neck
HT1204	head and neck
HT1205	head and neck
HT1207	head and neck
HT1208	head and neck

cDNA Library	Designated Tissue for Library
HT1209	head and neck
HT1227	head and neck
HT1232	thyroid
HT1233	thyroid
HT1235	thyroid
HT1241	thyroid
HT1246	thyroid
HT1247	thyroid
HT1248	thyroid
HT1249	thyroid
HT1251	thyroid
HT1252	thyroid
HT1253	thyroid
HT1255	thyroid
HT1256	thyroid
HT1258	thyroid
HT1263	thyroid
HT1265	thyroid
HT1267	thyroid
HT1268	thyroid
HT1269	thyroid
HT1270	thyroid
HT1273	thyroid
HT1277	thyroid
HT1284	thyroid
HT1294	thyroid
HT1295	thyroid
HT1296	thyroid
HT1297	thyroid
HT1298	thyroid
HT1301	thyroid
HT1302	thyroid
HT1306	thyroid
HT1311	thyroid
HT1312	thyroid
HT1313	thyroid
KN0008	uncharacterized tissue
KN0014	uncharacterized tissue
KN0030	uncharacterized tissue
KN0031	uncharacterized tissue

cDNA Library	Designated Tissue for Library
KN0033	uncharacterized tissue
KN0034	uncharacterized tissue
KN0036	uncharacterized tissue
LT0005	uncharacterized tissue
MT0182	whole blood
MT0186	whole blood
MT0187	whole blood
MT0196	whole blood
MT0202	whole blood
MT0218	whole blood
MT0236	whole blood
MT0237	whole blood
MT0246	whole blood
MT0247	whole blood
MT0248	whole blood
MT0255	whole blood
MT0260	whole blood
MT0261	whole blood
MT0262	whole blood
MT0263	whole blood
MT0264	whole blood
MT0265	whole blood
MT0266	whole blood
MT0270	whole blood
MT0280	whole blood
MT0286	whole blood
MT0287	whole blood
MT0288	whole blood
MT0289	whole blood
MT0290	whole blood
MT0291	whole blood
MT0292	whole blood
MT0293	whole blood
MT0294	whole blood
MT0295	whole blood
MT0296	whole blood
MT0297	whole blood
MT0298	whole blood
MT0311	whole blood
MT0316	whole blood

cDNA Library	Designated Tissue for Library
MT0331	whole blood
MT0332	whole blood
MT0336	whole blood
MT0337	whole blood
MT0338	whole blood
MT0339	whole blood
MT0340	whole blood
MT0343	whole blood
MT0344	whole blood
MT0345	whole blood
MT0346	whole blood
MT0347	whole blood
MT0348	whole blood
MT0349	whole blood
MT0350	whole blood
MT0351	whole blood
MT0352	whole blood
MT0353	whole blood
MT0354	whole blood
MT0355	whole blood
MT0356	whole blood
MT0357	whole blood
MT0361	whole blood
MT0364	whole blood
MT0365	whole blood
MT0374	whole blood
MT0388	whole blood
MT0390	whole blood
MT0393	whole blood
MT0394	whole blood
MT0395	whole blood
MT0400	whole blood
MT0417	whole blood
MT0420	whole blood
MT0421	whole blood
MT0427	whole blood
MT0428	whole blood
MT0433	whole blood
NN0188	cerebrum
NN0281	uncharacterized tissue

cDNA Library	Designated Tissue for Library
NN1197	brain
NN2003	nervous
NN2004	brain
NN2006	uncharacterized tissue
NT0219	brain
NT0224	brain
NT0233	brain
NT0261	brain
NT0263	brain
NT0264	brain
NT0265	brain
NT0266	brain
NT0267	brain
NT0268	brain
NT0269	brain
NT0272	brain
NT0274	brain
NT0278	brain
NT0280	brain
NT0282	brain
NT0283	brain
NT0285	brain
NT0286	brain
NT0287	brain
NT0288	brain
NT0290	brain
NT0291	brain
NT0294	brain
NT0314	uncharacterized tissue
NT0319	uncharacterized tissue
NT0911	uncharacterized tissue
OT0098	uncharacterized tissue
OT0099	uncharacterized tissue
OT0101	uncharacterized tissue
OT0105	uncharacterized tissue
OT0106	uncharacterized tissue
OT0109	uncharacterized tissue
OT0127	uncharacterized tissue
OT0129	uncharacterized tissue
OT0144	uncharacterized tissue

cDNA Library	Designated Tissue for Library
OT0146	uncharacterized tissue
OT0159	uncharacterized tissue
OT0161	uncharacterized tissue
OT0166	uncharacterized tissue
OT0167	uncharacterized tissue
OT0177	uncharacterized tissue
OT0180	uncharacterized tissue
OT0185	uncharacterized tissue
OT0186	uncharacterized tissue
OT0187	uncharacterized tissue
OT0188	uncharacterized tissue
OT0190	uncharacterized tissue
OT0191	uncharacterized tissue
OT0196	uncharacterized tissue
OT0197	uncharacterized tissue
OT0200	uncharacterized tissue
OT0201	uncharacterized tissue
OT0202	uncharacterized tissue
OT0203	uncharacterized tissue
OT0204	uncharacterized tissue
OT0205	uncharacterized tissue
OT0207	uncharacterized tissue
OT0211	uncharacterized tissue
OT0213	uncharacterized tissue
OT0214	uncharacterized tissue
OT0215	uncharacterized tissue
OT0216	uncharacterized tissue
OT0219	uncharacterized tissue
RT0026	kidney
RT0033	kidney
RT0035	uncharacterized tissue
RT0044	uncharacterized tissue
RT0045	kidney
RT0048	kidney
RT0053	uncharacterized tissue
RT0054	kidney
RT0056	uncharacterized tissue
RT0058	uncharacterized tissue
RT0061	uncharacterized tissue
RT0064	uncharacterized tissue

cDNA Library	Designated Tissue for Library
RT0066	uncharacterized tissue
RT0067	uncharacterized tissue
RT0069	uncharacterized tissue
RT0070	uncharacterized tissue
RT0072	uncharacterized tissue
SN0349	uncharacterized tissue
UT0116	uterus
UT0117	uterus
UT0118	uncharacterized tissue
UT0119	uterus
SAGE_normal_gastric_body_epithelial	stomach
SAGE_98-04-P117	uncharacterized tissue
HB0003	uncharacterized tissue
SAGE_normal_pediatric_cortex_H1571	cerebrum
NIH_MGC_114	brain
NIH_MGC_115	pooled tissue
NIH_MGC_116	pooled tissue
NIH_MGC_118	whole blood
NIH_MGC_119	brain
NIH_MGC_120	pooled tissue
NIH_MGC_121	brain
NIH_MGC_122	pooled tissue
NIH_MGC_102	salivary gland
SAGE_normal_liver	liver
SAGE_98-05-P040	uncharacterized tissue
SAGE_ependymoma_1394	brain
SAGE_98-13-P301	uncharacterized tissue
cDNA library of human CD 34+ stem/progenitor cells	uncharacterized tissue
Homo sapiens breast cancer	uncharacterized tissue
Homo sapiens library (Geerts D)	uncharacterized tissue
Homo sapiens fetal bone marrow	uncharacterized tissue
Human Cultured Dermal Papilla Cell cDNA Library	uncharacterized tissue
Homo sapiens T-cell library (Sugita Y)	uncharacterized tissue
Homo sapiens E-cell library (Sugita Y)	uncharacterized tissue
Homo sapiens Mast cell library (Sugita Y)	uncharacterized tissue
Homo sapiens monocyte library (Sugita Y)	uncharacterized tissue
Human cell line A431 subclone overexpressing hsp27	uncharacterized tissue
SAGE_H972	uncharacterized tissue
k562 cell cDNA Library	uncharacterized tissue
SAGE_glioma_1150	brain

cDNA Library	Designated Tissue for Library
SAGE_97-05-P312	uncharacterized tissue
human HNSCC Library	uncharacterized tissue
Human abdominal aortic adventitia from an aneurysm specimen	uncharacterized tissue
cDNA Library from human CD34+ stem/progenitor cells	uncharacterized tissue
313 (synonym: hlcc2)	uncharacterized tissue
686 (synonym: hlcc3)	muscle
HM3/S3	muscle
Human endometrium	uncharacterized tissue
SAGE_DL5	uncharacterized tissue
SAGE_NORMAL_HEART	uncharacterized tissue
SAGE_DL7	uncharacterized tissue
SAGE_H1413	uncharacterized tissue
NIH_MGC_110	mammary gland
NIH_MGC_111	uncharacterized tissue
NIH_MGC_112	skin
Human insulinoma	pancreatic islet
Human Retinal Pigment Epithelium (2)	retina
Homo sapiens breast cancer-related	uncharacterized tissue
Human leukaemia cell U937 cDNA	uncharacterized tissue
SAGE_98-09-P558	uncharacterized tissue
SAGE_ependymoma239	brain
Human fetal brain cDNA library	uncharacterized tissue
Homo sapiens stomach	uncharacterized tissue
Dermal Papillae cDNA Library from alopecia areata patient	uncharacterized tissue
Human SPRL genes	uncharacterized tissue
SAGE_Hemangioma_146	skin
SAGE_Universal_Reference_Human_RNA	pooled tissue
NIH_MGC_108	kidney
SAGE_H127	brain
Human fetal kidney subtracted cDNA library	uncharacterized tissue
Homo sapiens fetal bone marrow mesenchymal stem cells	uncharacterized tissue
SAGE_ependymoma_353	brain
Human cancer	uncharacterized tissue
Human normal tissue	uncharacterized tissue
suppression subtractive hybridization library of human cancer	uncharacterized tissue
Homo sapiens SH-SY5Y	uncharacterized tissue
Melton Normalized Human Islet 4 N4-HIS 1	pancreatic islet
Homo sapiens astrocytoma grade 2	uncharacterized tissue
SAGE_DCIS-3	mammary gland
SAGE_DCIS-4	mammary gland

cDNA Library	Designated Tissue for Library
SAGE_DCIS-5	mammary gland
SAGE_IDC-3	mammary gland
SAGE_IDC-4	mammary gland
SAGE_IDC-5	mammary gland
Homo sapiens gastric cancer cell line BGC823	uncharacterized tissue
SAGE_H408	uncharacterized tissue
Plasmid subtractive library of human umbilical vein endothelial cells	uncharacterized tissue
SAGE_gastric_cancer-G189	gastrointestinal tract
SAGE_ependymoma_455	brain
SAGE_ependymoma_582	brain
DDRT-PCR (Li D)	uncharacterized tissue
SAGE_normal_spinal_cord	nervous
SAGE_astrocytoma_H388	uncharacterized tissue
Homo sapiens multiple myeloma	uncharacterized tissue
FIBIP	uncharacterized tissue
NIH_MGC_124	cerebrum
NIH_MGC_125	ovary
SAGE_astrocytoma_H154	uncharacterized tissue
SAGE_ependymoma_510	uncharacterized tissue
SAGE_ependymoma_512	uncharacterized tissue
SAGE_Normal_Human_Bone_Marrow	uncharacterized tissue
Human K562 cells	uncharacterized tissue
NIH_MGC_101	lung
SAGE_Astrocytoma_H272	uncharacterized tissue
SAGE_ependymoma_580	uncharacterized tissue
Human Fetal Pancreas 1A	pancreas
Human Fetal Pancreas 1B	pancreas
SAGE_Astrocytoma_H970	uncharacterized tissue
Human fetal hippocampus cDNA library	uncharacterized tissue
adrenal gland (DD-RT-PCR vs PHEO)	uncharacterized tissue
PHEO (DD-RT-PCR vs adrenal gland)	uncharacterized tissue
Homo sapiens S284N	uncharacterized tissue
Homo sapiens S284T	uncharacterized tissue
Homo sapiens S285T	uncharacterized tissue
Homo sapiens pla-801c	uncharacterized tissue
SAGE_normal_heart	heart
NIH_MGC_126	pooled tissue
Homo sapiens 1F6	uncharacterized tissue
NIH_MGC_127	pooled tissue
NIH_MGC_128	pooled tissue

cDNA Library	Designated Tissue for Library
cDNA subtractive library of human rectum adencarcinoma	uncharacterized tissue
Homo sapiens adipose tissue	uncharacterized tissue
PN001	uncharacterized tissue
SAGE-gastric-normal-antrum	uncharacterized tissue
SAGE-gastric-cancer-X43	uncharacterized tissue
Expressed sequences from YACs	uncharacterized tissue
SAGE_Oligodendroglioma_bulk_1001	uncharacterized tissue
Human cutaneous subtractive library	uncharacterized tissue
Homo sapiens ATL-derived JuanaW	uncharacterized tissue
Homo sapiens HTLV-1-transformed C91-PL	uncharacterized tissue
NCI_CGAP_DF0	bone
NCI_CGAP_DF1	bone
NCI_CGAP_DH0	uncharacterized tissue
NCI_CGAP_DH1	uncharacterized tissue
NCI_CGAP_DI0	lung
NCI_CGAP_DT0	uncharacterized tissue
NCI_CGAP_DT1	uncharacterized tissue
NCI_CGAP_ED0	uncharacterized tissue
NCI_CGAP_ED1	uncharacterized tissue
NCI_CGAP_EI0	uncharacterized tissue
NCI_CGAP_EI1	uncharacterized tissue
Homo sapiens blastocyst	uncharacterized tissue
NIH_MGC_109	ovary
NIH_MGC_107	mammary gland
UI-E-DW0	eye
UI-E-CR0	eye
UI-E-EO1	eye
UI-E-EO0	eye
UI-E-DX0	eye
UI-E-DX1	eye
UI-E-CQ0	eye
UI-E-CL0	retina
UI-E-CR1	eye
UI-E-CK1	eye
UI-E-CK0	eye
UI-E-CQ1	eye
UI-E-CI1	eye
UI-E-CI0	retina
UI-E-DW1	eye
UI-E-CL1	retina

cDNA Library	Designated Tissue for Library
UI-E-EJ1	eye
UI-E-EJ0	retina
S1SNU5	stomach
S1SNU5s1	stomach
S1SNU5s2	stomach
S2SNU668	stomach
S2SNU668s1	stomach
S3SNU16	stomach
S3SNU16s1	stomach
S4SNU1	stomach
S4SNU1s1	uncharacterized tissue
S5SNU484	stomach
S5SNU484s1	stomach
S6SNU620	stomach
S6SNU620s1	stomach
S7SNU719	stomach
S7SNU719s1	stomach
S8SNU638	stomach
S8SNU638s1	uncharacterized tissue
S9SNU601	stomach
S10SNU1	stomach
S11SNU1	stomach
S12SNU216	stomach
S12SNU216s1	uncharacterized tissue
S13KMS5	stomach
S13KMS5s1	stomach
S14K402	stomach
S14K402s1	stomach
S15N181907	uncharacterized tissue
S16N667673	uncharacterized tissue
S17N258215	stomach
S18N669761	stomach
S19N665307	stomach
S20T665307	stomach
S21SNU520	stomach
S21SNU520s1	stomach
S22SNU16	stomach
S22SNU16n1	stomach
Affymetrix mapping of human transcriptome	uncharacterized tissue
8 5 week embryo anterior tongue 8 5 EAT	head and neck

cDNA Library	Designated Tissue for Library
Human Trabecular Meshwork cDNA library	eye
Homo sapiens gastric cancer	uncharacterized tissue
Homo sapiens gastric mucosa	uncharacterized tissue
NCI_CGAP_Fs1	uncharacterized tissue
NCI_CGAP_Pl1	placenta
NCI_CGAP_Pl2	placenta
NCI_CGAP_Pl3	placenta
NCI_CGAP_Pl4	placenta
NCI_CGAP_Pl5	uncharacterized tissue
NCI_CGAP_Pl6	placenta
NCI_CGAP_Pl7	uncharacterized tissue
NCI_CGAP_Car1	uncharacterized tissue
UI-CF-EN1	lung
UI-CF-EN0	uncharacterized tissue
UI-CF-EC1	lung
UI-CF-DU1	lung
Homo sapiens primary mesangial cells	uncharacterized tissue
Homo sapiens thyroid papillary carcinoma	uncharacterized tissue
Homo sapiens thyroid adenoma	uncharacterized tissue
subtracted library of lung cancer cell	uncharacterized tissue
Human fetal skeletal muscle subtracted cDNA library	uncharacterized tissue
Homo sapiens clone	uncharacterized tissue
Homo sapiens thyroid	uncharacterized tissue
human vestibular cDNA library	uncharacterized tissue
Human SW480 Lambda gt11 cDNA Library	uncharacterized tissue
Subtracted_BTII	uncharacterized tissue
subtractive library of bladder carcinoma	uncharacterized tissue
NCI_CGAP_Ct1	uncharacterized tissue
NCI_CGAP_Ch1	bone
upregulated by angiotensin II in mesangial cells	uncharacterized tissue
pool_AK_lib_v_SPA	uncharacterized tissue
pool_Hela_lib_v_SPE	uncharacterized tissue
pool_AB_lib_v_SPB	uncharacterized tissue
pool_ALU_lib_v_SPA	uncharacterized tissue
pool_AB_lib_v_SPE	uncharacterized tissue
pool_T_lib_v_SPA	uncharacterized tissue
pool_H_lib_v_SPB	uncharacterized tissue
pool_AH_lib_v_SPA	uncharacterized tissue
pool_AK_lib_v_SPC	uncharacterized tissue
pool_T_lib_v_SPE	uncharacterized tissue

cDNA Library	Designated Tissue for Library
pool_Hela_lib_v_SPA	uncharacterized tissue
pool_AB_lib_v_SPD	uncharacterized tissue
pool_BM_lib_v_SPA	uncharacterized tissue
pool_AH_lib_v_SPC	uncharacterized tissue
pool_HL_lib_v_SPB	uncharacterized tissue
pool_T_lib_v_SPB	uncharacterized tissue
pool_AK_lib_v_SPE	uncharacterized tissue
pool_AB_lib_v_SPA	uncharacterized tissue
pool_T_lib_v_SPC	uncharacterized tissue
pool_Hela_lib_v_SPD	uncharacterized tissue
pool_YT_lib_v_SPD	uncharacterized tissue
pool_AH_lib_v_SPE	uncharacterized tissue
pool_HPB_lib_v_SPE	uncharacterized tissue
pool_HPB_lib_v_SPA	uncharacterized tissue
pool_YT_lib_v_SPA	uncharacterized tissue
pool_AK_lib_v_SPD	uncharacterized tissue
pool_HL_lib_v_SPE	uncharacterized tissue
pool_FLU_lib_v_SPD	uncharacterized tissue
pool_H_lib_v_SPC	uncharacterized tissue
pool_HL_lib_v_SPC	uncharacterized tissue
pool_HPB_lib_v_SPC	uncharacterized tissue
pool_FLU_lib_v_SPE	uncharacterized tissue
pool_AB_lib_v_SPC	uncharacterized tissue
pool_SK_lib_v_SPB	uncharacterized tissue
pool_FLU_lib_v_SPA	uncharacterized tissue
pool_HPB_lib_v_SPB	uncharacterized tissue
pool_U_lib_v_SPD	uncharacterized tissue
pool_T_lib_v_SPD	uncharacterized tissue
pool_FL_lib_v_SPB	uncharacterized tissue
pool_SK_lib_v_SPD	uncharacterized tissue
pool_FB_lib_v_SPE	uncharacterized tissue
pool_AH_lib_v_SPD	uncharacterized tissue
pool_FB_lib_v_SPB	uncharacterized tissue
pool_FL_lib_v_SPD	uncharacterized tissue
pool_AK_lib_v_SPB	uncharacterized tissue
pool_U_lib_v_SPB	uncharacterized tissue
pool_Hela_lib_v_SPB	uncharacterized tissue
pool_SK_lib_v_SPE	uncharacterized tissue
pool_SK_lib_v_SPC	uncharacterized tissue
pool_H_lib_v_SPD	uncharacterized tissue

cDNA Library	Designated Tissue for Library
pool_FLU_lib_v_SPB	uncharacterized tissue
pool_SK_lib_v_SPA	uncharacterized tissue
pool_HSI_lib_v_SPE	uncharacterized tissue
pool_FB_lib_v_SPC	uncharacterized tissue
pool_YT_lib_vec17	uncharacterized tissue
pool_YT_lib_vec20	uncharacterized tissue
pool_AH_lib_vec14	uncharacterized tissue
pool_AK_lib_vec22	uncharacterized tissue
pool_AB_lib_vec19	uncharacterized tissue
pool_FLU_lib_v_SPC	uncharacterized tissue
pool_ALU_lib_v_SPD	uncharacterized tissue
pool_FB_lib_v_SPD	uncharacterized tissue
pool_U_lib_v_SPA	uncharacterized tissue
pool_Hela_lib_v_SPC	uncharacterized tissue
pool_U_lib_v_SPE	uncharacterized tissue
pool_AH_lib_v_SPB	uncharacterized tissue
SAGE_Pancreas_carcinoma_CL_ASPC	pancreas
SAGE_Pancreas_carcinoma_CL_PL45	pancreas
Human lung squamous cell carcinoma	uncharacterized tissue
KN0015	uncharacterized tissue
KT0047	uncharacterized tissue
HB0025	uncharacterized tissue
HB0021	uncharacterized tissue
HB0022	uncharacterized tissue
HB0013	uncharacterized tissue
HB0030	uncharacterized tissue
HB0044	uncharacterized tissue
HB0039	uncharacterized tissue
HB0043	uncharacterized tissue
HB0053	uncharacterized tissue
HB0040	uncharacterized tissue
BT0823	uncharacterized tissue
BT2003	uncharacterized tissue
BT2504	uncharacterized tissue
BT3503	uncharacterized tissue
BT3502	uncharacterized tissue
BT4001	uncharacterized tissue
BT3004	uncharacterized tissue
BT2002	mammary gland
BT2000	uncharacterized tissue

cDNA Library	Designated Tissue for Library
BT2001	uncharacterized tissue
BT2501	uncharacterized tissue
BT2502	uncharacterized tissue
BT2505	uncharacterized tissue
BT2004	uncharacterized tissue
BT2503	uncharacterized tissue
BT3001	uncharacterized tissue
BT3000	uncharacterized tissue
BT3002	uncharacterized tissue
BT3003	uncharacterized tissue
BT4505	uncharacterized tissue
BT4500	uncharacterized tissue
BT3501	uncharacterized tissue
BT4502	uncharacterized tissue
BT4507	uncharacterized tissue
BT4501	uncharacterized tissue
BT4000	uncharacterized tissue
BT4503	uncharacterized tissue
BT5004	uncharacterized tissue
BT6007	uncharacterized tissue
BT5501	uncharacterized tissue
BT5505	uncharacterized tissue
BT5508	uncharacterized tissue
BT5001	uncharacterized tissue
BT6001	uncharacterized tissue
BT4508	uncharacterized tissue
BT6000	uncharacterized tissue
BT0151	uncharacterized tissue
BT1000	uncharacterized tissue
BT6005	uncharacterized tissue
BN0094	uncharacterized tissue
CT0549	uncharacterized tissue
CT0553	uncharacterized tissue
CT0829	uncharacterized tissue
CT0839	uncharacterized tissue
CT0823	uncharacterized tissue
CT0157	uncharacterized tissue
CT0821	uncharacterized tissue
CT0845	uncharacterized tissue
CT0844	uncharacterized tissue

cDNA Library	Designated Tissue for Library
CT0848	uncharacterized tissue
CT0849	uncharacterized tissue
CT0850	uncharacterized tissue
CT0830	uncharacterized tissue
CT0765	uncharacterized tissue
CT0368	uncharacterized tissue
CS0004	uncharacterized tissue
CS0029	uncharacterized tissue
CI0020	uncharacterized tissue
CI0036	uncharacterized tissue
CI0004	uncharacterized tissue
CI0106	uncharacterized tissue
CI0104	uncharacterized tissue
CI0188	uncharacterized tissue
CI0187	uncharacterized tissue
CN0194	uncharacterized tissue
CN0185	uncharacterized tissue
CN0157	uncharacterized tissue
CN0193	uncharacterized tissue
CN0186	uncharacterized tissue
CN0195	uncharacterized tissue
CN0170	uncharacterized tissue
CN0177	uncharacterized tissue
CN0163	uncharacterized tissue
CN0192	uncharacterized tissue
CN0171	uncharacterized tissue
CN0188	uncharacterized tissue
CN0190	uncharacterized tissue
CN0833	uncharacterized tissue
CN0265	uncharacterized tissue
CN0152	uncharacterized tissue
CN0168	uncharacterized tissue
CN0191	uncharacterized tissue
CN0203	uncharacterized tissue
CN0202	uncharacterized tissue
CN0175	uncharacterized tissue
CN0204	uncharacterized tissue
HT0308	uncharacterized tissue
HT1309	uncharacterized tissue
HT1196	uncharacterized tissue

cDNA Library	Designated Tissue for Library
HT1310	uncharacterized tissue
HT1308	uncharacterized tissue
HT1245	uncharacterized tissue
HT1315	uncharacterized tissue
HT1304	uncharacterized tissue
HT0624	uncharacterized tissue
HT0614	uncharacterized tissue
HN1176	uncharacterized tissue
HN0142	uncharacterized tissue
HR1000	uncharacterized tissue
RT0049	uncharacterized tissue
RT0081	uncharacterized tissue
RT0087	uncharacterized tissue
RT0086	uncharacterized tissue
ET0278	uncharacterized tissue
ET0275	uncharacterized tissue
ET0267	uncharacterized tissue
ET0261	uncharacterized tissue
MT0432	uncharacterized tissue
MT0431	uncharacterized tissue
MT0426	uncharacterized tissue
MT0429	uncharacterized tissue
MT0382	uncharacterized tissue
MT0405	uncharacterized tissue
MT0073	uncharacterized tissue
MT0404	uncharacterized tissue
MT0425	uncharacterized tissue
NN0258	uncharacterized tissue
NN0252	uncharacterized tissue
NN0256	uncharacterized tissue
NN0255	uncharacterized tissue
NN0262	uncharacterized tissue
NN0261	uncharacterized tissue
NN0257	uncharacterized tissue
NN0273	uncharacterized tissue
NN0253	uncharacterized tissue
NN0268	uncharacterized tissue
NN0259	uncharacterized tissue
NN0267	uncharacterized tissue
NN0173	uncharacterized tissue

cDNA Library	Designated Tissue for Library
NN1129	uncharacterized tissue
NN1131	uncharacterized tissue
NT0312	brain
NT0317	uncharacterized tissue
OT0237	uncharacterized tissue
OT0223	uncharacterized tissue
OT0232	uncharacterized tissue
OT0199	uncharacterized tissue
OT0227	uncharacterized tissue
OT0226	uncharacterized tissue
OT0236	uncharacterized tissue
OT0229	uncharacterized tissue
OT0244	uncharacterized tissue
OT0245	uncharacterized tissue
OT0233	uncharacterized tissue
OT0247	uncharacterized tissue
OT0251	uncharacterized tissue
OT0249	uncharacterized tissue
OT0246	uncharacterized tissue
OT0250	uncharacterized tissue
OT0259	uncharacterized tissue
OT0230	uncharacterized tissue
GN0098	uncharacterized tissue
GN0518	uncharacterized tissue
GN0228	uncharacterized tissue
GN0517	uncharacterized tissue
GN0514	uncharacterized tissue
GN0504	uncharacterized tissue
GN0510	uncharacterized tissue
GN0509	uncharacterized tissue
GN0519	uncharacterized tissue
GN0308	uncharacterized tissue
TN0137	uncharacterized tissue
TN0163	uncharacterized tissue
TN0185	uncharacterized tissue
TN0186	uncharacterized tissue
TN0194	uncharacterized tissue
TN0205	uncharacterized tissue
TN0191	uncharacterized tissue
NCI_CGAP_Ch2	bone

cDNA Library	Designated Tissue for Library
Differential display of retinoic acid receptor beta cells	uncharacterized tissue
Human embryo cerebrum cortex	uncharacterized tissue
NCI_CGAP_FH0	bone
NCI_CGAP_FE0	bone
CFS leukocyte	uncharacterized tissue
Homo sapiens pancreas and fetal brain	uncharacterized tissue
Human vestibular cDNA library	uncharacterized tissue
Human Retina cDNA (Un-normalized, unamplified): hd/he	retina
Homo sapiens library (Kratz CP)	uncharacterized tissue
Human Fat Cell 5'-Stretch Plus cDNA Library	adipose
Homo sapiens PCI-O6A; head and neck squamous cell carcinoma	uncharacterized tissue
pool_Dau_lib_v_SPB	uncharacterized tissue
pool_HSI_lib_v_SPA	uncharacterized tissue
pool_U_lib_v_SPC	uncharacterized tissue
T_lib_v_0.5M_pool	uncharacterized tissue
FLU_lib_v_0.5M_pool	uncharacterized tissue
HPB_lib_v_0.5M_pool	uncharacterized tissue
SK_lib_v_0.5M_pool	uncharacterized tissue
AK_lib_v_0.5M_pool	uncharacterized tissue
Hela_lib_v_0.5M_pool	uncharacterized tissue
AH_lib_v_0.5M_pool	uncharacterized tissue
U_lib_v_0.5M_pool	uncharacterized tissue
pool_H_lib_v_SPE	uncharacterized tissue
Homo sapiens library (Ashcroft K)	uncharacterized tissue
727 (synonym: hmcf1)	mammary gland
SAGE_OVT-7	ovary
Schneider fetal brain 00004	brain
HT0017	head and neck
HT0022	head and neck
HT0028	head and neck
NCI_CGAP_Brn50	brain
HT0010	head and neck
HT0013	head and neck
HT0019	head and neck
HT0029	head and neck
Homo sapiens testis (Singh BN)	uncharacterized tissue
Homo sapiens NB4	uncharacterized tissue
SAGE_HMEC-B41	mammary gland
SAGE_MDA453	mammary gland
SAGE_SKBR3	mammary gland

cDNA Library	Designated Tissue for Library
BT001	mammary gland
BT002	mammary gland
BT003	mammary gland
BT004	mammary gland
BT005	mammary gland
BT006	mammary gland
BT007	mammary gland
BT008	mammary gland
BT009	mammary gland
BT010	mammary gland
BT011	mammary gland
BT012	mammary gland
BT013	mammary gland
BT015	mammary gland
BT016	mammary gland
BT017	mammary gland
BT018	mammary gland
BT019	mammary gland
BT020	mammary gland
BT021	mammary gland
BT022	mammary gland
BT023	mammary gland
BT024	mammary gland
BT025	mammary gland
BT026	mammary gland
BT027	mammary gland
BT028	mammary gland
BT029	mammary gland
BT030	mammary gland
BT031	mammary gland
BT032	mammary gland
BT033	mammary gland
BT034	mammary gland
BT035	mammary gland
BT036	mammary gland
BT037	mammary gland
BT038	mammary gland
BT039	mammary gland
BT040	mammary gland
BT041	mammary gland

cDNA Library	Designated Tissue for Library
BT042	mammary gland
BT043	mammary gland
BT044	mammary gland
BT045	mammary gland
BT046	mammary gland
BT047	mammary gland
BT048	mammary gland
BT049	mammary gland
BT050	mammary gland
BT051	mammary gland
BT052	mammary gland
BT053	mammary gland
BT054	mammary gland
BT055	mammary gland
BT056	mammary gland
BT057	mammary gland
BT058	mammary gland
BT059	mammary gland
BT060	mammary gland
BT061	mammary gland
BT062	mammary gland
BT063	mammary gland
BT065	mammary gland
BT066	mammary gland
BT067	mammary gland
BT068	mammary gland
BT069	mammary gland
BT070	mammary gland
BT071	mammary gland
BT072	mammary gland
BT073	mammary gland
BT074	mammary gland
BT075	mammary gland
BT076	mammary gland
BT077	mammary gland
BT078	mammary gland
BT079	mammary gland
BT080	mammary gland
BT081	mammary gland
BT082	mammary gland

cDNA Library	Designated Tissue for Library
BT083	mammary gland
BT084	mammary gland
BT085	mammary gland
BT086	mammary gland
BT087	mammary gland
BT088	mammary gland
BT089	mammary gland
BT090	mammary gland
BT091	mammary gland
BT092	mammary gland
BT093	mammary gland
BT094	mammary gland
BT095	mammary gland
BT096	mammary gland
BT097	mammary gland
BT098	mammary gland
BT099	mammary gland
BT100	mammary gland
BT101	mammary gland
BT102	mammary gland
BT103	mammary gland
BT104	mammary gland
BT105	mammary gland
BT106	mammary gland
BT107	mammary gland
BT108	mammary gland
BT109	mammary gland
BT110	mammary gland
BT111	mammary gland
BT112	mammary gland
BT113	mammary gland
BT114	mammary gland
BT115	mammary gland
BT116	mammary gland
BT117	mammary gland
BT118	mammary gland
BT119	mammary gland
BT120	mammary gland
BT121	mammary gland
BT122	mammary gland

cDNA Library	Designated Tissue for Library
BT123	mammary gland
BT124	mammary gland
BT125	mammary gland
BT126	mammary gland
BT127	mammary gland
BT128	mammary gland
BT129	mammary gland
BT130	mammary gland
BT131	mammary gland
BT132	mammary gland
BT133	mammary gland
BT134	mammary gland
BT135	mammary gland
BT136	mammary gland
BT137	mammary gland
BT138	mammary gland
BT139	mammary gland
BT140	mammary gland
BT141	mammary gland
BT142	mammary gland
BT143	mammary gland
BT144	mammary gland
BT145	mammary gland
BT146	mammary gland
BT147	mammary gland
BT148	mammary gland
BT149	mammary gland
BT150	mammary gland
BT152	mammary gland
BT153	mammary gland
BT154	mammary gland
BT155	mammary gland
BT156	mammary gland
BT157	mammary gland
BT158	mammary gland
BT159	mammary gland
BT160	mammary gland
BT161	mammary gland
BT162	mammary gland
BT163	mammary gland

cDNA Library	Designated Tissue for Library
BT164	mammary gland
BT165	mammary gland
BT166	mammary gland
BT168	mammary gland
BT169	mammary gland
BT170	mammary gland
BT171	mammary gland
BT172	mammary gland
BT173	mammary gland
BT174	mammary gland
BT175	mammary gland
BT176	mammary gland
BT177	mammary gland
BT178	mammary gland
BT180	mammary gland
BT181	mammary gland
BT182	mammary gland
BT183	mammary gland
BT184	mammary gland
BT185	mammary gland
BT186	mammary gland
BT187	mammary gland
BT188	mammary gland
BT189	mammary gland
BT190	mammary gland
BT191	mammary gland
BT192	mammary gland
BT193	mammary gland
BT194	mammary gland
BT195	mammary gland
BT196	mammary gland
BT197	mammary gland
BT198	mammary gland
BT199	mammary gland
BT200	mammary gland
BT201	mammary gland
BT202	mammary gland
BT203	mammary gland
BT204	mammary gland
BT208	mammary gland

cDNA Library	Designated Tissue for Library
BT209	mammary gland
BT210	mammary gland
BT211	mammary gland
BT212	mammary gland
BT213	mammary gland
BT214	mammary gland
BT215	mammary gland
BT216	mammary gland
BT217	mammary gland
BT218	mammary gland
BT219	mammary gland
BT220	mammary gland
BT221	mammary gland
BT223	mammary gland
BT224	mammary gland
BT225	mammary gland
BT226	mammary gland
BT228	mammary gland
BT229	mammary gland
BT230	mammary gland
BT232	mammary gland
BT233	mammary gland
BT234	mammary gland
BT235	mammary gland
BT236	mammary gland
BT237	mammary gland
BT238	mammary gland
BT239	mammary gland
BT240	mammary gland
BT241	mammary gland
BT242	mammary gland
BT243	mammary gland
BT244	mammary gland
BT245	mammary gland
BT246	mammary gland
BT247	mammary gland
BT248	mammary gland
BT249	mammary gland
Homo sapiens articular cartilage	uncharacterized tissue
Homo sapiens lung fetus	uncharacterized tissue

cDNA Library	Designated Tissue for Library
Daudi lymphoma	uncharacterized tissue
BT0103	mammary gland
BT0107	mammary gland
BT0220	mammary gland
CT0012	colon
CT0013	colon
CT0015	colon
CT0018	colon
CT0029	colon
CT0030	colon
CT0031	colon
CT0032	colon
CT0033	colon
CT0034	colon
CT0035	colon
CT0036	colon
CT0037	colon
CT0038	colon
CT0039	colon
CT0040	colon
CT0042	colon
CT0044	colon
CT0046	colon
CT0047	colon
CT0048	colon
CT0050	colon
CT0052	colon
CT0054	colon
CT0070	colon
CT0071	colon
CT0075	colon
CT0076	colon
HT0009	head and neck
ST0023	stomach
ST0024	stomach
ST0027	stomach
ST0028	stomach
ST0029	stomach
ST0039	stomach
ST0040	stomach

cDNA Library	Designated Tissue for Library
ST0043	stomach
ST0050	stomach
Homo sapiens HTori-3, control line	uncharacterized tissue
Homo sapiens HTG9	uncharacterized tissue
Homo sapiens HTG8	uncharacterized tissue
Homo sapiens HTG13	uncharacterized tissue
Homo sapiens HTA4	uncharacterized tissue
Homo sapiens HTG2	uncharacterized tissue
Lambda gt10 human fetal brain	uncharacterized tissue
Homo sapiens library (Hawthorn LA)	uncharacterized tissue
Homo sapiens milk	uncharacterized tissue
Homo sapiens foreskin epidermal newborn	uncharacterized tissue
Homo sapiens osteosarcoma differential display	uncharacterized tissue
SAGE_Duke_HMVEC	vascular
SAGE_Duke_HMVEC+VEGF	vascular
SAGE_mammary_epithelium	mammary gland
SAGE_DCIS	mammary gland
SAGE_Duke_757	brain
SAGE_normal_cerebellum	cerebellum
Homo sapiens larynx adult	uncharacterized tissue
Homo sapiens astrocytoma library	uncharacterized tissue
Homo sapiens non-tumorogenic library	uncharacterized tissue
Homo sapiens glioblastoma library	uncharacterized tissue
Homo sapiens library (Scherer SW)	uncharacterized tissue
NCI_CGAP_Co22	colon
NCI_CGAP_Lu34	lung
NCI_CGAP_Ov39	ovary
NCI_CGAP_Ov40	ovary
Homo sapiens liver fetus	uncharacterized tissue
U937	uncharacterized tissue
Homo sapiens muscle adult	uncharacterized tissue
Homo sapiens differential display PCR (Xian B)	uncharacterized tissue
Homo sapiens ATCC SW1088	uncharacterized tissue
NCI_CGAP_HN7	head and neck
NCI_CGAP_HN8	head and neck
NCI_CGAP_HN9	head and neck
NCI_CGAP_HN10	head and neck
NCI_CGAP_HN12	head and neck
SAGE_OVT-8	ovary
NCI_CGAP_Sub1	uncharacterized tissue

cDNA Library	Designated Tissue for Library
NCI_CGAP_Sub2	uncharacterized tissue
Synovial fibroblasts, rheumatoid arthritis	uncharacterized tissue
prostate cancer cell line LNCaP	uncharacterized tissue
Homo sapiens cell line K562 cDNA Library	uncharacterized tissue
Homo sapiens library (Jangbo Y)	uncharacterized tissue
Lambda gt10 human adult liver	uncharacterized tissue
18p11 cDNA selection library	uncharacterized tissue
SAGE_Duke_96-349	mammary gland
SAGE_Duke_40N	mammary gland
SAGE_Duke_48N	mammary gland
NCI_CGAP_Lu34.1	uncharacterized tissue
SAGE_A2780-9	ovary
Soares_NHCe_cervix	cervix
Soares_NHCeC_cervical_tumor	cervix
Homo sapiens 530 melanoma	uncharacterized tissue
Homo sapiens MV3 melanoma	uncharacterized tissue
BT000	mammary gland
Homo sapiens EB1	uncharacterized tissue
normal muscle cDNA subtractive library	uncharacterized tissue
FSHD affected muscle cDNA subtractive library	uncharacterized tissue
NCI_CGAP_HN11	head and neck
761 (synonym: hamy2)	brain
762 (synonym: hmel2)	skin
UPC15	uncharacterized tissue
DNC15	whole blood
NCI_CGAP_Li8	liver
NCI_CGAP_Lu21	lung
Homo sapiens colon carcinoma	uncharacterized tissue
SAGE_ML10-10	ovary
BT0081	mammary gland
BT0163	mammary gland
BT0168	mammary gland
BT0169	mammary gland
BT0229	mammary gland
BT0234	mammary gland
CT0002	colon
CT0003	colon
CT0004	colon
CT0005	colon
CT0016	colon

cDNA Library	Designated Tissue for Library
CT0041	colon
CT0060	colon
CT0062	colon
CT0064	colon
CT0065	colon
CT0069	colon
CT0077	colon
CT0078	colon
CT0079	colon
CT0080	colon
CT0081	colon
CT0082	colon
CT0083	colon
CT0088	colon
CT0090	colon
CT0092	colon
CT0100	colon
CT0101	colon
CT0103	colon
CT0104	colon
CT0105	colon
HT0026	head and neck
HT0027	head and neck
ST0018	stomach
ST0020	stomach
ST0031	stomach
ST0041	stomach
ST0056	stomach
PT-PCR products	uncharacterized tissue
human ovarian cancer cDNA	uncharacterized tissue
KRIBB Human TN intrathymic T-cell cDNA library	uncharacterized tissue
KRIBB Human DP intrathymic T-cell cDNA library	uncharacterized tissue
KRIBB Human CD4 intrathymic T-cell cDNA library	uncharacterized tissue
RT-PCR products human melanoma	uncharacterized tissue
NEUROBLASTOMA MYCN	uncharacterized tissue
hEx1 library	uncharacterized tissue
Homo sapiens skov	uncharacterized tissue
Homo sapiens CGL3	uncharacterized tissue
NIH_MGC_71	Uterus

cDNA Library	Tissue Category
NIH_MGC_56	Brain
NIH_MGC_87	Breast
NIH_MGC_12	Cervix
NIH_MGC_116	Colon
NIH_MGC_15	Colon
NIH_MGC_65	Colon
NIH_MGC_88	Duodenum
NIH_MGC_43	Eye
NIH_MGC_16	Eye
NIH_MGC_67	Eye
NIH_MGC_74	Heart
NIH_MGC_75	Kidney
NIH_MGC_58	Kidney
NIH_MGC_89	Kidney
NIH_MGC_45	Kidney
NIH_MGC_14	Kidney
NIH_MGC_76	Liver
NIH_MGC_90	Liver
NIH_MGC_100	Liver
NIH_MGC_77	Lung
NIH_MGC_122	Lung
NIH_MGC_69	Lung
NIH_MGC_18	Lung
NIH_MGC_68	Lung
NIH_MGC_59	Lung
NIH_MGC_7	Lung
NIH_MGC_8	Lymph
NIH_MGC_85	Lymph
NIH_MGC_99	Lymph
NIH_MGC_17	Muscle
NIH_MGC_66	Ovary
NIH_MGC_9	Ovary
NIH_MGC_125	Ovary
NIH_MGC_78	Pancreas
NIH_MGC_120	Pancreas
NIH_MGC_39	Pancreas
NIH_MGC_110	Pancreas
NIH_MGC_42	Pancreas
NIH_MGC_70	Pancreas
NIH_MGC_79	Placenta
NIH_MGC_10	Placenta

cDNA Library	Tissue Category
NIH_MGC_21	Placenta
NIH_MGC_48	Primary
NIH_MGC_83	Prostate
NIH_MGC_60	Prostate
NIH_MGC_91	Prostate
NIH_MGC_40	Prostate
NIH_MGC_81	Skeletal
NIH_MGC_41	Skin
NIH_MGC_112	Skin
NIH_MGC_49	Skin
NIH_MGC_62	Skin
NIH_MGC_20	Skin
NIH_MGC_72	Skin
NCI_CGAP_Skn3	Skin
NCI_CGAP_Skn4	Skin
NIH_MGC_113	Spleen
NIH_MGC_82	Testis
NIH_MGC_97	Testis
NIH_MGC_61	Testis
NIH_MGC_92	Testis
NIH_MGC_44	Uterus
NIH_MGC_46	Uterus
NIH_MGC_71	Uterus
NIH_MGC_84	Adrenal gland
NIH_MGC_36	B-cell
NIH_MGC_37	B-cell
NIH_MGC_38	B-cell
NIH_MGC_48	B-cell
NIH_MGC_50	B-cell
NIH_MGC_51	B-cell
NIH_MGC_52	B-cell
NIH_MGC_53	Bladder
NIH_MGC_93	Bladder
NIH_MGC_2	Blood
NIH_MGC_106	Blood
NIH_MGC_86	Bone
NIH_MGC_54	Bone marrow
NIH_MGC_55	Bone marrow
NIH_MGC_19	Brain/CNS
NIH_MGC_47	Brain/CNS
NIH_MGC_56	Brain/CNS

cDNA Library	Tissue Category
NIH_MGC_57	Brain/CNS
NIH_MGC_73	Brain/CNS
NIH_MGC_95	Brain/CNS
NIH_MGC_96	Brain/CNS
NIH_MGC_98	Brain/CNS
NIH_MGC_114	Brain/CNS
NIH_MGC_119	Brain/CNS
NIH_MGC_121	Brain/CNS
NIH_MGC_124	Brain/CNS
NIH_MGC_87	Breast
NIH_MGC_107	Breast
NIH_MGC_4	Cervix
NIH_MGC_5	Cervix
NIH_MGC_12	Cervix
NIH_MGC_35	Cervix
NIH_MGC_64	Cervix
NIH_MGC_15	Colon
NIH_MGC_65	Colon
NIH_MGC_16	Eye
NIH_MGC_43	Eye
NIH_MGC_67	Eye
NIH_MGC_102	Head/neck
NIH_MGC_74	Heart
NIH_MGC_14	Kidney
NIH_MGC_45	Kidney
NIH_MGC_58	Kidney
NIH_MGC_75	Kidney
NIH_MGC_89	Kidney
NIH_MGC_108	Kidney
NIH_MGC_118	Leukocyte
NIH_MGC_76	Liver
NIH_MGC_90	Liver
NIH_MGC_100	Liver
NIH_MGC_7	Lung
NIH_MGC_18	Lung
NIH_MGC_59	Lung
NIH_MGC_68	Lung
NIH_MGC_69	Lung
NIH_MGC_77	Lung
NIH_MGC_101	Lung
NIH_MGC_3	Lymph

cDNA Library	Tissue Category
NIH_MGC_8	Lymph
NIH_MGC_63	Lymph
NIH_MGC_85	Lymph
NIH_MGC_99	Lymph
NIH_MGC_17	Muscle
NIH_MGC_81	Muscle
NIH_MGC_9	Ovary
NIH_MGC_66	Ovary
NIH_MGC_109	Ovary
NIH_MGC_125	Ovary
NIH_MGC_39	Pancreas
NIH_MGC_42	Pancreas
NIH_MGC_70	Pancreas
NIH_MGC_78	Pancreas
NIH_MGC_110	Pancreas
NIH_MGC_10	Placenta
NIH_MGC_21	Placenta
NIH_MGC_79	Placenta
NIH_MGC_147	Placenta
NIH_MGC_115	Pooled
NIH_MGC_116	Pooled
NIH_MGC_120	Pooled
NIH_MGC_122	Pooled
NIH_MGC_123	Pooled
NIH_MGC_126	Pooled
NIH_MGC_127	Pooled
NIH_MGC_128	Pooled
NIH_MGC_141	Pooled
NIH_MGC_142	Pooled
NIH_MGC_146	Pooled
NIH_MGC_40	Prostate
NIH_MGC_60	Prostate
NIH_MGC_83	Prostate
NIH_MGC_91	Prostate
NIH_MGC_111	Prostate
NIH_MGC_20	Skin
NIH_MGC_41	Skin
NIH_MGC_49	Skin
NIH_MGC_62	Skin
NIH_MGC_72	Skin
NIH_MGC_112	Skin

cDNA Library	Designated Tissue for Library
NIH_MGC_84	Adrenal cortex, carcinoma
NIH_MGC_84	Adrenal
NIH_MGC_53	Bladder
NIH_MGC_93	Bladder
NIH_MGC_118	Blood
NIH_MGC_106	Blood
NIH_MGC_55	Bone
NIH_MGC_54	Bone
NIH_MGC_86	Bone
NIH_MGC_73	Brain
NIH_MGC_115	Brain
NIH_MGC_119	Brain
NIH_MGC_114	Brain
NCI_CGAP_Brn67	Brain
NIH_MGC_98	Brain
NIH_MGC_121	Brain
NIH_MGC_57	Brain
NCI_CGAP_Brn64	Brain
NIH_MGC_124	Brain
NIH_MGC_95	Brain
NIH_MGC_96	Brain
NIH_MGC_19	Brain
NIH_MGC_47	Brain
NIH_MGC_88	Small intestine
NIH_MGC_113	Spleen
NIH_MGC_61	Testis
NIH_MGC_82	Testis
NIH_MGC_92	Testis
NIH_MGC_97	Testis
NIH_MGC_44	Uterus
NIH_MGC_46	Uterus
