# Supplementary material for: Variation in alternative splicing across human tissues
Source: Genome Biol. 2004 Sep 13;5(10):R74. doi: 10.1186/gb-2004-5-10-r74 (PMC545594; doi:10.1186/gb-2004-5-10-r74)

Figure S2. The average length in bases of ESTs stringently aligned to gene regions across tissues. Error bars are 1 standard deviation.

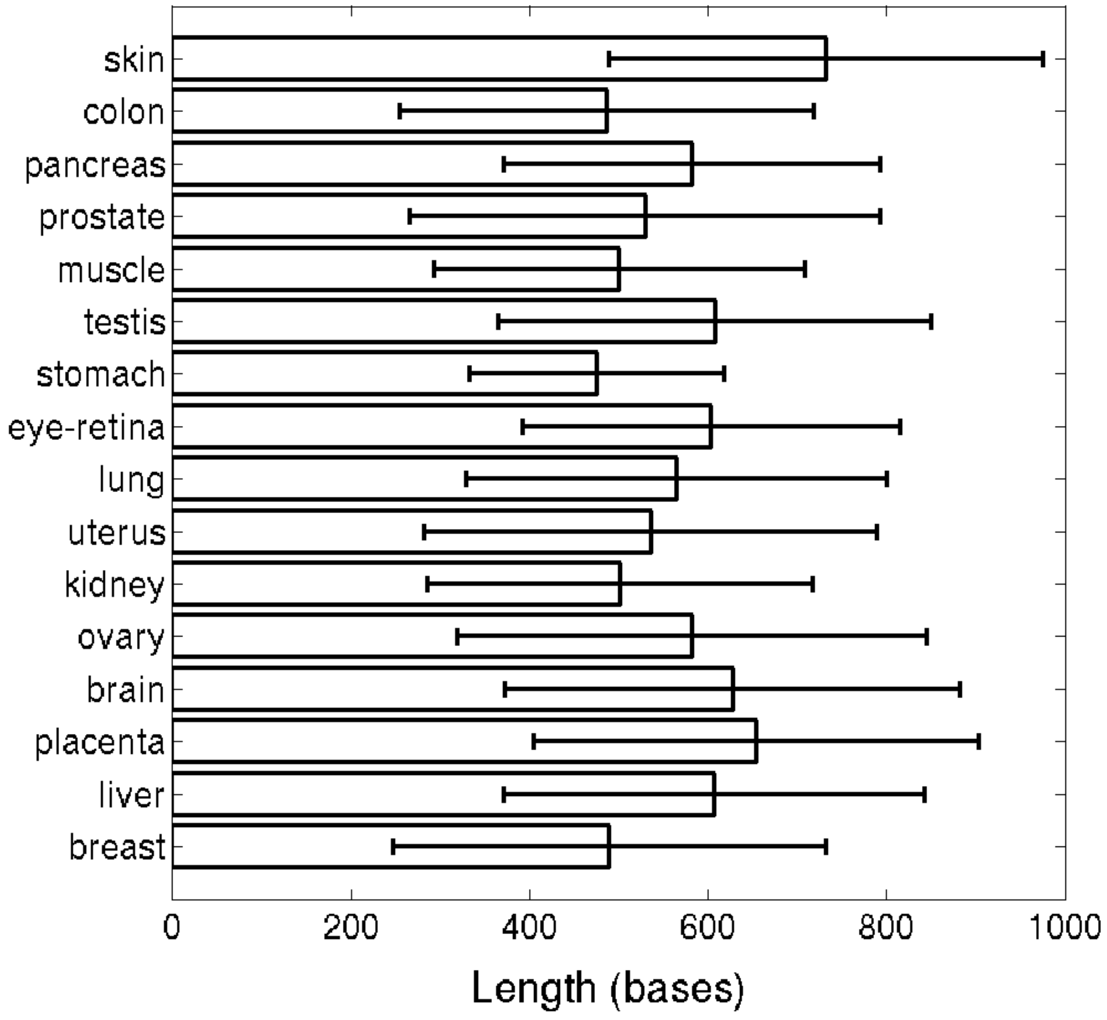

Supplement: Additional data file 6 — The average lengths of ESTs that aligned to gene loci expressed in different tissues [file gb-2004-5-10-r74-s6.pdf]
