# Supplementary material for: Variation in alternative splicing across human tissues
Source: Genome Biol. 2004 Sep 13;5(10):R74. doi: 10.1186/gb-2004-5-10-r74 (PMC545594; doi:10.1186/gb-2004-5-10-r74)
Supplement: Additional data file 7 — Human splicing factors of SR, SR-related and hnRNP genes, corresponding Ensembl gene numbers and Affymetrix microarray probe identification numbers [file gb-2004-5-10-r74-s7.pdf]

Ensembl ID	Splicing factor	HG-95A probe ID	HG-133A probe ID
136450	ASF/SF2	36098_at	211784_s_at
167978	SRm300	32761_at	207435_s_at
161547	SC35	36111_s_at	200753_x_at
100650	SRp40	40453_s_at	212266_s_at
124193	SRp55	35808_at	208804_s_at
111786	SRp30c	32573_at	201698_s_at
115875	9G8	32165_at	213649_at
116754	Srp54	32183_at	200685_at
136527	SFRS10	140_s_at	210180_s_at
112081	SRp20	351_f_at	208673_s_at
135486	hnRNP A1	31463_s_at	213356_x_at
122566	hnRNP A2/B2	36654_s_at	205292_s_at
092199	hnRNP C	32408_s_at	200751_s_at
138669	hnRNP D	38016_at	2000073_s_at
147274	hnRNP G	39731_at	213762_x_at
169045	hnRNP H1	41292_at	213472_at
165119	hnRNP K	39415_at	200775_s_at
104824	hnRNP L	35201_at	202072_at
099783	hnRNP M	37717_at	2000072_s_at
125970	hnRNP RALY	36125_s_at	201271_s_at

Table S5. **Human splicing factors of SR, SR-related and hnRNP protein families.** The last six digits of the Ensembl gene number (`ENSG00000#`) are in the first column, followed by the gene name and corresponding Affymetrix DNA microarray HG-95A and HG-133A probe identification numbers..
