# Supplementary material for: Variation in alternative splicing across human tissues
Source: Genome Biol. 2004 Sep 13;5(10):R74. doi: 10.1186/gb-2004-5-10-r74 (PMC545594; doi:10.1186/gb-2004-5-10-r74)
Supplement: Additional data file 8 — The distribution of the average Pearson correlation coefficient values across different tissues for expression levels of random sets of genes obtained from the Affymetrix microarray datat [file gb-2004-5-10-r74-s8.pdf]

Supplementary Figure 3

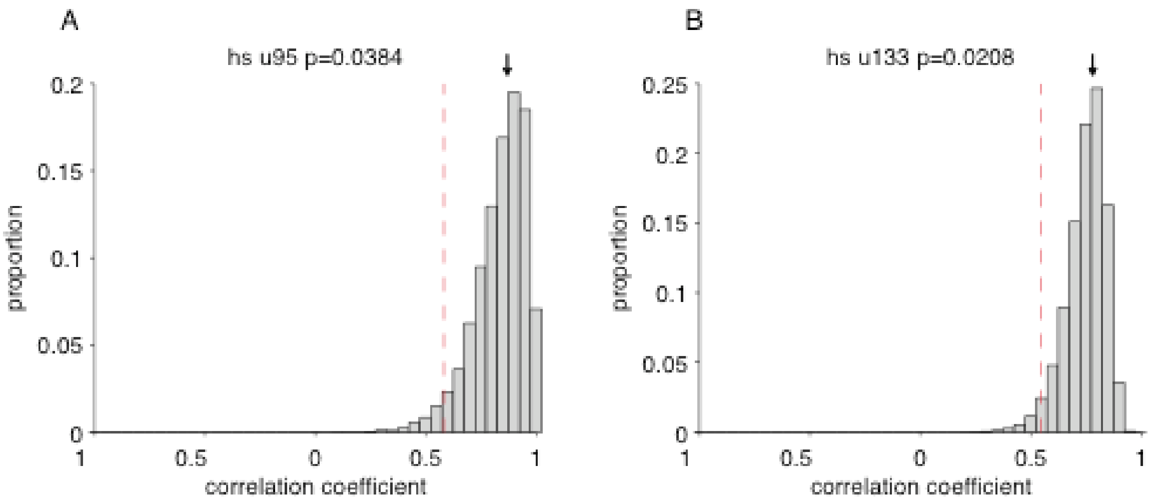

Distribution of mean correlation coefficients for random sets of genes. For each iteration (total of 1000 iterations), we selected 20 random genes and computed the correlation coefficient between the expression levels in 26 different tissues as in Figure 3 for the U95A chip (A) and the U133A chip (B). The figure shows the distribution of the average correlation coefficient for each tissue. The arrow indicates the mean value. The dashed vertical line shows the mean correlation coefficient obtained in Figure 3 for the liver tissue. The *p*-value above each subplot shows the proportion of values that were less than the observed value for the liver (total number of coefficients in the distribution = 1000 iterations x 26 tissues).
